# Does cereal, protein and micronutrient availability hold the key to the malnutrition conundrum? An exploratory analysis of cereal cultivation and wasting patterns of India

**DOI:** 10.12688/wellcomeopenres.15934.1

**Published:** 2020-06-02

**Authors:** Rama Krishna Sanjeev, Prashanth Nuggehalli Srinivas, Bindu Krishnan, Yogish Channa Basappa, Akshay S. Dinesh, Sabu K. Ulahannan

**Affiliations:** 1 Pediatrics, Rural Medical College, Pravara Institute of Medical Sciences, Loni (BK), Ahmednagar district, Maharashtra, 413736, India; 2 Health equity cluster, Institute of Public Health Bengaluru, Bengaluru, Karnataka, 560070, India; 3 Physiology, Rural Medical College, Pravara Institute of Medical Sciences, Loni (BK), Ahmednagar district, Maharashtra, 413736, India; 4 Metastring Foundation, Bengaluru, Karantaka, 560020, India

**Keywords:** Millets, malnutrition, wasting, stunting, MTORC1(mammalian target of Rapamycin complex1), GCN2( general control non derepressible 2), DSCQ( District subsistence cultivation quantum)

## Abstract

**Background: **High prevalence of maternal malnutrition, low birth-weight and child malnutrition in India contribute substantially to the global malnutrition burden. Rural India has disproportionately higher levels of child malnutrition. Stunting and wasting are the primary determinants of malnutrition and their district-level distribution shows clustering in different geographies and regions.

**Methods: **The last round of National Family Health Survey (NFHS4) has disaggregated data by district, enabling a more nuanced understanding of the prevalence of markers of malnutrition. We used data from NFHS4 and agricultural statistics datasets to analyse relationship of cereal cultivation with the prevalence of child malnutrition. We studied the current science on growth-related nutrient-sensing pathways to explain this pattern.

**Results: **Stunting and wasting patterns across districts show a distinct geographical and age distribution; districts with higher wasting showed early prevalence of 40% at six months of age. Wasting was associated with higher cultivation of millets, with a stronger association seen for jowar and other millets. Low maternal BMI in districts with higher wasting could be linked to the consumption of millets as staple. We conceptualised a hypothetical schematic pathway linking early origin of wasting in children with millet-based diet, driven by inhibition of critical intra-cellular pathways controlling growth covering pre-natal, post-natal and early childhood. The analysis was limited by lack of fine-scale data on prevalence of low birth-weight and type of cereal consumed.

**Conclusions: **Multi-site observational studies of long-term effects of type of cereals consumed could help explain the ecogeographic distribution of malnutrition in India. Cereals, particularly millets constitute the bulk of protein intake among the poor, especially in rural areas in India where wasting persists. Policies and programs targeting malnutrition need to address type of cereal consumed in order to impact childhood malnutrition in parts of India where subsistence cultivation of millets for staple consumption is prevalent.

## Introduction

Globally, the World Health Organization (WHO) estimates that among children under five, about 151 million suffer from stunting and 51 million from wasting with consequent risks of mortality, morbidity and development. The latest stunting trends indicate increases in Africa along with substantial reductions across Asia. However, with regards to wasting, South Asia accounts for half of all wasted children globally ^1^. Stunting (low height for age), mid upper-arm circumference (MUAC) less than 125 mm and wasting (low weight for height) are the primary measures of prevalence of under-5 malnutrition ^2, 3^. Children with stunting and wasting both have reduction in muscle mass, albeit greater in the latter, which reduces the pool of available alanine and glutamine for gluconeogenesis, which in turn is essential for supply of glucose to the brain for functioning. Children with wasting also have reduced fat mass, which contributes to a depressed immune response through leptin ^4^. Children having both stunting and wasting have the greatest risk of mortality. Indeed both are interlinked; wasting earlier in infancy contributes to stunting within a few months to a year ^5^. Nationwide surveys, in fact, often reveal a mix of both in any population. Further, children with both stunting and wasting are the most vulnerable to mortality due to infections, and hence regions with high prevalence of stunting and wasting are likely to also report higher infant mortality ^6, 7^. India has a disproportionately high prevalence of stunting; there are 62 million stunted children accounting for 40% of the global total, despite having 20% of the world population ^8, 9^. The socio-economic gains and poverty reduction of the past decades have not translated into commensurate reduction of stunting and wasting in children, often characterised as, the Asian enigma ^10– 12^.

### Early onset of malnutrition in India

Historically distinct forms of severe malnutrition referred to as Kwashiorkor and Marasmus are today captured under the umbrella term severe acute malnutrition (SAM). SAM is recognised to have a range of clinical manifestations with a multifactorial causation ^13^. While some of the risk factors of malnutrition are proximate, related to maternal and household characteristics, distal factors related to the wider socio-economic environment have also been attributed ^2, 14^. Poverty, poor sanitation and hygiene, in and around households, are important social determinants that can act across households and geographies causing clustering of malnutrition in entire regions. Ultimately, the proximate causes of morbidity and mortality in severe malnutrition could be recurrent infections probably culminating in environmental enteric dysfunction (EED) with contributions from household nutritional factors (including breast feeding and complementary feeding) and maternal nutritional status (preconceptionally, as well as during pregnancy and breastfeeding) ^2, 15– 17^. Pre-conceptional low maternal BMI is an important contributor to intrauterine growth retardation. Maternal short stature, even after adjustment for socio-economic status, is also associated with low birth weight, child stunting, delivery complications and increased child mortality ^18^.

In India (and other parts of South Asia as well), low birth weight and early wasting (during the first six months of life) has been shown to be a particular feature of malnutrition, in contrast to other low- and middle-income countries (LMIC) ^12, 19^. Notwithstanding precise estimates being unavailable due to lack of disaggregated data, India has disproportionately high low birth weight prevalence ^20, 21^. Since greater than 50% of the foetal and neonatal energy consumption is by the brain, the impact of malnutrition in infancy on the developing brain is significant, with consequences for school preparedness and adult life as well ^22^. Nutritional and socio-economic drivers of poor maternal nutrition include lack of maternal milk intake during pregnancy in a predominantly vegetarian cereal based diet ^23^ and low protein diet in late pregnancy ^24^. Maternal malnutrition adversely affects key nutrients in breast milk including vitamins B1, B2, B6, B12, A, D as well as selenium, phosphorous, choline, iodine, free amino acids and fatty acids ^15, 25– 27^. Maternal multiple micronutrient deficiencies can cause an adverse impact on both the foetus and breastfeeding infant ^28^.

### Subsistence farming and millet dependence

Indian states consist of 640 districts (at the time of NFHS4) with wide differences in geography, climate and the main agricultural crops. India has a large and poor rural population (68.9% rural with 25.5 % rural poverty prevalence), and over half (54%) of the working rural population (481.9 million) are cultivators and agricultural labourers ^29, 30^. Small land-holding farmers (owning less than 2 hectares of land) and their families constitute more than half the country’s population. Only half (96.46 million hectares) of the total area under cultivation (198.36 million hectares) is irrigated ^31^. Although, rice and wheat together constitute 75% of total area under food grain cultivation, Jowar (Sorghum) and Bajra (pearl millet) make up a significant 13.8%. However, the distribution of food grain cultivation in irrigated land varies, with rice (60%) and wheat (94.2%), expectedly being grown largely on irrigated land. In contrast, Sorghum (Jowar) and Pearl millet (Bajra) are grown largely in non-irrigated lands, most likely by small land-holding farmers in monsoon-dependent arid or semi-arid regions of the country, which are also among the poorest ^32, 33^. Household food grain consumption and diets in such regions are likely driven by these strong linkages between agro-climatic and eco-geographic factors, more so among poorer households with socio-economic barriers to achieve dietary diversity.

The latest completed round of National Family Health Survey 4 (NFHS 4) was published in 2015 with district-level data for the first time ^34^. Based on unpublished field observations of wasting prevalence among populations depending on millet as staple in rural Maharashtra (spanning western and central India), we critically examined the spatial patterns of prevalence of stunting and wasting at the district level across India with the objective of exploring the role of dietary staple cereal consumption pattern as a possible explanation for these patterns. Further, we propose a hypothetical theoretical framework that integrates evidence emerging from agro-climatic and geographic patterns with physiological mechanisms of malnutrition.

## Methods

We analysed district-level secondary data on under-5 stunting, wasting, short stature and low body mass index (BMI) of women of the 15–49 years age group with crop cultivation to assess geo-spatial overlaps and analyse relationships between malnutrition and subsistence cultivation of millets. We used available evidence on cellular pathways to malnutrition to arrive at a plausible explanatory framework for these patterns.

### Definitions and data sources

We considered the following millet crops widely grown and reported in Indian agriculture databases and the Directorate of Millets Development (under Department of Agriculture, Co-operation and Farmers Welfare) in our analysis, henceforth mentioned as millets: jowar (sorghum), bajra (pearl millet) and other millets (Kodo millet, little millet, proso millet, barnyard millet and foxtail millet). The initial hypothesis of the association between children presenting with severe malnutrition and millets was in locations with staple consumption of millets other than ragi (finger millet). Ragi was excluded from above because it has a relatively better nutritional profile and belongs to a distinct sub-family in the grass family *Poaceae* ^35– 37^ We did not include Maize in the analysis as, in India, only 20% of maize is consumed, with remaining being utilised for other purposes ^38^.

We adopted the definitions of districts with high prevalence of wasting and stunting from district-level malnutrition analysis by Junaid and Mohanty ^39^, which has considered >45% district-level stunting prevalence, and >27% district-level wasting prevalence as high. We used levels of > = 30% and >= 15% as high prevalence of women’s BMI (<18.5) and short stature(< 145 cms) respectively.

We extracted variables of interest from round four of the National Family Health Survey (NFHS4). NFHS4 is a standardised and periodic nationally representative survey covering 601,509 households, 699,686 women aged 15–49 years and 103,525 men aged 15–54 years that provides comprehensive data on various aspects of maternal and child health ^39, 40^. NFHS-4 provides unit level data (for each of the 640 districts of India at the time of survey) for download upon request via the demographic health survey data repository ^40, 41^. We extracted data on population of each district from the 2011 Census ^30^.

For data on cultivation of cereal crops, we used DACNET, a web-based land use statistics information system maintained by the Agriculture Informatics Division of the National Informatics Centre of the Government of India ^42^.

From each of the three data sources mentioned above, the following data were extracted to prepare a district-level dataset for analysis ^43^:

1. From the 2011 census data, district-wise total population
2. From the NFHS4 data,
  a. using appropriate weights district averages of BMI of women 15–49 years age group and district-level prevalence of short stature was calculated from women’s dataset
  b. district-level prevalence of wasting and stunting was calculated from the children’s dataset
  c. Proportion of people in wealth quintiles for a given district was calculated from household dataset
3. Various crop data is available in state-wise reports compiled by the Ministry of Agriculture and Farmers Welfare. We extracted district-level area under cultivation of cereals: rice, wheat, maize, ragi and millets (by type as defined above) into a spreadsheet. Data was from the latest state-wise reports available at the time of analysis at DACNET ^42^ (data for most states ranged for years between 2014–17 except Maharashtra 2002–03, Manipur 2004–05 and Gujarat 2007–08; all data in hectares converted to acres).

Using district names as the common variable in all three datasets, they were merged. Any errors due to district spellings and duplicate district names across differing states were handled with caution to ensure proper merging. For each district we estimated the population of poor by multiplying the census figures for population of the district by the proportion of the population in the fourth and fifth wealth quintiles (from NFHS4). This was based on the assumption that subsistence millet consumption is largely restricted to poor small land-holding farmers ^44, 45^. We formulated a measure, the District Subsistence Cultivation Quantum (DSCQ) for each cereal by multiplying the per-capita area under cultivation of cereal (log-normalised) with the population of poor as estimated above.

We searched literature on PubMed reporting on cellular pathways and metabolomics pertaining to child malnutrition from 1990 onwards, particularly looking for those that could explain our hypothesised pattern of early origins of wasting in children in relation to millet staple. We used combinations of the following keywords: mammalian target of rapamycin, general control non derepressible 2, nutrient-sensing pathways, amino-acids, intrauterine growth retardation, milk, micronutrients, stunting, wasting. We retained articles that were relevant to explaining early onset of malnutrition, especially in the first 1000 days of life (excluding articles dealing with other stages of life and other health problems). We identified 72 papers that were relevant to our search, of which seven papers were integrated into the schematic pathway we describe below. The pathway integrates cellular mechanisms with nutritional and other socio-environmental influences in the first 1000 days of life.

### Variables

Variables from both data sources (NFHS-4 and agriculture cultivation data) were combined into a single dataset ^43^.

***Independent variables.*** We used per capita area under cultivation of crops and the DSCQ for each cereal. Except for areas in economically better-off and well-irrigated regions, particularly in northern India ^44, 46^, where bajra and jowar are grown for animal fodder and other purposes; elsewhere, millets are cultivated largely by small land-holding rural poor farmers and for subsistence purposes ^47^.

**Outcome variables:** percentage of children with wasting (weight for height <2SD), percentage of children with stunting (height for age <2 SD), prevalence of low body mass index (<18.5) among women in 15–49 years age-group and short stature(<145 cm height) among women in 15–49 age-group.

### Analysis

***Spatial malnutrition patterns.*** We assessed overlaps between high prevalence of stunting and/or wasting with cereal cultivation data by generating maps derived from The Database of Global Administrative Areas (GADM) ^48^. We merged tabular data (from a spreadsheet file) with geographic data (from a geojson file), chose variables of interest, created map legends dynamically and rendered multiple maps using a custom-built wrapper software written in javascript which internally uses Mapbox GL JS library (version 1.10.0) for rendering maps ^49^. Further information on what this software wrapper does and how it works is present in the README file of the source code ^50^. As a base layer, DSCQ was shaded using a linear interpolator with manually chosen colour levels for the legend. A transparent layer of outcome variables (stunting and wasting, low BMI and short stature) marked with distinct stripe patterns was overlaid on the base layer for visualizing overlap.

***Examining relationship between subsistence millet cultivation, childhood malnutrition and its early onset.*** For each cereal, we examined its association with district-level prevalence of stunting and wasting, as well as the association between maternal factors (women’s BMI and short stature) and DSCQ (normalised using logarithmic transformation) by linear regression. We examined the relationship of age with wasting and stunting at the district level.

## Results

**Figure 1.**
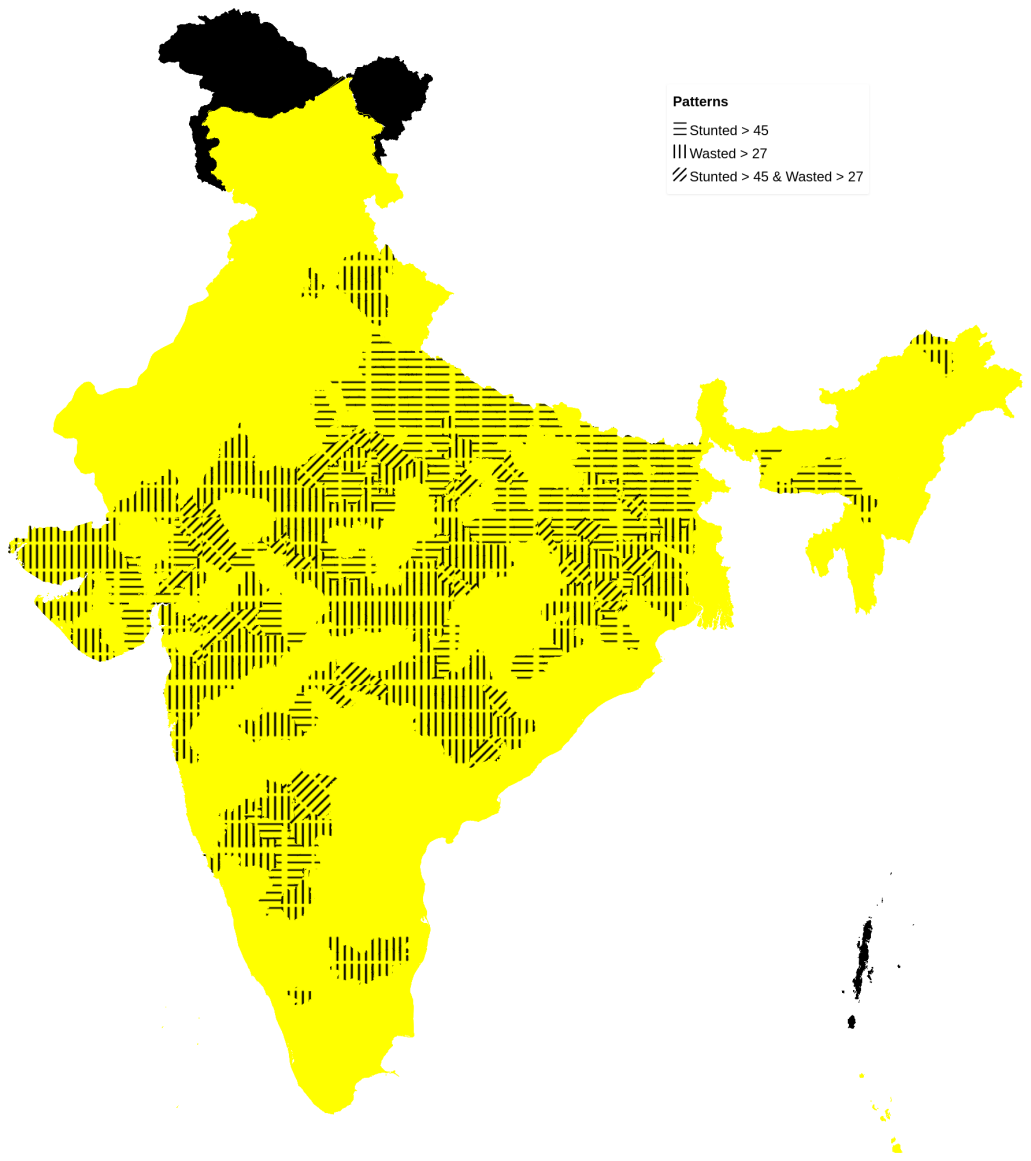
Map of India showing areas with higher prevalence of stunting (>45%) in horizontal bars and those with higher prevalence of wasting (>27%) in vertical bars.

Districts with high prevalence of stunting (ranging from 46–65% district prevalence) numbered 108 with higher representation from the poorer states (number of districts followed by percentage given in parenthesis) of Uttar Pradesh (30; 28%) Bihar (28; 26%) and Madhya Pradesh (22; 20%). Districts with higher wasting prevalence (ranging from 28–47% district prevalence) were also 108 in number with predominantly tribal districts of Jharkhand (14; 13%), Madhya Pradesh (19; 18%), Maharashtra (12; 11%), Rajasthan (10; 9%) and Gujarat (10; 9%) having higher representation. High stunting is more concentrated in north and eastern India, whereas high wasting areas are located primarily in central India, along with those districts having both stunting and wasting ( Figure 1). There are only 18 districts with both stunting and wasting. Out of them 13 have millets or maize as either the highest (8) or second highest crops (5) ( Table 1). Of these 18 districts, there are three from Rajasthan, which have more maize cultivation than any other cereal crop (Udaipur 62 %, Banswara 51%, and Dungarpur 47%). In these districts, it is likely that maize consumption (without nixtamalization; see discussion) as a staple among the poor could be contributing to both stunting and wasting. Similarly, Arwal and Sheikpura districts in Bihar, and Pashchimi Singhbhum from Jharkhand are predominantly rice and wheat growing. In these districts, assuming rice and wheat staple, the pattern of malnutrition could be stunting followed by wasting.

**Table 1.** Districts with high prevalence of stunting and high prevalence of wasting as per the integrated dataset from NFHS4 and agriculture statistics ^43^.

No	District	State	Major crop1	Percentage	Major crop2	Percentage	Major crop3	percentage
1	Arwal	Bihar	Rice	73.64	Wheat	25.21	Maize	0.9
2	Sheikhpura	Bihar	Wheat	49.37	Rice	49.12	Maize	1.5
3	Kaushambi	Uttar Pradesh	Wheat	53.69	Rice	35.95	Bajra	6.33
4	Chitrakoot	Uttar Pradesh	Wheat	62.43	Bajra	13.36	Rice	11.23
5	Sheopur	Madhya Pradesh	Wheat	66.24	Rice	21.57	Bajra	10.58
6	Morena	Madhya Pradesh	Wheat	50.46	Bajra	48.11	Rice	0.44
7	Alirajpur	Madhya Pradesh	Maize	42.4	Wheat	21.99	Bajra	14.42
8	Barwani	Madhya Pradesh	Maize	35.02	Wheat	33.77	Jowar	26.04
9	Bhind	Madhya Pradesh	Wheat	67.57	Bajra	22.00	Jowar	4.06
10	Udaipur	Rajasthan	Maize	62.65	wheat	29.25	Jowar	2.62
11	Banswara	Rajasthan	Maize	51.63	Wheat	34.42	Rice	12.17
12	Dungarpur	Rajasthan	Maize	47.50	Wheat	35.06	Rice	13.19
13	Yadgir	Karnataka	Rice	62.26	jowar	24.99	Bajra	12.35
14	Gulbarga	Karnataka	Jowar	86.11	Bajra	5.10	wheat	3.89
15	Chatra	Jharkhand	Rice	78.44	Maize	11.7	Wheat	8.99
16	Paschimi Singhbhum	Jharkhand	Rice	98.89	Wheat	0.61	Maize	0.47
17	Yavatmal	Maharashtra	Jowar	83.63	Wheat	10.55	Bajra	3.36
18	Nandurbar	Maharashtra	Jowar	42.29	Rice	14.87	Maize	12.62

**Figure 2.**
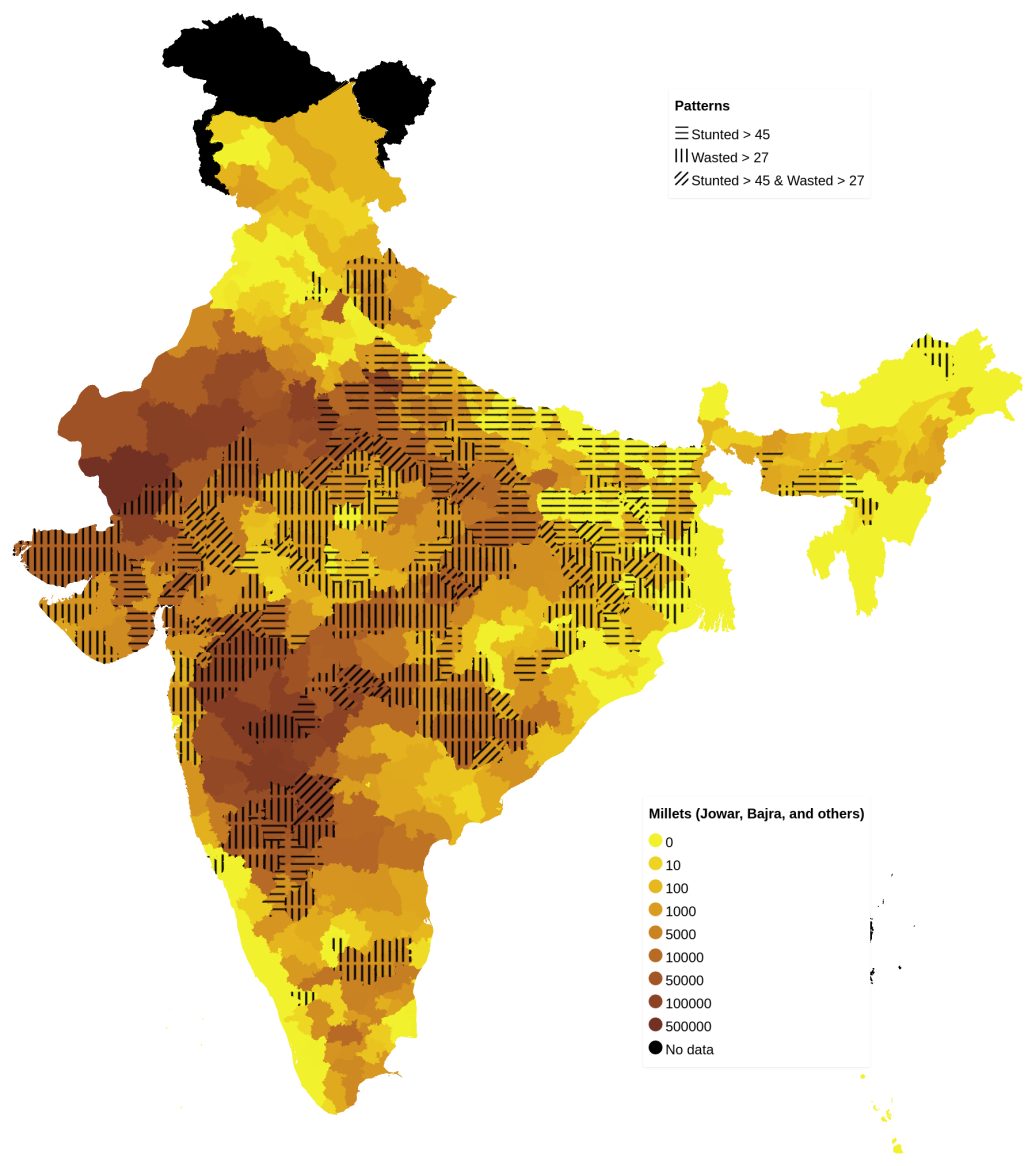
Map of India showing overlap between high prevalence of stunting, high prevalence of wasting with DSCQ of Jowar, Bajra and other millets by district.

On examining the district-level patterns of subsistence cultivation of millets by district (based on DSCQ of Jowar, Bajra and other millets) overlaid over districts having higher prevalence of stunting and wasting, we find that there is an overlap of districts with wasting and those with stunting and wasting with higher DSCQ for millets ( Figure 2). However, large areas with higher DSCQ particularly in North West and some parts of central India do not show either stunting or wasting. Similar maps, separately showing overlap of high stunting and high wasting with per-capita cultivation of jowar, wheat, rice, bajra and other millets are also available ^51^. There is an overlap of districts with high wheat and rice cultivation in the well irrigated Gangetic plain (North and Eastern parts) with stunting. Cultivation of other millets is scattered throughout the country with an overlap with high prevalence of wasting. The large irrigated areas in the Northwest & Central India with high DSCQ of Bajra & Jowar also have higher DSCQ of rice and wheat as seen from the maps (irrigated areas having cultivation of rice, wheat along with Bajra & Jowar). Maize cultivation is all over the country with no clear overlap with either stunting or wasting.

**Figure 3.**
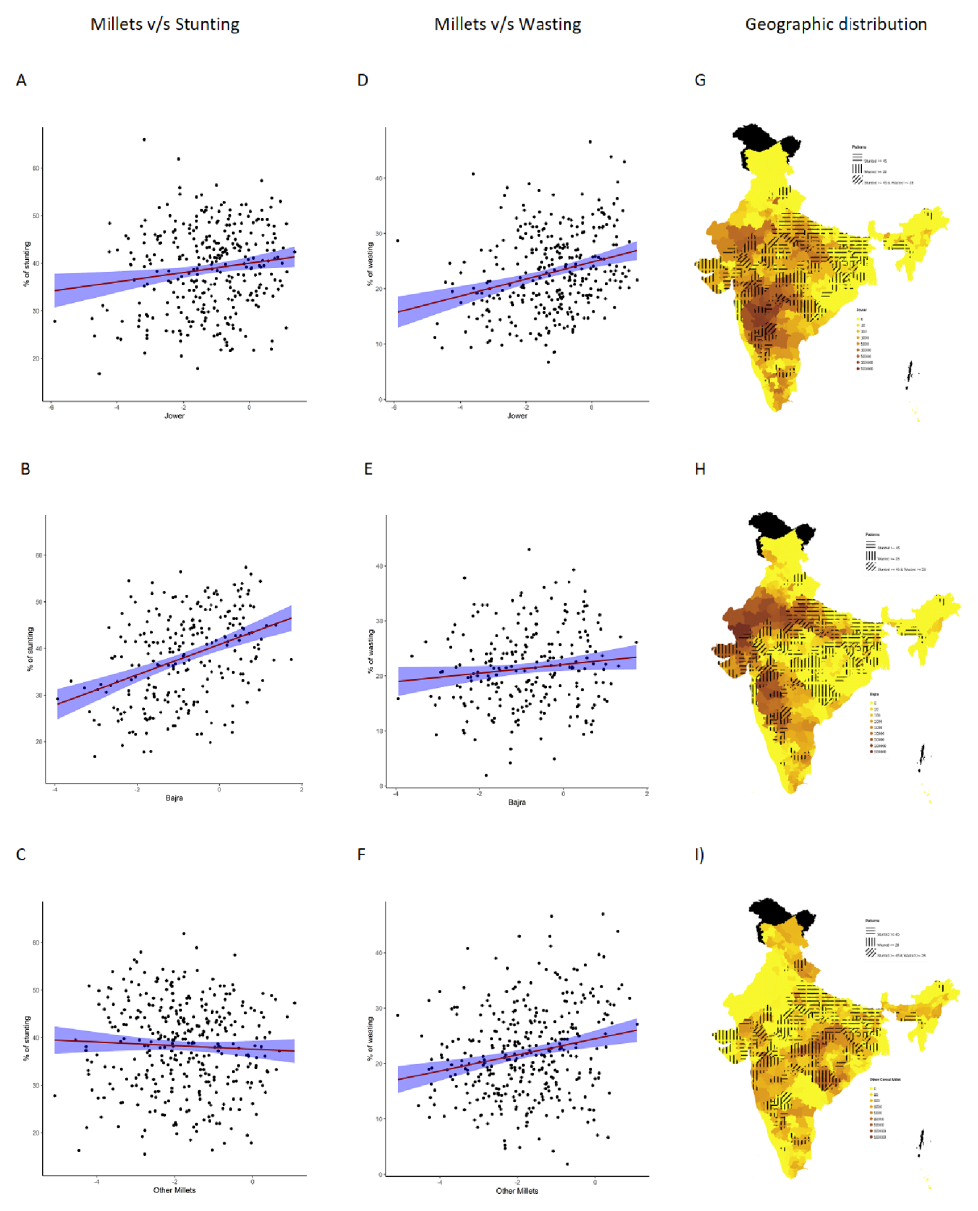
Plots examining relationship between jowar, bajra and other millets cultivation with stunting and wasting at district level along with map showing the overlaps for each type of millet with stunting and wasting. ( **A**) Scatterplot of stunting v/s district subsistence cultivation quantum of jowar by poor, ( **B**) Scatterplot of stunting v/s district subsistence cultivation quantum of bajra by poor, ( **C**) Scatterplot of stunting v/s district subsistence cultivation quantum of other millets by poor, ( **D**) Scatterplot of wasting v/s district subsistence cultivation quantum of jowar by poor, ( **E**) Scatterplot of wasting v/s district subsistence cultivation quantum of bajra by poor, ( **F**) Scatterplot of wasting v/s district subsistence cultivation quantum of other millets by poor, ( **G**) Geographic distribution of district subsistence cultivation quantum of jowar by poor, stunting >45 & wasting >28, ( **H**) Geographic distribution of district subsistence cultivation quantum of bajra by poor, stunting >45 & wasting >28, ( **I**) Geographic distribution of district subsistence cultivation quantum of other Millets by poor, stunting >45 & wasting >28; (relationship examined for each cereal type separately in extended data ^51^)

Overall, increase in cultivation of jowar, bajra and other millets is independently associated with increase in prevalence of both stunting and wasting (see Figure 3). When the association was examined for individual millets, whereas jowar cultivation did show an association with increase in both stunting and wasting, increase in bajra cultivation was associated only with increase in stunting. Increase in cultivation of other millets was associated with increase in wasting only (a reverse trend was seen with stunting). As expected, there was either no change or decrease seen when we examined association between increase in rice or wheat cultivation with stunting and wasting (except for the increase in stunting associated with increase in wheat cultivation).

**Figure 4.**
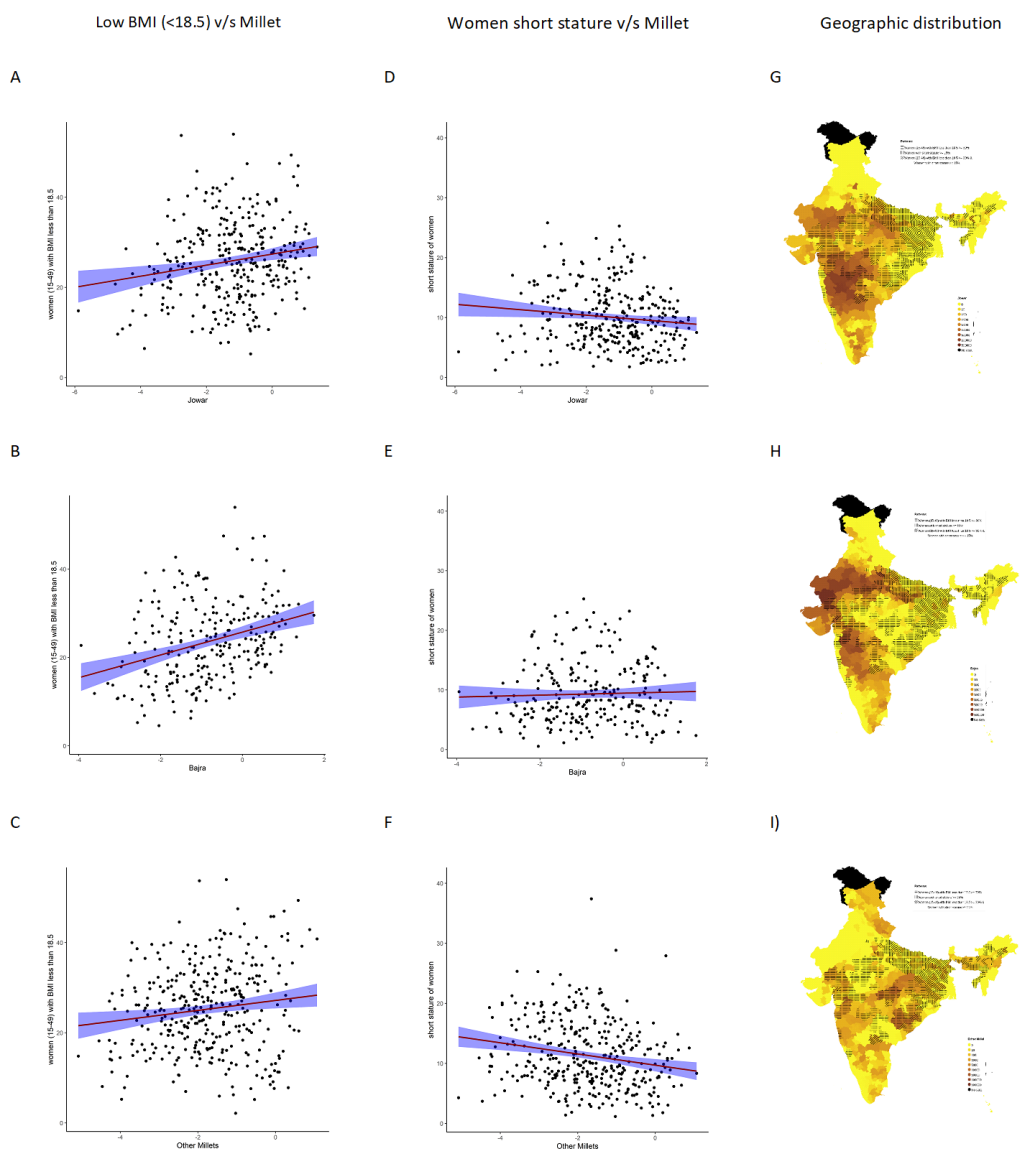
Plots examining relationship between type of millet cultivated with low BMI and short stature in women (15–49) at district level along with map showing the overlaps for each type of millet with low BMI and short stature in women (15–49). ( **A**) Scatterplot of low women BMI v/s district subsistence cultivation quantum of jowar by poor, ( **B**) Scatterplot of low women BMI v/s district subsistence cultivation quantum of bajra by poor, ( **C**) Scatterplot of low women BMI v/s district subsistence cultivation quantum of other millets by poor, ( **D**) Scatterplot of women short stature v/s district subsistence cultivation quantum of jowar by poor, ( **E**) Scatter plot of women short stature v/s district subsistence cultivation quantum of bajra by poor, ( **F**) Scatterplot of women short stature v/s district subsistence cultivation quantum of other millets by poor, ( **G**) Geographic distribution of district subsistence cultivation quantum of jowar by poor low women BMI and Women short stature, ( **H**) Geographic distribution of district subsistence cultivation quantum of bajra by poor low women BMI and Women short stature, ( **I**) Geographic distribution of district subsistence cultivation quantum of other millets by poor low women BMI and women short stature; relationship examined for each cereal type available in extended data ^52^.

On examining the relationship between low BMI and short stature with millet cultivation, we see an association between increase in jowar, bajra and other millets (both when examined individually and altogether) with prevalence of low maternal BMI. For short stature, the relationship with millets was either reversed or there was no relationship ( Figure 4).

We examined the possible role of maternal malnutrition in districts with high millet cultivation.

We used the data available in NFHS4 on prevalence of low BMI and short stature of women in 15–49 years age group as a proxy for maternal BMI and short stature. On examining the high prevalence of low BMI (≥30%; BMI less than 18.5) in women of 15–49 years of age and short stature (≥15% with stature less than 145 cm) in women 15–49 years of age, we found that the areas with higher short stature were distributed in the Northern Gangetic plains and Northeast India, whereas the areas with higher prevalence of low BMI were located pre-dominantly in peninsular India. The areas with low BMI overlap over millet-growing areas ( Figure 4) in a pattern similar to the prevalence of wasting in children seen earlier ( Figure 2).

**Figure 5.**
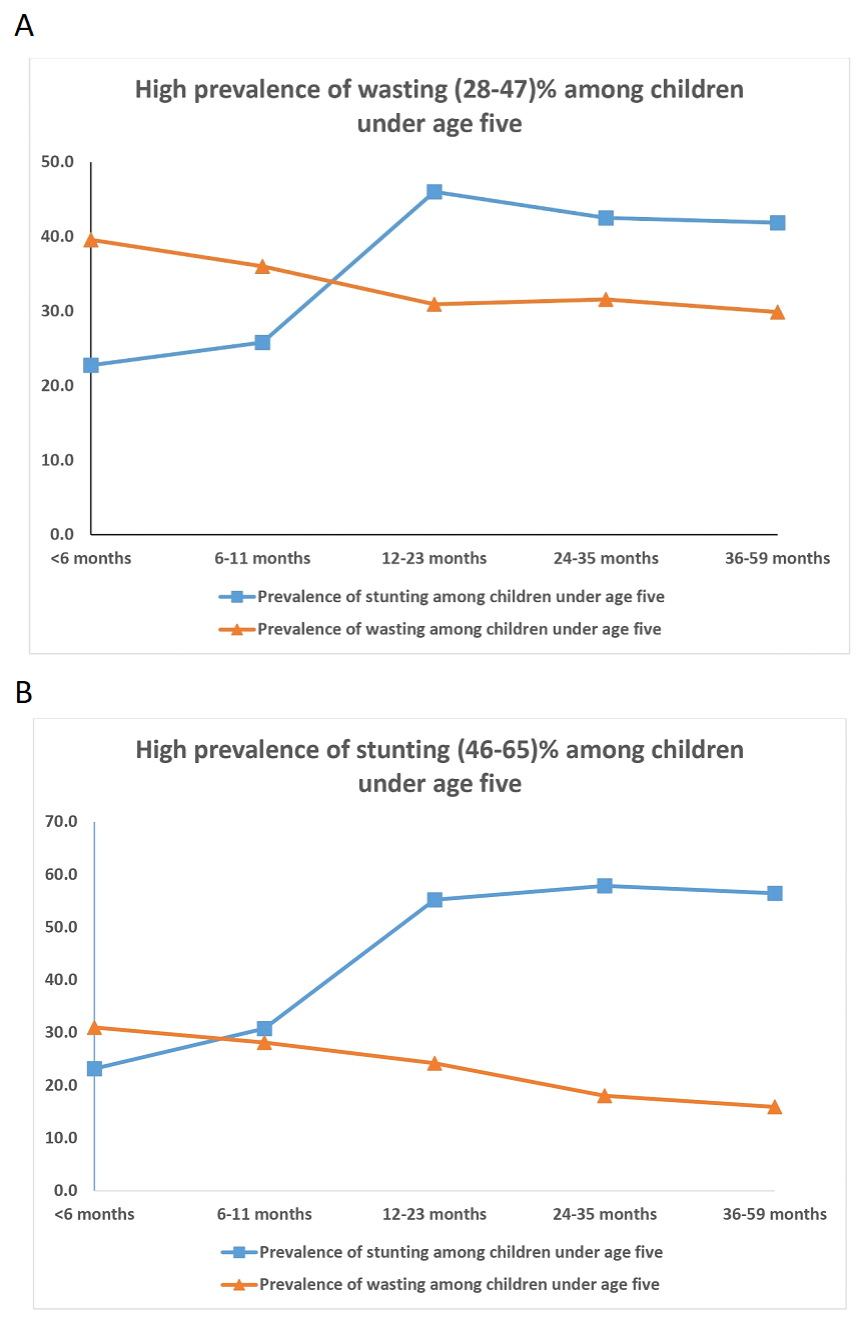
( **a**) Age profile of children in high wasting (28–47%) districts; ( **b**) Age profile of children in high stunting (45–67%) districts.

In districts with high stunting and wasting, wasting showed an early onset with highest wasting (40%) at 6 months of age ( Figure 5). The age-distribution of stunting was similar for both groups of districts. The districts with high prevalence of stunting had highest age-specific stunting prevalence at 12 months with a plateau thereafter till five years of age. The earlier onset of wasting suggests maternal nutritional factors affecting intra-uterine growth during pregnancy and possibly continuing in early infancy while being breast fed.

**Figure 6.**
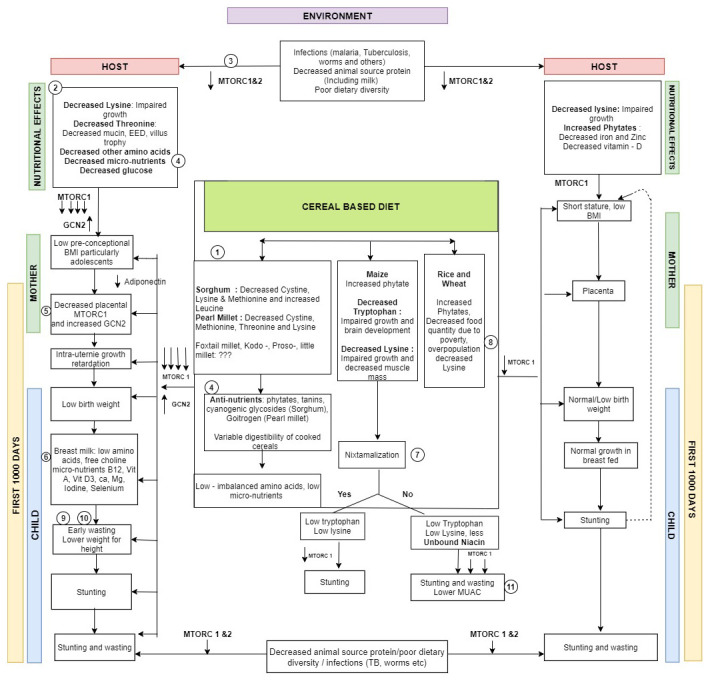
A schematic figure showing the possible pathways linking staple cereal consumption with child malnutrition with key steps highlighted (with references to evidence from literature).

Clearly, there are distinct geographic patterns of both stunting and wasting as well as low BMI and short stature of women of 15–49 years age group. There is also similarity in patterns of association between low maternal BMI with child wasting and maternal short stature with child stunting. Taken from the prism of nutrient intake in a largely cereal based diet, the cultivation of other cereal crops in the same area could also be altering maternal and child nutrition prevalence indices through dietary diversity (as is possibly the case with bajra grown in areas with rice and wheat cultivation seen in northern India), whereas large-scale subsistence cultivation of millets in dry/semi-arid areas of central India are more likely to be associated with higher wasting. Other factors like quantity of food, intake of other proteins through milk and other animal sources during and before the first 1000 days by the mother, the dietary matrix and diversity and presence of infections are also important. However, the ecogeographic pattern malnutrition can be explained to some extent by staple cereal cultivation. Hence, an attempt has been made to build a hypothetical framework explaining the plausible pathways through which millet staple based cereal based diet could produce wasting ( Figure 6)

The millet based diet has putative deficiencies in amino acids and micronutrients ( Figure 6 pathway 1) with variable digestibility after cooking ( Figure 6 pathway 2) due to the presence of antinutrients ( Figure 6 pathway 4) ^36, 47, 53– 55^. This leads to a greater lowering of amino acids and/or micronutrients like niacin which in turn leads to a substantial lowering of MTORC1 and rise in GCN2 contributing to wasting ( Figure 6 pathway 5) ^56– 58^. The lowering of MTORC1 & GCN2 at the cellular level causes low BMI in women ( particularly in growing adolescents) with consequent decreased acquisition of nutrients by placental syncytiotrophoblast ^59– 62^. This in turn leads to intra-uterine growth retardation and low birth weight. These nutritional effects on the malnourished mother persist during breast feeding ^15^ ( Figure 6 pathway 6) leading to early wasting and lower weight for height seen in regions with higher wasting ( Figure 6 pathway 9) ^63– 67^. Maize consumption without nixtamalization could lead to lesser unbound niacin ( Figure 6 pathway 7) ^68– 70^ and both stunting and wasting with lower MUAC ( Figure 6 pathway 11) ^63, 67^. Poverty in highly populous areas growing rice & wheat could be contributory to stunting ( Figure 6 pathway 8) ^12, 19, 39^. Lower lysine, high phytates with lowered micronutrients like zinc and iron in cereal based diet in wheat and rice growing areas with poor dietary diversity could lead to stunting, through decreased MTORC1 stimulation ( Figure 6 pathway 10) ^56, 59, 71– 74^. Infections in the growing child can decrease stimulation of both MTORC1 and 2 through T cell mediated mechanisms ( Figure 6 pathway 3) ^75^.

## Discussion

Poverty and its antecedents affecting dietary intake, healthcare and poor environmental conditions can broadly explain malnutrition prevalence, particularly stunting worldwide ^76^. Nearly three decades ago, Victora identified the several-fold variation in wasting prevalence in areas with similar stunting prevalence ^77^. Child malnutrition is more common in rural non-pastoral communities pointing to the protective role of milk proteins in the mother-child dyad during the first 1000 days; indeed breastfeeding has been linked with intelligence, educational attainment and income security in adult life ^23, 24, 78– 80^. In India too, the age-profiles of children in districts with predominant stunting differs from those with higher prevalence of wasting in line with worldwide differences in such patterns ^64^. In a prescient paper, Martorell *et al.* highlighted higher levels of early wasting with similar levels of stunting in India, in comparison to Gautemala ^65^. They have linked the higher prevalence of low BMI and maternal anaemia in India, while suggesting that improvements in maternal nutrition through prenatal interventions and during breastfeeding could address this problem of early wasting. Apart from this comparative analysis, the current list of possible explanations for the particular patterns of wasting in India include several macro-level and general determinants of malnutrition such as poor status of women ^12^, the *thin-fat* infant phenotype ^81^, chronic dietary insufficiency, poor dietary quality, marked seasonality, and poor levels of sanitation ^2, 82^. Our analysis shows that ultimately, disaggregation of the data on type of malnutrition prevalence within India at a finer-scale along with proximate dietary factors probably holds the clues to such differences.

### Global patterns of wasting linked to millet subsistence

The explanations for ecogeographic patterns of wasting in India appear to at least partially rest in subsistence cultivation patterns and staple consumption of millets as seen in our results. This pattern can be reproduced globally. Subsistence farming of millets is prevalent in other areas with some of the highest prevalence of wasting worldwide such as Yemen and Sub-Saharan Africa ^1^. Similarly, in the low-lying areas of Jizan province of Saudi Arabia (which is adjoining Yemen), pearl millet is cultivated and widely used as a staple; expectedly, wasting prevalence in Jizan is the highest among all provinces in Saudi Arabia ^83– 85^. Malnutrition in early infancy has been shown in a recent paper to be highly prevalent in India, Niger, Nigeria, Burkina Faso and Mali ^86^. These are in fact the top countries that produce millets for human consumption through subsistence farming ^87^. These apparent differences in type of malnutrition linked to millets has also been reproduced in observational studies. In a comparison of Bwamanda district (Democratic Republic of Congo with maize and Cassava staple) and Niakhar region (in Senegal with staple millet consumption), the former had higher proportion of wasting measured by lower MUAC, while the latter had earlier onset of wasting and higher prevalence of low weight for heights ^63^. On examining FAOSTAT data ^87^ to explain Grellety and Golden’s assessment of differences in types of malnutrition across various countries, we found higher prevalence of wasting by MUAC to be a feature of maize-cultivating countries, while the countries reporting low weight for height are typically cultivating millets ^67, 69, 87^. At a global level, the top 50 countries ranked among the hidden hunger scores ^88^ had either maize or millets/sorghum as staple among the top two cereals (as seen from FAOSTAT data of the top two cereals produced) ^69, 87^.

### Millet protein quality

In rural India, the chief source of proteins are cereals ^89^ Further, there is a socio-economic gradient to protein quality; tribal populations and the poor consume lesser lysine-containing proteins primarily through cereals ^90, 91^. The diet of rural pregnant and lactating women is particularly inadequate with respect to quality of protein, thereby contributing to early malnutrition. Lysine content of millet (22 mg/g of protein) and sorghum (24 mg/g of protein) is the least among cereals in comparison to rice (35 mg/g of protein) and wheat (27 mg/g of protein) ^89^. The proportionate amino-acid requirement at infancy is the highest and shows an age-related decline ^92– 94^. Extensive dependence on millets and sorghum as a staple diet among millions of poor rural communities where subsistence farming is the mainstay, in semi-arid and rainfed agricultural landscapes, motivated the FAO to commission a detailed assessment of their dietary protein quality ^47^. The report unequivocally highlights the inadequacy of millet and sorghum proteins for infants and young children based on amino acid scores ^47^. The other inexpensive and subsistence crop in India is maize with only 20% consumption, which (apart from its association with Kwashiorkor in the initial description by Cecily Williams) too has been extensively investigated for its causation of Pellagra ^55, 95, 96^. Similarly, Sorghum has been shown to be associated with Pellagra in Indian studies ^57^.

Sorghum, millets and maize share a common evolutionary ancestor in the grass family (Family *Poaceae*, sub-family *Panicoideae*), and can grow in arid/semi-arid agro-climatic regions where other crops often do not produce optimal yields through their dependence on the C4 carbon fixation pathway of photosynthesis ^98– 101^. Millets are also usually not traded in markets but consumed directly by poor subsistence farmers ^47^. Sorghum, which is cultivated, can be the white (low tannin) and red (high tannin) variety ^55^. Sorghum protein is stored in Kafirins and is deficient in amino acids lysine, arginine, tryptophan and threonine with an excess of leucine ^47, 54, 55, 57^. The deficient micronutrients are selenium, vitamins A, B12, and E, as well as folic acid. Miillets in general have higher tannins ^22, 47^; pearl millet has antinutrients like phytic acid, goitrogenic polyphenols, and tannins ^53^. Despite its better amino-acid profile (among the millets) the digestibility of proteins is probably less than other major grains due to antinutrients ^53, 101^. In India, the commonest mode of consumption of millets is by milling followed by removal of bran and making unleavened bread using (typically) dry heat ^102^. Porridge-like preparations and cooked grains are also common. These modes are inferior to processes such as fermentation, germination or in combination which increase the availability of micronutrients, such as iron and zinc ^22, 71^. Such cultural and social norms are important determinants of bioavailability of micronutrients. For instance, maize is consumed in Latin America after nixtamalization, unlike in India where it is consumed directly, possibly explaining the stunting and wasting in the few districts in India where maize-growing for staple consumption is high ^70^.

The lack of dietary diversity as a contributor to malnutrition is well known. Both protein (quantity and in terms of all essential amino-acids) and micronutrient deficiencies contribute to malnutrition ^72, 79, 92, 103, 104^. Over the last three decades, pioneering research on cell growth has helped elucidate the critical role of complex intracellular nutrient-sensing mechanisms and their linkages with upstream and downstream pathways incorporating endocrine inputs for growth, primarily centred on the role of protein kinases, MTORC 1 and MTORC2 ^59, 62, 105– 107^. Another protein kinase, GCN2 acts in concert with MTORC1 in sensing amino acid deficiencies ^108^. Millets are indeed gluten-free, have high fibre and antioxidant content and have recently seen a spike in use ^109– 111^. However, their use among poor communities with limited dietary diversity and consumption as a staple cereal by pregnant and nursing mothers and children in these communities during the first 1000 days of life could be associated with malnutrition.

The focus on protein quality has important biomedical and policy implications. Recent success with the use of peanut paste with milk based ready-to-use therapeutic food (RUTF) is being supplemented with well-intentioned attempts to use locally available ingredients in community-based malnutrition management approaches ^103^. These soya-maize-sorghum (SMS) formulations have been reported to be inferior in trials, particularly in children less than two years of age *vis-a-vis* peanut-based RUTF ^6, 112, 113^. However, when supplemented with free amino acids (free aminoacid soya-maize-sorghum RUTF), it has been shown to be as efficacious as standard peanut milk based RUTF ^6^ thereby highlighting amino acids to be the missing link in the millet-based RUTF in the first 1000 days of life.

### Study limitations

An important limitation of our analysis is the limited data on low birth weight and foodgrain consumption at finer scales, which would have allowed for confirmation of our hypothesis at household level. One of the reasons for this is that the NFHS surveys record cereal consumption without paying attention to type of cereal. Indeed, our analysis shows that this is an important change in NFHS and demographic health surveys worldwide that may be needed to gain better understanding of pathways to malnutrition. The use of millet cultivation as a proxy for consumption too is a source of noise in our data, as some of the cultivation is likely to be for non-human use (primarily fodder). The data on availability of nutrients from millet consumption, as per current nutritional assays (stable isotope-based), is meagre, to the best of our knowledge. Such data from cereal consumption could help in linking the dietary matrix to effects.

## Conclusion

Wasting prevalence among children in India has an ecogeographic pattern. It appears to be linked to millet consumption as a staple. In poor households that consume millets, low birth weight, early onset of malnutrition in children (in infants under six months) and wasting are probably linked to low BMI in mothers which in turn is linked to dependence on millets. In these mothers and infants, the wasting is possibly driven through greater lack of MTORC 1 signalling (in comparison to stunting) and higher GCN2 signalling both *in utero* and after birth, mediated by multiple deficiencies of amino-acids and other nutrients. Areas with large population of people using millets as staple will require special interventions to address early origin of wasting. MUAC and type of cereal consumed should be incorporated in the anthropometry measures surveyed in NFHS4 and global demographic and health surveys to enable better assessment of patterns of malnutrition. State of the art research in nutrient sensing should be integrated with agriculture , food science, delivery systems and dietary matrix for translational benefits to accrue to the wider population.

## Data availability

### Underlying data

Figshare: Dataset used to assess relationship between millet cultivation and malnutrition patterns in India at district level. https://doi.org/10.6084/m9.figshare.12236789.v2 ^43^

This project contains the following underlying data:

- malnutrition_dataset_for_publication.xlsx (Dataset used for analysis described in the papert)
- Malnutrition and millets – India – DACNET NFHS 4.docx (Word document explaining how the dataset was prepared)

### Extended data

Figshare: Plots examining relationship between type of millet cultivated with stunting and wasting at district level along with map showing the overlaps for each type of millet with stunting and wasting. https://doi.org/10.6084/m9.figshare.12206135.v1 ^51^

This project contains the following extended data:

- malnutrition_millets and malnutrition.pdf (PDF file with panel of seven plots and maps, each showing relationship between type of millet cultivated with stunting and wasting at district level and the corresponding map showing the overlaps of each type of millet with stunting and wasting)

Figshare: Plots examining relationship between low BMI and short stature in women 15–49 with stunting and wasting at district level along with map showing the overlaps for each type of millet with low BMI and short stature in women (15–49). https://doi.org/10.6084/m9.figshare.12206264.v2 ^52^

This project contains the following extended data:

- malnutrition_bmi_short_status.pdf (PDF file with panel of seven plots and maps, wach showing relationship between low BMI and short stature in women 15–49 with stunting and wasting at district level alogn with maps showing overlaps for each type of millet with low BMI and short stature in women (15–49))

Data are available under the terms of the Creative Commons Attribution 4.0 International license (CC-BY 4.0).

## Software availability

Source code available from: https://gitlab.com/asdofindia/malnutrition-crops-maps

Archived source code at time of publication: http://doi.org/10.5281/zenodo.3828725 ^50^

License: MIT license
