# Eco-geographic patterns of child malnutrition in India and its association with cereal cultivation: An analysis using demographic health survey and agriculture datasets

**DOI:** 10.12688/wellcomeopenres.15934.3

**Published:** 2021-09-30

**Authors:** Rama Krishna Sanjeev, Prashanth Nuggehalli Srinivas, Bindu Krishnan, Yogish Channa Basappa, Akshay S. Dinesh, Sabu K. Ulahannan

**Affiliations:** 1 Pediatrics, Rural Medical College, Pravara Institute of Medical Sciences, Loni (BK), Ahmednagar district, Maharashtra, 413736, India; 2 Health equity cluster, Institute of Public Health Bengaluru, Bengaluru, Karnataka, 560070, India; 3 Physiology, Rural Medical College, Pravara Institute of Medical Sciences, Loni (BK), Ahmednagar district, Maharashtra, 413736, India; 4 Metastring Foundation, Bengaluru, Karantaka, 560020, India

**Keywords:** Millets, malnutrition, wasting, stunting, MTORC1 (mechanistic target of Rapamycin complex1), GCN2 (general control non derepressible 2), DSCQ (District subsistence cultivation quantum)

## Abstract

**Background: **High prevalence of maternal malnutrition, low birth-weight and child malnutrition in India contribute substantially to the global malnutrition burden. Rural India has disproportionately higher levels of child malnutrition. Stunting and wasting are the primary determinants of child malnutrition and their district-level distribution shows clustering in different geographies and regions. Cereals, particularly millets, constitute the bulk of protein intake among the poor, especially in rural areas in India where high prevalence of wasting persists.

**Methods: **The last round of National Family Health Survey (NFHS4) has disaggregated data by district, enabling a more fine-scale characterisation of the prevalence of markers of malnutrition. We used data from NFHS4 and agricultural statistics datasets to analyse relationship of prevalence of malnutrition at the district level and area under cereal cultivation. We analysed malnutrition through data on under-5 stunting and wasting by district.

**Results: **Stunting and wasting patterns across districts show a distinct geographical and age distribution; districts with higher wasting showed relatively higher prevalence before six months of age. Wasting prevalence at district level was associated with higher cultivation of millets, with a stronger association seen for jowar and other millets (Kodo millet, little millet, proso millet, barnyard millet and foxtail millet). District level stunting was associated with higher district level cultivation of all crops (except other millets). The analysis was limited by lack of fine-scale data on prevalence of low birth-weight and type of cereal consumed.

**Conclusions: **Better cereal cultivation and consumption data will be needed to confirm causal pathways contributing to potential ecogeographic patterns. The cultivation of other millets has a strong association with prevalence of wasting. State-of-the-art studies that improve our understanding of bio-availability of amino acids and other nutrients from the prevalent dietary matrices of rural poor communities will be needed to confirm causal pathways contributing to potential eco-geographic patterns.

## Introduction

Undernutrition in childhood includes intrauterine growth restriction (defined as below the 10 ^th^ centile of intergrowth standards for the gestational age and sex), apart from stunting (z score for length/height for age less than 5 years below -2), wasting (z score for weight for length /height for age less than 5 years below -2) and underweight (z score for weight for age below -2) ^1^. Low pre-pregnancy BMI, low maternal BMI (< 18.5 kg/m2) ,maternal short stature and maternal micronutrient deficiency or anemia all contribute to small for gestational age, low birth weight and prematurity ^2^. Out of the estimated 20.5 million babies born low birth weight, 48% are born in South Asia. India alone is estimated to have 100 million adult women with low BMI ^1, 2^. Globally, the World Health Organization (WHO) estimates that among children under five, about 151 million suffer from stunting and 51 million from wasting with consequent risks of mortality, morbidity and delayed development ^3^. The latest stunting trends indicate increases in Africa along with substantial reductions across Asia. However, with regards to wasting, with a regional prevalence of 12%, South Asia accounts for half of all wasted children globally ^1, 4, 5^. India reports 15.4% wasting of children under 5 years numbering about 27 million ^4^. South Asia is also estimated to have ~45% of the global burden of stunting. The socio-economic gains and poverty reduction of the past decades have not translated into commensurate reduction of stunting and wasting in children, often characterised as, *the Asian enigma* ^6– 8^. 

### Subsistence farming and millet dependence

Indian states consist of 640 districts (at the time of NFHS4) with wide differences in geography, climate and the main agricultural crops. India has a large and poor rural population (68.9% rural with 25.5 % rural poverty prevalence), and over half (54%) of the working rural population (481.9 million) are cultivators and agricultural labourers ^9, 10^. Small land-holding farmers (owning less than two hectares of land) and their families constitute more than half the country’s population. Only half (96.46 million hectares) of the total area under cultivation (198.36 million hectares) is irrigated ^11^. Although, rice and wheat together constitute 75% of total area under food grain cultivation, Jowar (Sorghum) and Bajra (pearl millet) make up a significant 13.8%. However, the distribution of food grain cultivation in irrigated land varies, with rice (60%) and wheat (94.2%), expectedly being grown largely on irrigated land. In contrast, Sorghum (Jowar) and Pearl millet (Bajra) are grown largely in non-irrigated lands, most likely by small land-holding farmers in monsoon-dependent arid or semi-arid regions of the country, which are also among the poorest ^12, 13^. Cereal cultivation and consequently household food grain consumption and diets in such regions are likely driven by these strong linkages between agro-climatic, edaphic, and eco-geographic factors, more so among poorer households with socio-economic barriers to achieve dietary diversity.

A study based on National Family Health Survey-3, which reported results at the state level for India in 2005–6, demonstrated considerable geographic variation among the states of India with regards to child malnutrition, with higher levels of stunting seen in Uttar Pradesh, Uttaranchal & Gujarat ^14^. In contrast, higher wasting levels were seen in Madhya Pradesh, a state in central India. A nutritional survey among preschool children in three tribal regions belonging to different ecological zones in the state of Madhya Pradesh, India, namely Jhabua, Bastar and Sarguja, showed greater extent and severity of malnutrition among children in Jhabua. The staple cereals reported in the study for Jhabua was maize and Sorghum, while for Bastar and Sarguja, it was rice ^15^. Sorghum, as staple, has also been linked to endemic pellagra among farm workers in Hyderabad by Gopalan ^16^. 

The Lancet 2008 series too has brought out this aforementioned pattern of child malnutrition, with areas having similar prevalence of stunting demonstrating substantial differences in wasting ^17^. Likewise, low women’s BMI (15–49 years age) too, has numerous geographical subnational hotspots in South Asia ^1^. 

Geo-spatial heterogeneity in prevalence of child malnutrition across Indian districts has been reported ^18^. The National Family Health Survey 4 (NFHS 4) was published in 2015 with district-level data for the first time ^19^. Based on unpublished field observations of wasting prevalence among populations depending on millet as staple in rural Maharashtra (spanning western and central India), we critically examined the spatial patterns of prevalence of stunting and wasting at the district level across India with the objective of exploring the role of dietary staple cereal consumption pattern using cultivation pattern as a Proxy. Ragi (finger millet; Eleusine coracana) was excluded because it belongs to a distinct sub-family in the grass family Poaceae and has a relatively better nutritional profile ^20– 22^. We also propose a hypothetical pathway that integrates evidence emerging from agro-climatic and geographic patterns with physiological mechanisms of malnutrition.

## Methods

We analysed district-level secondary data on under-5 stunting and wasting as reported in NFHS4 with district-wise crop cultivation data to assess geo-spatial overlaps and risk relationships between pre-school child malnutrition and cultivation of staple cereal crops. NFHS is a standardised and periodic nationally representative survey. NFHS4 covered 601,509 households, 699,686 women aged 15–49 years and 103,525 men aged 15–54 years that provides comprehensive data on various aspects of maternal and child health ^18, 23^. NFHS-4 provides unit level data (for each of the 640 districts of India at the time of survey) for download upon request via the demographic health survey data repository ^23, 24^. We extracted data on population of each district from the 2011 Census ^10^. We included other socio-demographic variables with known associations with malnutrition from NFHS4 to assess their relative contribution to childhood wasting and stunting, at district level using multivariable linear regression.

### Definitions and data sources

We considered the following cereal crops widely grown and reported in Indian agriculture databases and the Directorate of Millets Development (under Department of Agriculture, Co-operation and Farmers Welfare) in our analysis: rice( *Oryza sativa*), wheat( *Triticum aestivum*) , maize( *Zea mays*), jowar (sorghum; *Sorghum bicolor*), bajra (pearl millet; *Pennisetum glaucum*) and other millets (kodo millet: *Paspalum scrobiculatum*, little millet: *Panicum sumatrense*, proso millet: *P. miliaceum*, barnyard millet: *Echinochloa esculenta* and foxtail millet: *Setaria italica*). Henceforth, millets in the text means Jowar, Bajra and other millets combined.

We adopted the definitions of districts with high prevalence of wasting and stunting from district-level malnutrition analysis by Junaid and Mohanty ^18^, which has considered >46% district-level stunting prevalence (Z score **≤** 2), and >28% district-level wasting prevalence (Z score **≤** 2) as representing high prevalence districts for stunting and wasting respectively.

We extracted variables of interest from NFHS4 (see variables listed below). For data on cultivation of cereal crops, we used DACNET, a web-based land use statistics information system maintained by the Agriculture Informatics Division of the National Informatics Centre of the Government of India ^25^. 

The following data were extracted to prepare a district-level dataset for analysis ^26^:

1. From the 2011 census data, district-wise total and rural population
2. From the NFHS4 data,
  a. using appropriate weights BMI less than 18.5 and short stature less than 145cm of women aged 15–49 years, utilization of Anganwadi, dietary diversity (age 6–23months), women with 10years or more of education, household wealth quintiles(lowest and second), open defecation and rural population.
  b. district-level percentage of wasting and stunting was calculated from the children dataset
  c. percentage of people in household wealth quintiles, open defecation for a given district was calculated from household dataset
3. Various crop data is available in state-wise reports compiled by the Ministry of Agriculture and Farmers Welfare. We extracted district-level area under cultivation of cereals: rice, wheat, maize, ragi, bajra, jowar, and and millets (by type as defined above) into a spreadsheet. Data was from the latest state-wise reports available at the time of analysis at DACNET ^25 ^(data for most states ranged for years between 2014–17 except Maharashtra 2002–03, Manipur 2004–05 and Gujarat 2007–08; all data in hectares converted to acres).

Using district names as the common variable in all three datasets, the variables from these three datasets were merged into a single dataset ^26^. Any errors due to district spellings and duplicate district names across differing states were handled with caution to ensure proper merging. For each district we estimated the population of poor by multiplying the census figures for population of the district by the proportion of the population in the fourth and fifth wealth quintiles (from NFHS4). This was based on the assumption that subsistence cereal consumption is largely restricted to poor small land-holding farmers ^27, 28^. Since, Sorghum and other millets are largely cultivated by poor farmers with small land holdings for subsistence purposes with the exception of economically better-off and well-irrigated regions, particularly in northern India ^28– 30^ District Subsistence Cultivation Quantum (DSCQ) for each district was obtained by multiplying the per capita area (cereal cultivation area in acres/total population) by the proportion of the poor (in the lowest two wealth quintiles as per NFHS) followed by normalising data using logarithmic transformation.

***Independent variables.*** We used the DSCQ for each cereals and other sociodemographic variables listed above while describing the district level dataset. Other socio-demographic variables included in our analysis have been listed above while describing the district level dataset.

**Outcome variables:** percentage of children with wasting (Z score of weight for height <2), percentage of children with stunting (Z score of height for age <2).

### Analysis

***Spatial malnutrition patterns.*** We assessed overlaps between high prevalence of stunting and/or wasting with cereal cultivation data by generating maps derived from The Database of Global Administrative Areas (GADM) ^31^. We merged tabular data (from a spreadsheet file) with geographic data (from a geojson file), chose variables of interest, created map legends dynamically and rendered multiple maps using a custom-built wrapper software written in javascript which internally uses Mapbox GL JS library (version 1.10.0) for rendering maps ^32^. Further information on what this software wrapper does and how it works is present in the README file of the source code ^33^. As a base layer, DSCQ was shaded using a linear interpolator with manually chosen colour levels for the legend. A transparent layer of outcome variables (stunting and wasting) marked with distinct stripe patterns was overlaid on the base layer for visualizing overlap.

***Examining relationship between subsistence millet cultivation, childhood malnutrition and its early onset.*** For each cereal, we examined its association with district-level prevalence of stunting and wasting and DSCQ (normalised using logarithmic transformation) by linear regression. We also examined the relationship of age with wasting and stunting at the district level.

## Results

**Figure 1.**
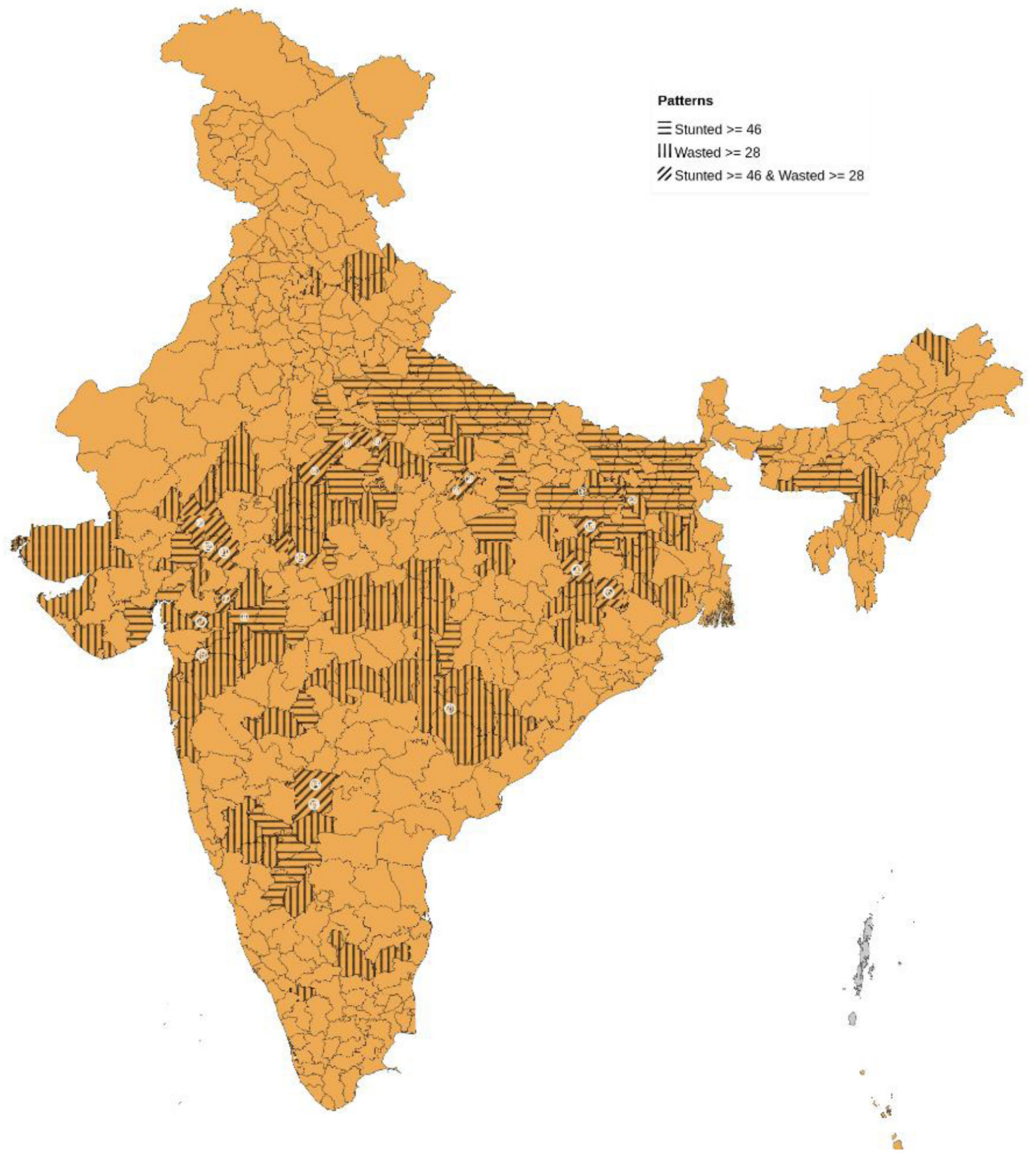
Map of India showing areas with higher prevalence of stunting (>46%) in horizontal bars and those with higher prevalence of wasting (>28%) in vertical bars. Districts with higher prevalence of both stunting and wasting are numbered cross referenced to Table 1 and marked with oblique bars.

In all, 107 districts had a high prevalence of stunting (ranging from 46–65% district prevalence) with risk concentrated in poorer states: Uttar Pradesh (31; 29%) Bihar (28; 26%) and Madhya Pradesh (13; 12%) (numbers in brackets are number of districts followed by percentage). Among the 112 districts, those with higher rates of wasting (ranging from 28–47% district prevalence) were in districts with pre-dominantly tribal population in Jharkhand (14; 12.5%), Madhya Pradesh (20; 17.8%), Maharashtra (12; 11%), Rajasthan (11; 9.8%) and Gujarat (10; 9%) (numbers in brackets are number of districts followed by percentage). High stunting areas were concentrated in north and eastern India, whereas areas of high wasting were primarily in central India, which had high prevalence of both childhood stunting and wasting ( Figure 1). There were 21 districts with high levels of both stunting and wasting, of which 16 had millets of any type or maize as either the most dominant (n=6) or second most dominant crop (n=10) ( Table 1). Of these 21 districts, there were three from Rajasthan, which had more maize cultivation than any other cereal crop: Udaipur (62 %), Banswara (51%), and Dungarpur (47%).

**Table 1.** Districts with high prevalence of stunting(>46%) and high prevalence of wasting(>28%) as per the integrated dataset from NFHS4 and agriculture statistics ^26^.

S.No	District	State	Major crop1	Percentage	Major crop 2	Percentage	Major crop 3	Percentage
1	Arwal	Bihar	Rice	73.64	Wheat	25.21	Maize	0.9
2	Sheikhpura	Bihar	Wheat	49.37	Rice	49.12	Maize	1.5
3	Narayanpur	Chhatisgarh	Rice	87.17	Other millets	7.66	Maize	3.8
4	Narmada	Gujarat	Rice	59.38	Jowar	27.07	Wheat	7.86
5	The Dangs	Gujarat	Rice	64.86	Other millets	16.98	Jowar	16.60
6	Chatra	Jharkhand	Rice	78.44	Maize	11.7	Wheat	8.99
7	Pashchimi Singhbhum	Jharkhand	Rice	98.89	wheat	0.61	Maize	0.47
8	Gumla	Jharkhand	Rice	88.18	Ragi	6.5	Other cereals	2.4
9	Gulbarga	Karnataka	Jowar	86.11	Bajra	5.10	Wheat	3.89
10	Yadgir	Karnataka	Rice	62.26	Jowar	24.99	Bajra	12.35
11	Alirajpur	Madhya Pradesh	Maize	42.4	Wheat	21.99	Bajra	14.42
12	Barwani	Madhya Pradesh	Maize	35.02	Wheat	33.77	Jowar	26.04
13	Bhind	Madhya Pradesh	Wheat	67.57	Bajra	22.00	Jowar	4.06
14	Morena	Madhya Pradesh	Wheat	50.46	Bajra	48.11	Rice	0.44
15	Shajapur	Madhya Pradesh	Wheat	99.44	Jowar	0.53	Rice	0.02
16	Sheopur	Madhya Pradesh	Wheat	66.24	Rice	21.57	Bajra	10.58
17	Banswara	Rajasthan	Maize	51.63	Wheat	34.42	Rice	12.17
18	Dungarpur	Rajasthan	Maize	47.50	Wheat	35.06	Rice	13.19
19	Udaipur	Rajasthan	Maize	62.65	Wheat	29.25	Jowar	2.62
20	Chitrakoot	Uttar Pradesh	Wheat	62.43	Bajra	13.36	Rice	11.23
21	Kaushambi	Uttar Pradesh	Wheat	53.69	Rice	35.95	Bajra	6.33

**Figure 2.**
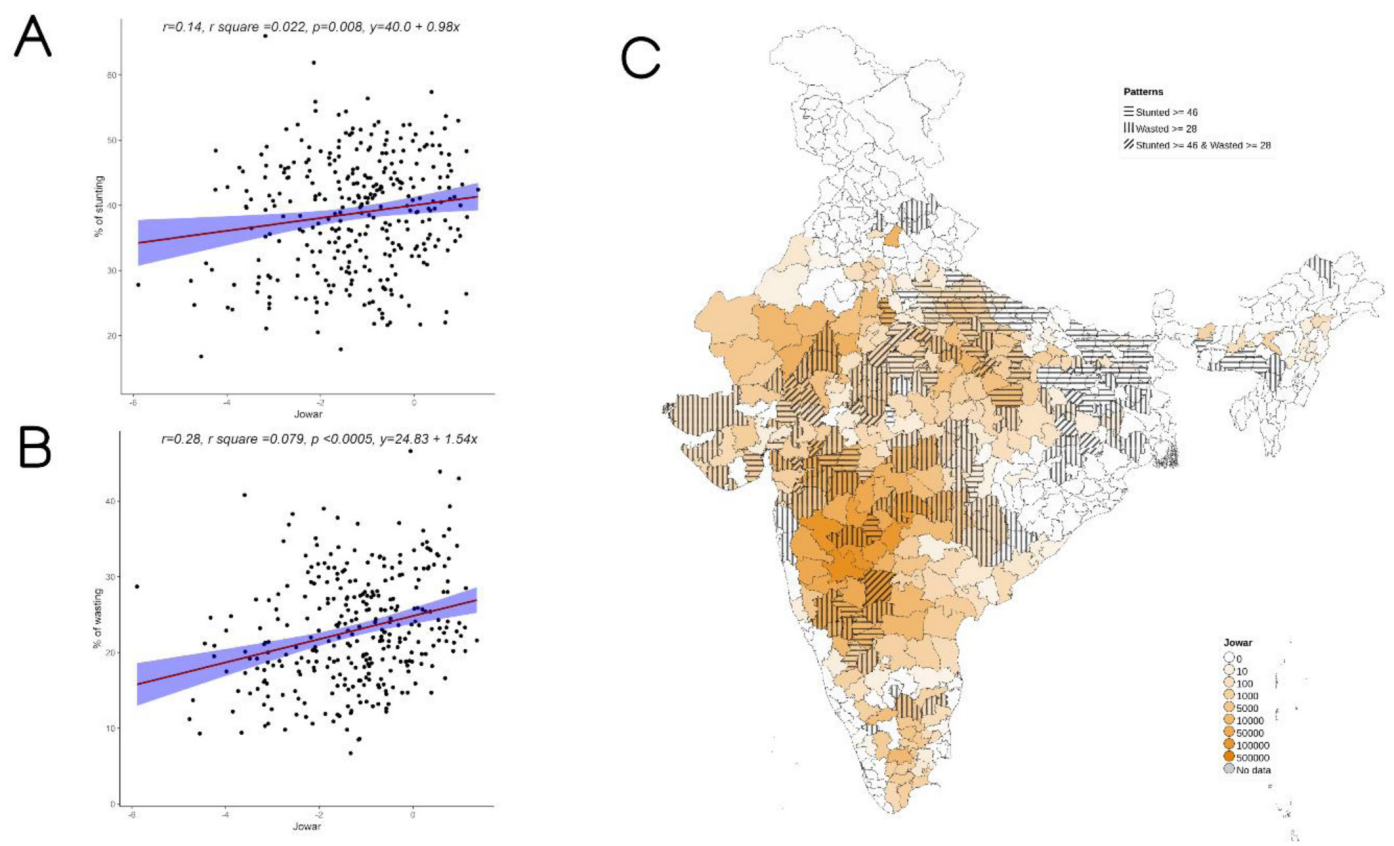
Plots examining relationship between jowar cultivated with stunting and wasting at district level along with map showing the overlap of jowar cultivated with stunting and wasting. **A**) Scatterplot of stunting v/s district subsistence cultivation quantum (DSCQ) of jowar by poor **B**) Scatterplot of wasting v/s DSCQ of jowar by poor **C**) Geographic distribution of DSCQ of jowar by poor, stunting > 46 & wasting >28.

**Figure 3.**
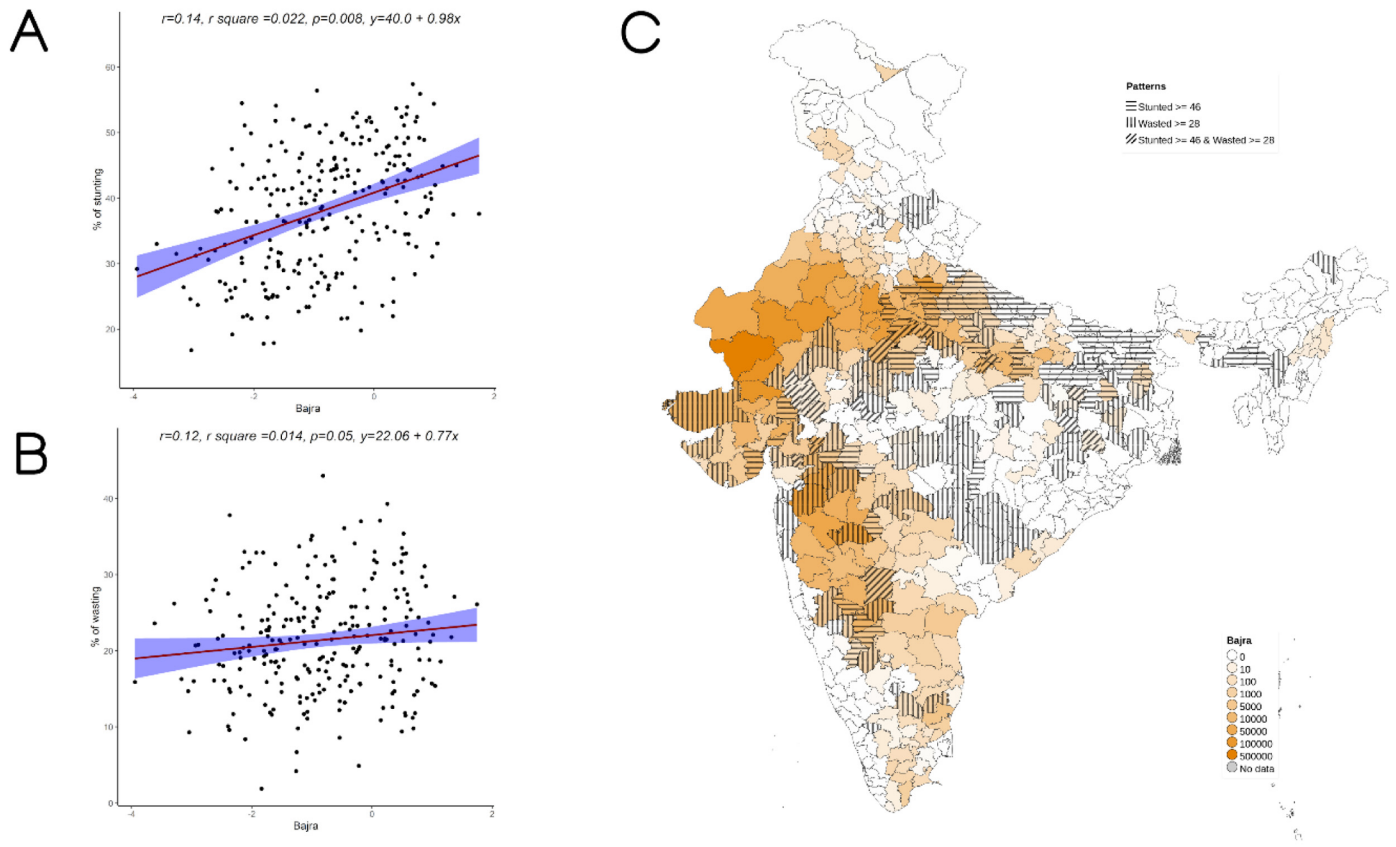
Plots examining relationship between bajra cultivated with stunting and wasting at district level along with map showing the overlap of bajra cultivated with stunting and wasting. **A**) Scatterplot of stunting v/s DSCQ of bajra by poor **B**) Scatterplot of wasting v/s DSCQ of bajra by poor **C**) Geographic distribution of DSCQ of bajra by poor, stunting > 46 & wasting >28.

On examining the district-level patterns of subsistence cultivation of jowar by district overlaid over districts having higher prevalence of stunting and wasting, we find that there is an overlap of districts with wasting alone and those with stunting and wasting with higher DSCQ for jowar ( Figure 2). However, large areas with higher DSCQ particularly in North, West and some parts of central India do not show either stunting or wasting. Similar maps, separately showing overlap of high stunting and high wasting with per-capita cultivation of jowar, wheat, rice, bajra, maize, and other millets are also available ^33^. There is an overlap of districts with high wheat and rice cultivation in the well irrigated Gangetic plain (North and Eastern parts) with stunting ( Figure 5 & Figure 6). Cultivation of other millets is scattered throughout the country with an overlap with high prevalence of wasting. The large, irrigated areas in the Northwest & Central India with high DSCQ of Bajra & Jowar also have higher DSCQ of rice and wheat as seen in Figure 2, Figure 3, Figure 5 & Figure 6. Maize cultivation is all over the country with no clear overlap with either stunting or wasting.

Overall, increase in cultivation of jowar, bajra and other millets is independently associated with increase in prevalence of both stunting and wasting (see Figure 3– Figure 5). When the association was examined for individual millets, whereas jowar cultivation did show an association with increase in both stunting and wasting, increase in bajra cultivation was associated only with increase in stunting. Increase in cultivation of other millets was associated with increase in wasting only (a reverse trend was seen with stunting). As expected, there was either no change or decrease seen when we examined association between increase in rice or wheat cultivation with wasting (with an increase in stunting associated with increase in rice or wheat cultivation).

**Figure 4.**
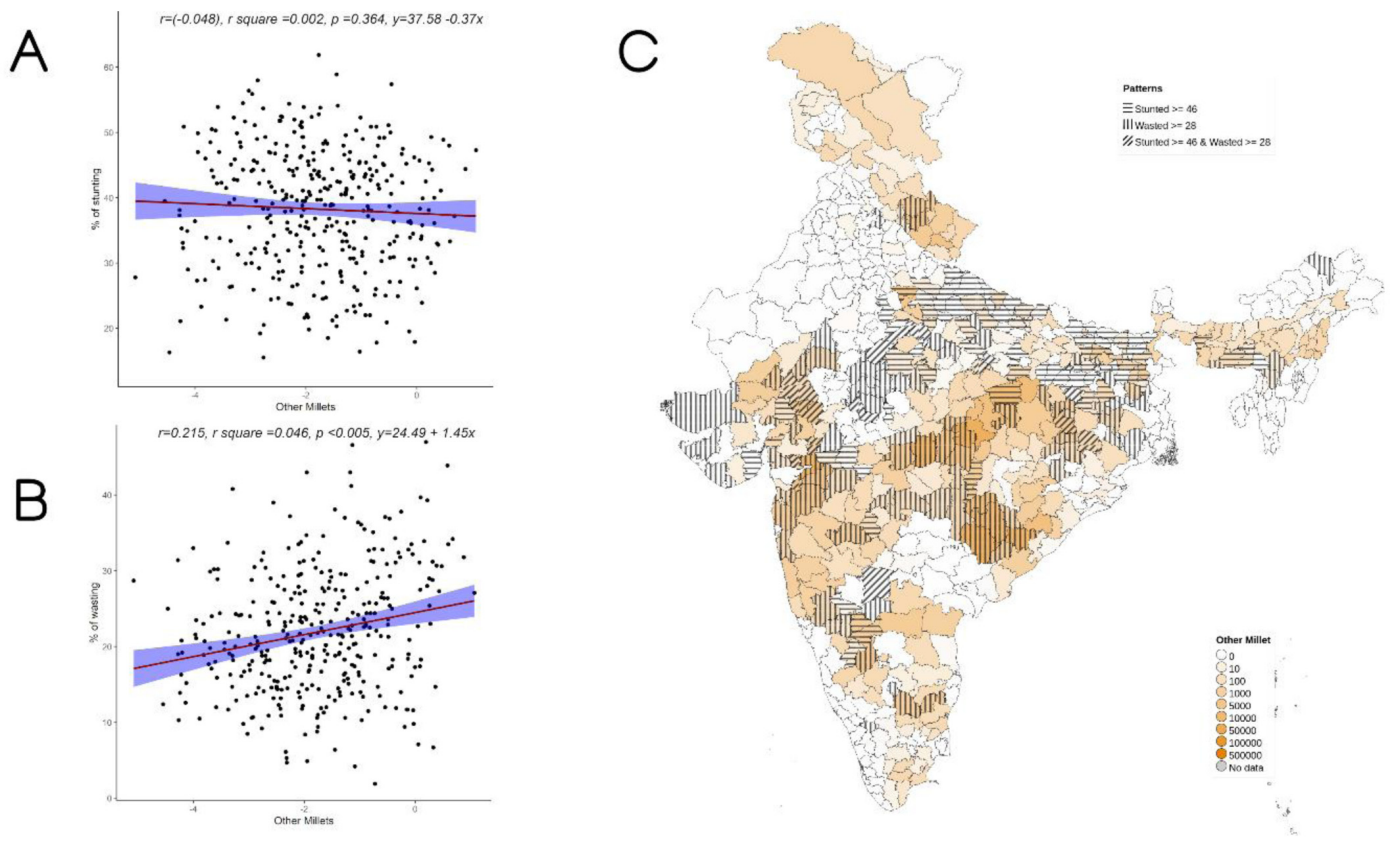
Plots examining relationship between Other millets (kodo millet, little millet, proso millet, barnyard millet and foxtail millet) cultivated with stunting and wasting at district level along with map showing the overlap of Other millets cultivated with stunting and wasting. **A**) Scatterplot of stunting v/s DSCQ of other millets by poor **B**) Scatterplot of wasting v/s DSCQ of Other millets by poor **C**) Geographic distribution of DSCQ of Other millets by poor, stunting > 46 & wasting >28.

**Figure 5.**
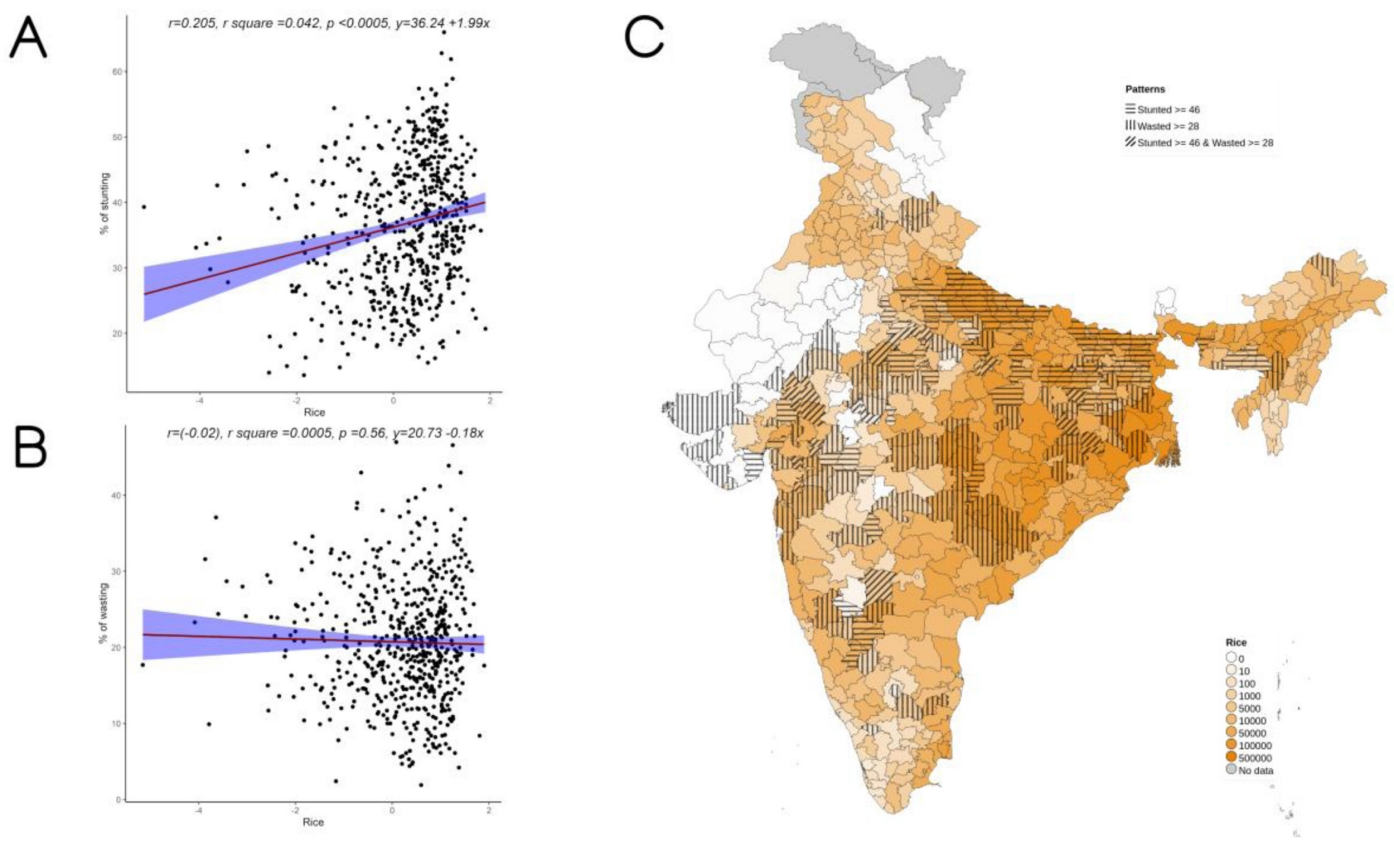
Plots examining relationship between rice cultivated with stunting and wasting at district level along with map showing the overlap of rice cultivated with stunting and wasting. **A**) Scatterplot of stunting v/s DSCQ of rice by poor **B**) Scatterplot of wasting v/s DSCQ of rice by poor **C**) Geographic distribution of DSCQ of rice by poor, stunting > 46 & wasting >28.

**Figure 6.**
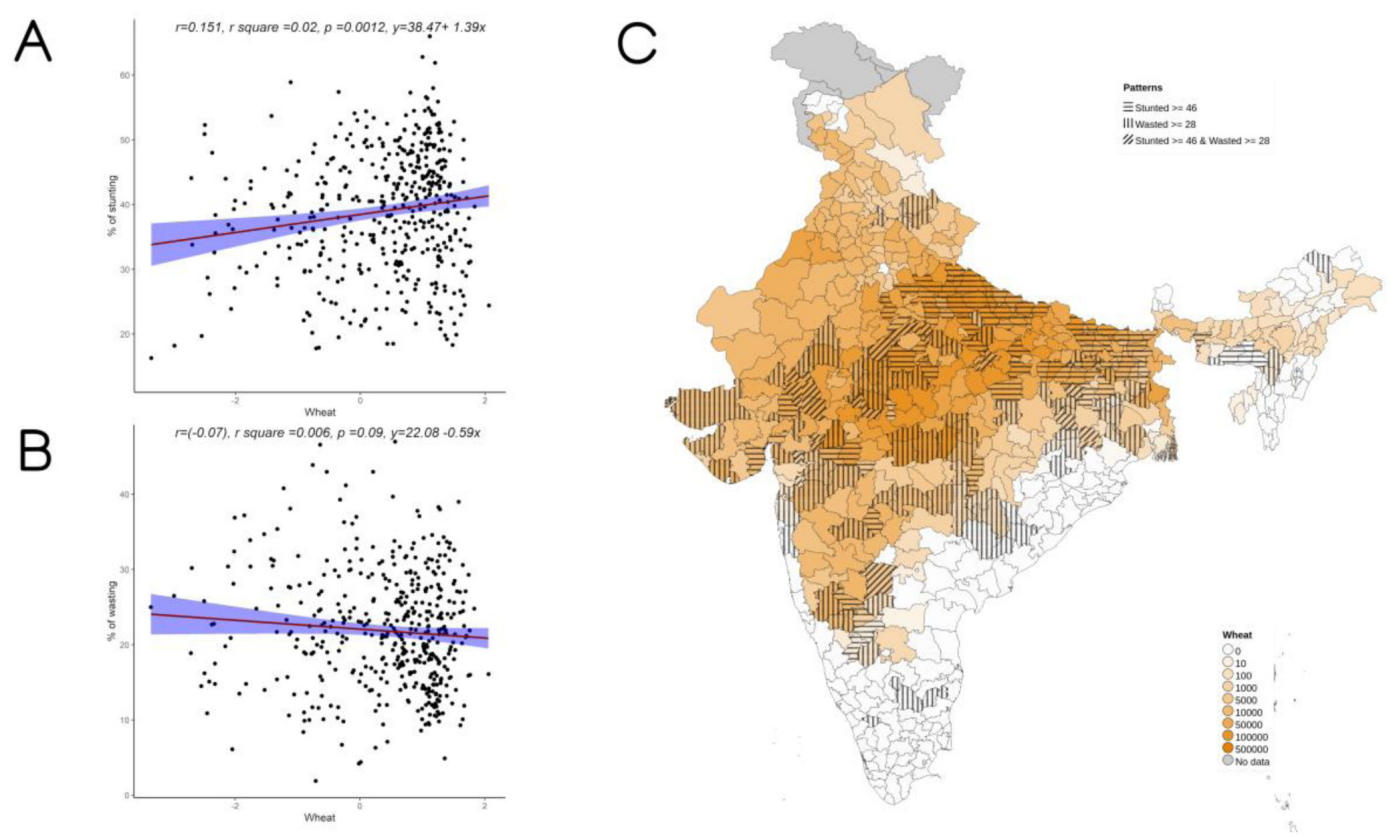
Plots examining relationship between wheat cultivated with stunting and wasting at district level along with map showing the overlap of wheat cultivated with stunting and wasting. **A**) Scatterplot of stunting v/s DSCQ of wheat by poor **B**) Scatterplot of wasting v/s DSCQ of wheat by poor **C**) Geographic distribution of DSCQ of wheat by poor, stunting > 46 & wasting >28.

**Figure 7.**
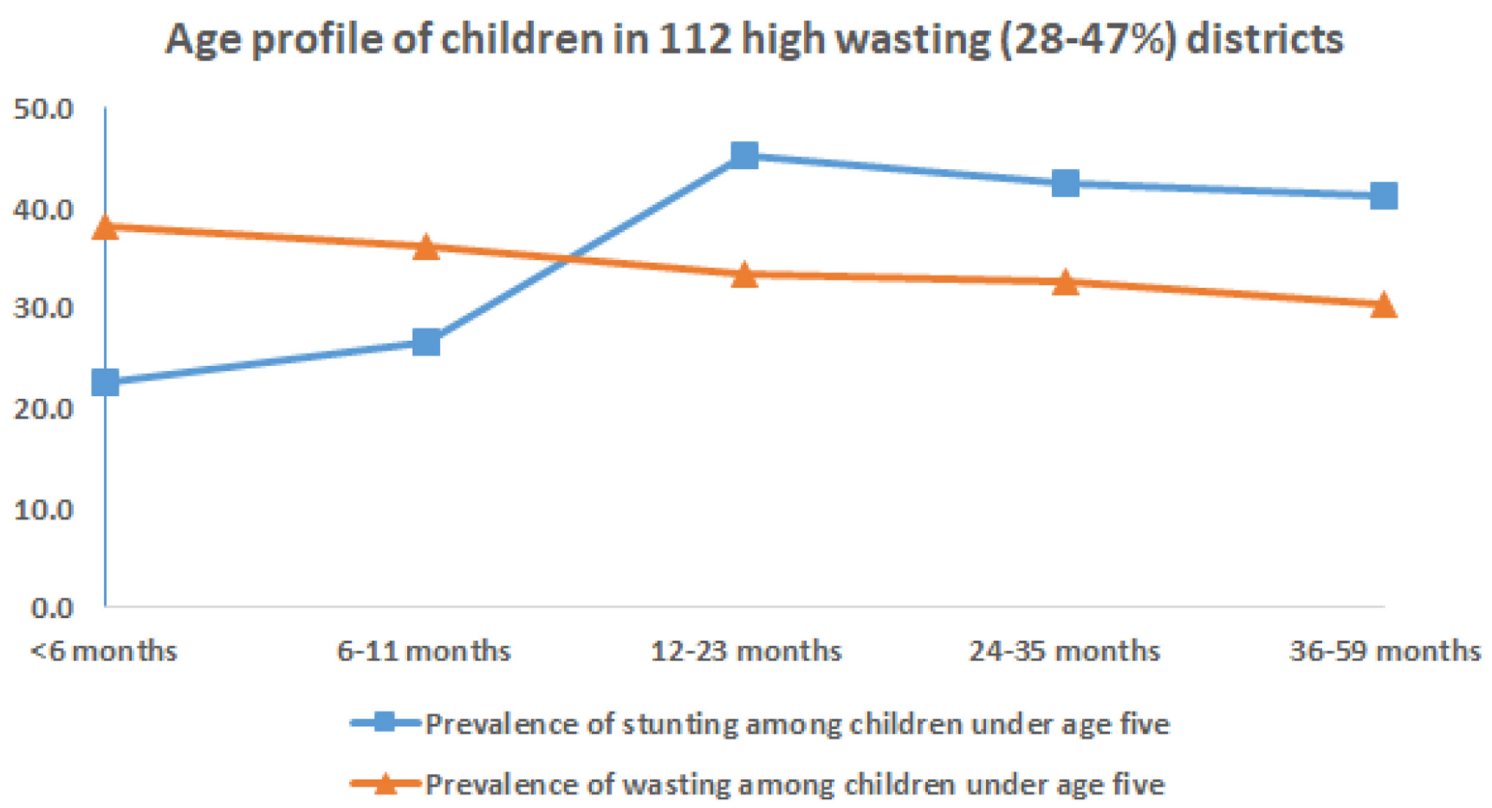
Age profile of stunted and wasted children in 108 high wasting (28–47%) districts.

**Figure 8.**
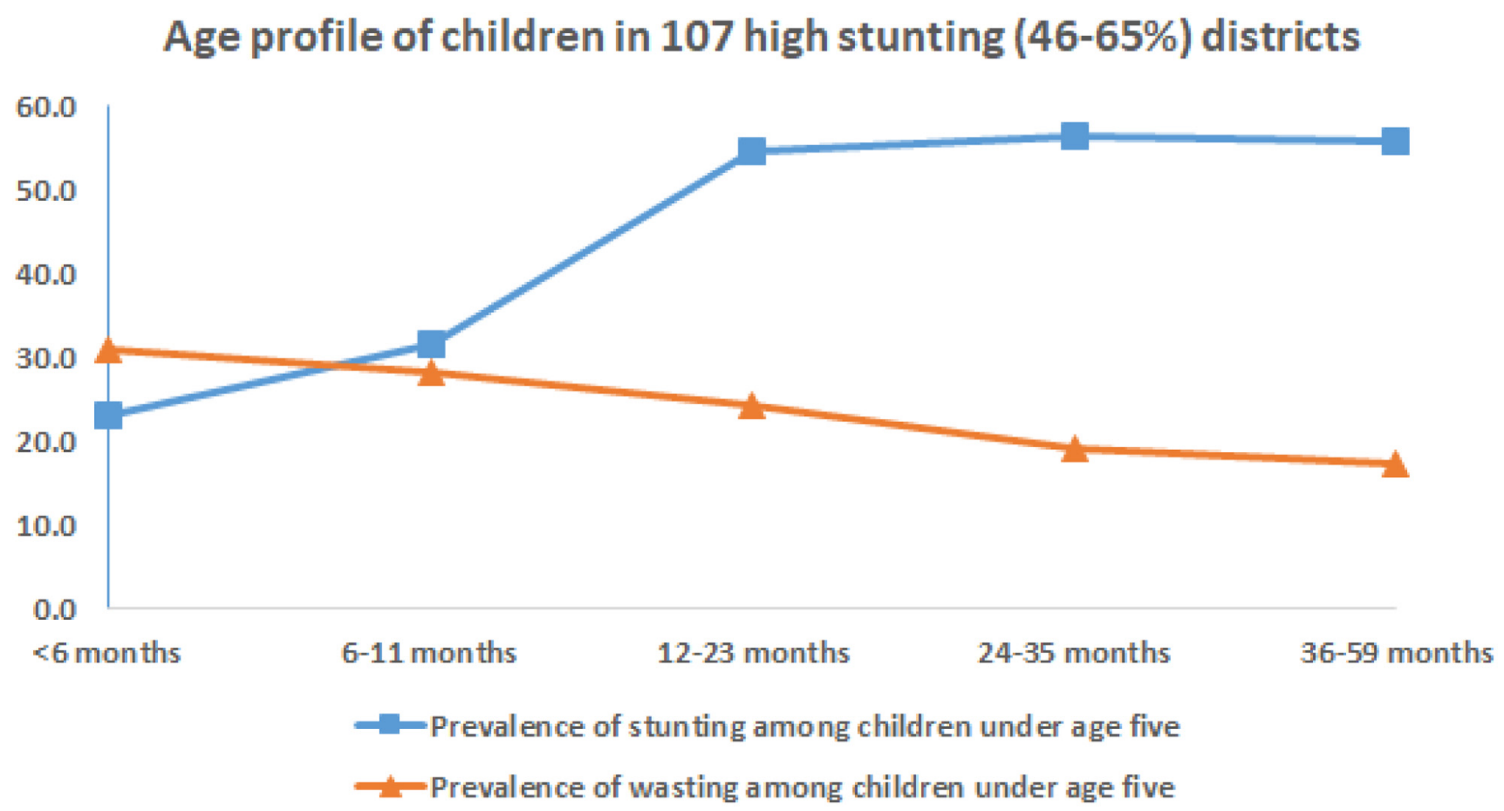
Age profile of stunted and wasted children in 112 high stunting (46–67%) districts.

On examining the age of children in districts with higher prevalence of stunting and wasting the following observations are evident, as seen in Figure 7& Figure 8. In 112 districts with high wasting, wasting showed an early onset with highest wasting (40%) less than 6 months of age ( Figure 7). The age-distribution of stunting was similar for both groups of districts with highest age-specific stunting prevalence at 12 months and a plateau thereafter till five years of age ( Figure 7& Figure 8). The earlier onset of wasting indicates the possibility of maternal nutritional factors affecting intra-uterine growth during pregnancy and possibly continuing in early infancy while being breast fed.

After accounting for the effects of known co-variates of child malnutrition, cultivation of jowar (β = 0.32, 95% CI: 0.163 - 0.488), other millets (β = 3.372, 95% CI: 1.404 - 5.341), women BMI less than 18.5 (β = 0.211, 95% CI: 0.135 - 0.287) are significant predictors of under-five wasting at district level ( Table 2). On the other hand, cultivation of wheat (β = 0.315, 95% CI:0.191 - 0.439), and women short stature (β = 0.474, 95% CI: 0.376 - 0.571) predict district level stunting ( Table 3)

**Table 2.** Multivariable Regression models exploring the association between poor, women =>10 years of education, Proportion of rural, Open defecation, Minimum dietary diversity, Utilization of anganwadi, Women’s short stature (<145 cms) in 15-49 years of age, Women’s BMI less than 18.5 in the 15-49 years of age, cultivation of Jowar, Bajra, other millets, rice and ragi and the outcome of interest is under 5 wasting.

Variables	Model-1	Model-2	Model-3	Model-4	Model-5	Model-6	Model-7
	Unadjusted coefficients (95% CI)	Adjusted coefficients (95% CI)	Adjusted coefficients (95% CI)	Adjusted coefficients (95% CI)	Adjusted coefficients (95% CI)	Adjusted coefficients (95% CI)	Adjusted coefficients (95% CI)
Poor	0.073 ***	-0.029				0.005	-0.025
	(0.050 - 0.095)	(-0.072 - 0.014)				(-0.036 - 0.047)	(-0.072 - 0.022)
women =>10 years of education	-0.074 ***	-0.007				0.016	0.016
	(-0.105 - -0.042)	(-0.049 - 0.035)				(-0.026 - 0.058)	(-0.027 - 0.058)
Proportion of rural	0.030 *	-0.042 *				-0.075 ***	-0.058 ***
	(0.002 - 0.058)	(-0.075 - -0.008)				(-0.107 - -0.042)	(-0.092 - -0.025)
Open defecation	0.125 ***	0.157 ***				0.120 ***	0.070 ***
	(0.106 - 0.145)	(0.129 - 0.185)				(0.092 - 0.148)	(0.039 - 0.101)
Minimum dietary diversity	-0.168 ***		-0.173 ***			-0.119 ***	-0.056 **
	(-0.206 - -0.129)		(-0.210 - -0.136)			(-0.157 - -0.080)	(-0.099 - -0.014)
Utilization of anganwadi	0.093 ***		0.098 ***			0.088 ***	0.046 **
	(0.064 - 0.122)		(0.070 - 0.125)			(0.061 - 0.115)	(0.016 - 0.075)
Women short stature	0.026						
	(-0.073 - 0.125)						
Women BMI less than 18.5	0.372 ***			0.372 ***			0.211 ***
	(0.321 - 0.422)			(0.321 - 0.422)			(0.135 - 0.287)
Jowar	0.620 ***				0.670 ***		0.326 ***
	(0.482 - 0.757)				(0.500 - 0.840)		(0.163 - 0.488)
Bajra	0.228 **				-0.306 ***		-0.194 *
	(0.085 - 0.371)				(-0.481 - -0.131)		(-0.366 - -0.022)
Wheat	0.363 ***				0.365 ***		0.135
	(0.243 - 0.483)				(0.243 - 0.488)		(-0.003 - 0.272)
Other millets	6.689 ***				4.299 ***		3.372 ***
	(4.472 - 8.905)				(2.185 - 6.412)		(1.404 - 5.341)
Rice	0.052						
	(-0.128 - 0.233)						
Ragi	0.490 ***				0.488 ***		0.237 *
	(0.296 - 0.684)				(0.303 - 0.674)		(0.057 - 0.418)
Observations	640	640	640	640	640	640	640
R-squared		0.218	0.167	0.247	0.221	0.299	0.382

*** p<0.001, ** p<0.01, * p<0.05

**Table 3.** Multivariable Regression models exploring the association between poor, women =>10 years of education, Proportion of rural, Open defecation, Minimum dietary diversity, Utilization of anganwadi, Women short stature (<145 cms) in 15–49 years age, Women’s BMI less than 18.5, cultivation of Jowar, Bajra, other millets, rice and ragi and the outcome of interest is under 5 stunting.

Variables	Model-1	Model-2	Model-3	Model-4	Model-5	Model-6	Model-7
	Unadjusted coefficients (95% CI)	Adjusted coefficients (95% CI)	Adjusted coefficients (95% CI)	Adjusted coefficients (95% CI)	Adjusted coefficients (95% CI)	Adjusted coefficients (95% CI)	Adjusted coefficients (95% CI)
Poor	0.248 ***	0.052 *				0.062 **	**0.031**
	**(0.225 - 0.271)**	**(0.010 - 0.093)**				**(0.023 - 0.102)**	**(-0.014 - 0.076)**
women =>10 years of education	-0.335 ***	-0.197 ***				-0.137 ***	-0.104 ***
	**(-0.367 - -0.304)**	**(-0.237 - -0.156)**				**(-0.177 - -0.097)**	**(-0.143 - -0.065)**
Proportion of rural	0.164 ***	-0.044 **				**-0.030**	**-0.012**
	**(0.130 - 0.198)**	**(-0.077 - -0.012)**				**(-0.061 - 0.001)**	**(-0.043 - 0.019)**
Open defecation	0.235 ***	0.146 ***				0.132 ***	0.086 ***
	**(0.214 - 0.257)**	**(0.119 - 0.173)**				**(0.106 - 0.159)**	**(0.057 - 0.114)**
Minimum dietary diversity	-0.339 ***		-0.336 ***			-0.149 ***	-0.082 ***
	**(-0.384 - -0.294)**		**(-0.381 - -0.292)**			**(-0.186 - -0.113)**	**(-0.121 - -0.043)**
Utilization of anganwadi	-0.058 **		-0.048 **			-0.068 ***	-0.038 **
	**(-0.096 - -0.019)**		**(-0.081 - -0.015)**			**(-0.093 - -0.042)**	**(-0.065 - -0.011)**
Women short stature	0.902 ***			0.612 ***			0.474 ***
	**(0.796 - 1.007)**			**(0.512 - 0.712)**			**(0.376 - 0.571)**
Women BMI less than 18.5	0.571 ***			0.427 ***			**0.065**
	**(0.511 - 0.631)**			**(0.368 - 0.487)**			**(-0.005 - 0.134)**
Jowar	0.511 ***				0.265 *		**0.079**
	**(0.328 - 0.694)**				**(0.053 - 0.476)**		**(-0.072 - 0.230)**
Bajra	0.467 ***				**-0.052**		**0.146**
	**(0.285 - 0.648)**				**(-0.274 - 0.170)**		**(-0.011 - 0.304)**
Wheat	0.964 ***				0.901 ***		0.315 ***
	**(0.825 - 1.103)**				**(0.749 - 1.052)**		**(0.191 - 0.439)**
Other millets	**2.039**						
	**(-0.875 - 4.954)**						
Rice	0.420 ***				0.361 ***		**-0.102**
	**(0.191 - 0.650)**				**(0.153 - 0.570)**		**(-0.258 - 0.055)**
Ragi	**-0.222**						
	**(-0.475 - 0.030)**						
Observations	**640**	**640**	**640**	**640**	**640**	**640**	**640**
R-squared		**0.557**	**0.266**	**0.473**	**0.246**	**0.617**	**0.684**

*** p<0.001, ** p<0.01, * p<0.05

## Discussion

Poverty and its antecedents affecting dietary intake, healthcare and poor environmental conditions can broadly explain malnutrition prevalence, particularly stunting worldwide ^2^. Nearly three decades ago, Victora identified the several-fold variation in wasting prevalence in areas with similar stunting prevalence, also highlighted more recently by Black *et. al.* (2008) ^17, 34^. In a particularly illuminating paper, Martorell *et al.* (2012) highlighted higher levels of early wasting with similar levels of stunting in India, in comparison to Gautemala ^35^. They have linked the higher prevalence of low BMI and maternal anaemia in India, while suggesting that improvements in maternal nutrition through prenatal interventions and during breastfeeding could address this problem of early wasting. Our analysis attempts to show that ultimately, disaggregation of the data on type of malnutrition prevalence within India at a finer-scale along with proximate dietary factors in terms of staple cereal consumption holds the clues to such differences.

### Global patterns of wasting linked to millet subsistence

The explanations for ecogeographic patterns of wasting in India appear to at least partially rest in subsistence cultivation patterns and staple consumption of millets as seen in our results. This pattern can be reproduced globally. Subsistence farming of millets overlaps with other global regions with some of the highest prevalence of wasting worldwide in Yemen and Sub-Saharan Africa ^3, 30^. Similarly, in the low-lying areas of Jizan province of Saudi Arabia (which is adjoining Yemen), pearl millet is cultivated and widely used as a staple; expectedly, wasting prevalence in Jizan is the highest among all provinces in Saudi Arabia ^36– 38^. Malnutrition in early infancy (< six months of age) has been shown to be highly prevalent in India, Niger, Nigeria, Burkina Faso and Mali ^39^. These are in fact the top countries that produce millets for human consumption through subsistence farming ^40^. A paper by Grellety and Golden ^41^ brought out differences in patterns of wasting in countries with some having more proportion of wasting assessed by MUAC, while others had greater proportion of wasting represented by low weight for height or having both low weight for height and low MUAC. On examining FAOSTAT data ^40^ to explain Grellety and Golden’s assessment of differences in types of acute malnutrition across various countries, we found higher prevalence of wasting by MUAC to be a feature of maize-cultivating countries, while the countries reporting low weight for height are typically cultivating millets ^40– 42^. These apparent differences in type of malnutrition linked to cereals has also been reproduced in observational studies. In a comparison of Bwamanda district (Democratic Republic of Congo with maize and Cassava staple) and Niakhar region (in Senegal with staple millet consumption), the former had higher proportion of wasting measured by lower MUAC, while the latter had earlier onset of wasting and higher prevalence of low weight for heights ^43^. At a global level, the top 50 countries ranked among the hidden hunger scores ^44^ had either maize or millets/sorghum as staple among the top two cereals (as seen from FAOSTAT data of the top two cereals produced), an observation that may be linked to protein quality of these cereal crops ^40, 42^. 

### Cereal protein quality

Sorghum, millets and maize share a common evolutionary ancestor in the grass family (Family *Poaceae*, sub-family *Panicoideae*), and can grow in arid/semi-arid agro-climatic regions where other crops often do not produce optimal yields through their dependence on the C4 carbon fixation pathway of photosynthesis ^45– 48^. Millets are also usually not traded in markets but consumed directly by poor subsistence farmers ^28, 29^. In rural India, the chief source of proteins are cereals ^49^. Further, there is a socio-economic gradient to protein quality; tribal populations and the poor consume lesser lysine-containing proteins primarily through cereals ^49, 50^. The diet of rural pregnant and lactating women is particularly inadequate with respect to quality of protein, thereby contributing to early malnutrition. Lysine content of millet (22 mg/g of protein) and sorghum (24 mg/g of protein) is the least among cereals in comparison to rice (35 mg/g of protein) and wheat (27 mg/g of protein) ^49^. The proportionate amino-acid requirement at infancy is the highest and shows an age-related decline ^49– 53^. Extensive dependence on millets and sorghum as a staple among millions of poor rural communities where subsistence farming is the mainstay, in semi-arid and rainfed agricultural landscapes, motivated the FAO to commission a detailed assessment of their dietary protein quality ^30^. The report unequivocally highlights the inadequacy of millet and sorghum proteins for infants and young children based on amino acid scores ^30^. The other inexpensive and subsistence crop in India is maize with only 20% consumption, which (apart from its association with Kwashiorkor in the initial description by Cecily Williams) too has been extensively investigated for its causation of Pellagra ^54– 57^. Similarly, Sorghum has been shown to be associated with Pellagra in Indian studies ^16, 58^. Protein quality is assessed currently by the Digestible Indispensable amino acid scores(DIAAS) as per guidelines of the FAO ^59^. The DIAAS of different cereals is shown in Table 4, drawn from a compendium curated by Hans-Henrik Stein of the University of Illinois, Urbana-Champaign (pers comm).

**Table 4.** Digestible indispensable amino acid scores (DIAAS) determined for human foods using the pig or rat model (Data from published studies compiled by Hans-Henrik Stein, University of Illinois, Urbana-Champaign).

		Reference Protein pattern			
Cereal grains	Animal model/ human	Infants(0–6 months	Young children (6 months–3 years)	Older children, adolescents and adults	Reference
Rice, cooked	Rat	--	60 (lysine)	--	Rutherfurd *et al.*, 2015
Rice, polished, cooked	Rat	--	37 (lysine)	--	Han *et al.*, 2019
Rice protein	Pig		48 (lysine)	57 (lysine)	Exp. 620 ^#^
Rice, white, polished, raw	Pig	--	--	64 (lysine)	Cervantes-Pahm *et al.*, 2014
Sorghum, raw	Pig	--	--	29 (lysine)	Cervantes-Pahm *et al.*, 2014
Millet, foxtail, cooked	Rat	--	10 (lysine)	--	Han *et al.*, 2019
Millet, proso, cooked	Rat	--	7 (lysine)	--	Han *et al.*, 2019
Corn, yellow dent, raw	Pig	--	--	48 (lysine)	Cervantes-Pahm *et al.*, 2014
Wheat, whole, cooked	Rat	--	20 (lysine)	--	Han *et al.*, 2019

^#^ Exp 620 stands for Experiment 620 in the Laboratory of Prof Hans-Henrik Stein, University of Illinois, Urbana-Champaign

Sorghum protein is stored in Kafirins and is deficient in amino acids lysine with an excess of leucine ^16, 21, 30, 58^, Millets in general have higher tannins ^21, 30^; pearl millet has antinutrients like phytic acid, goitrogenic polyphenols, and tannins ^60^. Despite its better amino-acid profile (among the millets) the digestibility of proteins in Pearl millet (bajra) is probably less than other major grains ^21^ due to antinutrients ^60^. 

**Effect of cooking and the dietary matrix**: In India, the commonest mode of consumption of millets is by milling followed by removal of bran and making unleavened bread using (typically) dry heat ^61^. Porridge-like preparations and cooked grains are also common. Sorghum protein becomes much less soluble after cooking ^62^. These modes of processing are inferior to processes such as fermentation, germination or in combination which increase the availability of micronutrients, such as iron and zinc ^63, 64^. Such cultural and social norms are important determinants of bioavailability of nutrients. For instance, maize is consumed in Latin America after nixtamalization, unlike in India where it is consumed directly, possibly explaining the stunting and wasting in the few districts in India where maize-growing for staple consumption is high ^65^. In separate studies for pearl millet and sorghum, conducted at the Hospital for sick children in Toronto (Canada), lysine availability was checked by assessing Indicator amino acid oxidation method (IAAO) after overnight soaking and boiling of the respective cereal in a rice cooker at 90°C. The pearl millet used had a lysine composition of 30.6 g/kg protein while sorghum had 27.31 g/kg protein. The studies yielded availability of cooked Lysine of 97 % for pearl millet and 94% for Sorghum ^66– 68^. However, the above method of cooking is significantly different from that used commonly in India for both sorghum and pearl millet. The flour is cooked by dry heat (at temperatures in the range 200°C-300°C) to make unleavened bread (variously called *bhakri* or *roti*) ^69– 72^. Heat is known to adversely affect cereal protein availability by means of *Maillard reaction* and Lysinoalanine-like product formation, particularly in sorghum ^70, 73^. So, the already lower lysine (as per composition per kg of protein) can get compromised further ^73^. In southern India, porridge like preparations are more common ^69^. 

A study using stable isotope method with Indian rice-, wheat- and *ragi*-based meals for iron studies showed the least absorption with millet-based meals among the three. The study also indicates the importance of dietary matrix for iron absorption, even in cereals like *ragi*, which is replete in micronutrients like iron and calcium ^74^. Overall, there is an urgent need for state-of-the-art stable isotope studies in India to determine the metabolic availability of key nutrients from the common diets prevalent among rural poor.

**Ready to use therapeutic food and aminoacids**: The focus on protein quality has important biomedical and policy implications. Recent success with the use of peanut paste with milk based ready-to-use therapeutic food (RUTF) is being supplemented with well-intentioned attempts to use locally available ingredients in community-based malnutrition management approaches ^75, 76^. These soya-maize-sorghum (SMS) formulations have been reported to be inferior in trials, particularly in children less than two years of age *vis-a-vis* peanut-based RUTF ^76, 77^. However, when supplemented with free amino acids (free aminoacid soya-maize-sorghum RUTF), it has been shown to be as efficacious as standard peanut milk based RUTF ^78^ thereby highlighting amino acids to be the missing link in the millet-based RUTF in the first 1000 days of life.

**Micronutrient availability from cereals:** However, in a poor family with low dietary diversity on a cereal based diet, in contrast to amino acid availability, the intake of micronutrients, particularly Zinc, could be lesser in the pre-dominantly rice and wheat cultivating areas ( Table 5). Zinc levels, however, remains unaffected by maternal status and intake in the breast milk ^79^. However, calcium and possibly, Vitamin D are affected by maternal intake in the breast milk. Ragi is replete with both Calcium and Vitamin D in comparison to other cereals ( Table 5). Figure 9 has pie charts showing cultivation of cereal crops all over India (A) as well as in districts with high wasting (9B) and high stunting (9C). The figures show that districts with high wasting have higher proportion of cultivation of coarse cereals (jowar, bajra, other millets and maize) with a commensurate reduction in proportion of rice and wheat. The converse is true for the proportion of crops in the 107 districts with high stunting. Hence, the higher prevalence of stunting and short stature in rice- and wheat-growing areas ( Figure 2– Figure 8) could be due to micronutrient deficiencies, particularly of zinc ^80– 82^. 

**Table 5.** Micronutrient table for cereals compiled from Indian Food composition Tables 2017, National Institute of Nutrition, Hyderabad, India ^82^.

						In 100 gm of edible portion		Expressed per 100 gm edible portion		
S.no	Cereal Name	Fe (mg)	Zn (mg)	Ca (mg)	Se (mg)	Total Carotenoids µg	Vit D2 µg	B1 (mg)	B3 (mg)	Total Folates B 9 mg
1	Bajra (Pennisetum typhoideum)	6.42 +/- 1.04	2.76 +/- 0.36	27.35 +/- 2.16	30.4 +/- 5.22	293 +/- 55.7	5.65 +/- 0.27	0.25 +/ 0.04	0.86 +/- 0.10	36.11 +/- 5.05
2	Jowar (Sorghum vulgare)	3.95 +/- 0.94	1.96 +/- 0.31	27.60 +/- 3.71	26.29 +/- 11.08	9.08 +/- 1.77	3.96 +/- 0.30	0.35 +/- 0.039	2.10 +/- 0.09	39.42 +/- 3.13
3	Maize, dry (Zea mays)	2.49 +/- 0.32	2.27 +/- 0.23	8.91 +/- 0.61	8.69 +/- 1.81	893 +/- 154	33.6 +/- 2.82	0.33 +/- 0.032	2.69 +/- 0.06	25.81 +/- 1.44
4	Ragi (Eleusine coracana)	4.62 +/- 0.36	2.53 +/- 0.51	364 +/- 58	15.30 +/- 6.23	154 +/- 25.6	41.46 +/- 3.12	0.37 +/- 0.041	1.34 +/- 0.02	34.66 +/- 4.97
5	Little millet or Samai (Panicum miliare)	1.26 +/- 0.44	1.82 +/- 0.14	16.06 +/- 1.54	40.41 +/- 24.09	120 +/- 9	0.28 +/- 0.80	0.26 +/- 0.042	1.29 +/- 0.02	36.20 +/- 7.04
6	Foxtail millet (Setaria italica)	2.34 +/- 0.46	1.65 +/- 0.18	15.27 +/- 1.28	14.12 +/- 2.26	272 +/- 25.1	-----	0.29 +/- 0.054	1.49 +/- 0.08	39.49 +/- 4.52
7	Rice, raw milled (Oryza sativa)	0.65 +/- 0.11	1.21 +/- 0.17	7.49 +/- 1.26	1.01 +/- 0.13	16.87 +/- 5.61	-----	0.05 +/- 0.019	1.69 +/- 0.13	9.32 +/- 1.93
8	Wheat flour,atta (Triticum aestivum)	1.77 # +/- 0.38	0.88 # +/- 0.07	20.4 # +/- 2.46	-----	284 +/- 31.9	13.43 +/- 1.77	0.42 +/- 0.044	2.37 +/- 0.10	29.22 +/- 1.92
9	Barley (Hordeum vulgare)	1.56 +/- 0.15	1.5 +/- 0.27	28.64 +/- 3.49	18.61+/- 1.32	69.87 +/- 28.88	------	0.36 +/- 0.059	0.86 +/- 0.10	31.58 +/- 3.79

# For wheat flour refined

**Figure 9.**
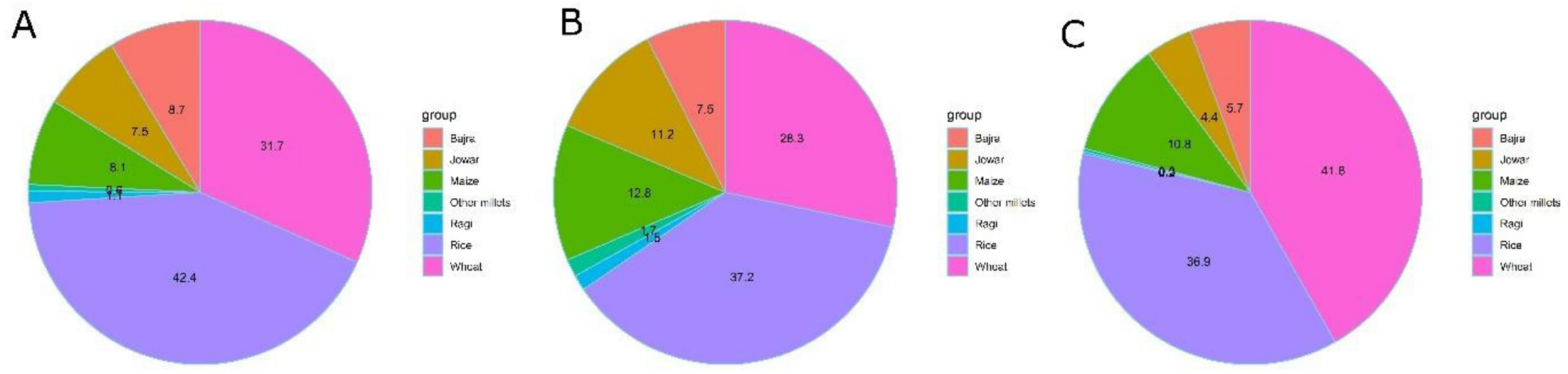
Pie charts showing Proportionate shares of cereal crops in districts with higher stunting (>46%), higher wasting (>28%) and all over India. ( **A**) pie chart showing proportionate share of cereal crops in India as a whole ( **B**) Pie chart showing proportionate share of cereal crops in 112 districts with higher prevalence of wasting ( **C**) Pie chart showing proportionate share of cereal crops in 107 districts with higher prevalence of stunting.

### Stunting, wasting and aminoacid level

A metabolomic study involving hospitalized children with acute malnutrition (kwarshiorkor & marasmus) in Malawi showed lower essential and conditionally essential amino acid levels in comparison to both stunted and non-stunted community controls ^83^. A similar community-based study showed lower amino acid profiles (particularly essential amino acids) among stunted children in comparison to non-stunted controls ^84^. These graded differences have raised questions about the possible causal role of amino acids in stunting ^85^. Similarly, it could be reasoned that further lower intake of amino acids could be linked to wasting in children with Kwarshiorkor and marasmus as seen in the Malawi hospital study. Hence, in a comparable caloric intake among populations consuming different cereal based diets there could be different levels of aminoacids and other key nutrients like glucose (because of varying glycemic indices of cereals ^86^) which are likely to have a bearing on growth as explained below.

### Cellular pathways to malnutrition

Over the last three decades, pioneering research on cell growth has helped elucidate the critical role of complex intracellular nutrient-sensing mechanisms and their linkages with upstream and downstream pathways incorporating endocrine inputs for growth, primarily centred on the role of protein kinases, MTORC 1 and MTORC2 ^87– 92^. Another protein kinase, GCN2 acts in concert with MTORC1 in sensing amino acid deficiencies ^90, 91^. 

From the results above, clearly, there are distinct geographic patterns of both stunting and wasting. There is also similarity in patterns of association between low maternal BMI with child wasting and maternal short stature with child stunting. Large-scale subsistence cultivation of millets in dry/semi-arid areas of central India are more likely to be associated with higher wasting. Other factors like quantity of food, intake of proteins through milk and other animal sources during and before the first 1000 days by the mother, the dietary matrix and diversity and presence of infections are also important. However, the ecogeographic patterns of malnutrition can probably be explained to some extent by staple cereal cultivation. Hence, the proportionate shares of cultivation of cereals crops in the country as whole and in the district with high wasting and stunting is shown in Figure 9

**Figure 10.**
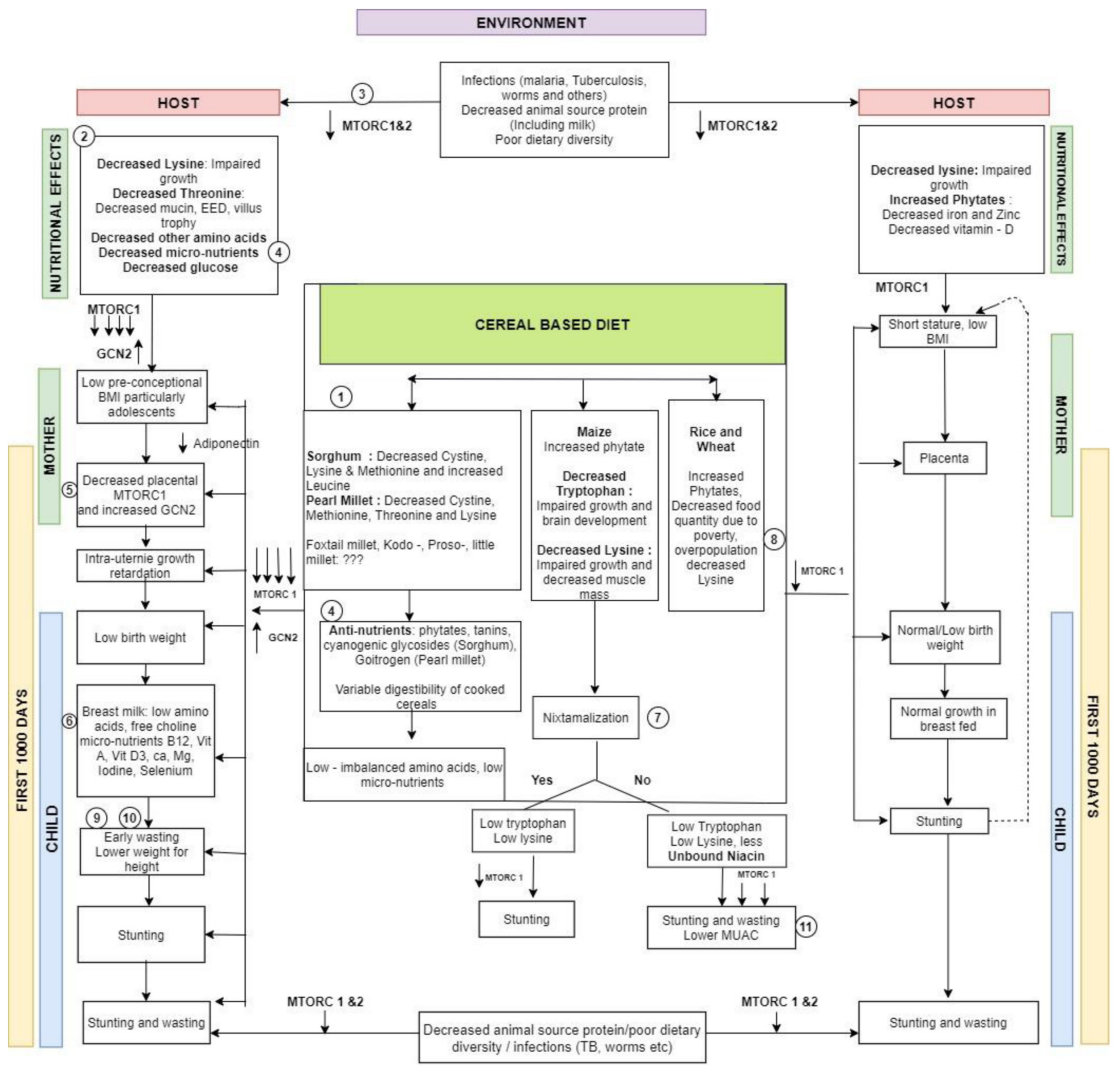
A schematic figure showing the possible pathways linking staple cereal consumption with child malnutrition with key steps highlighted (with references to evidence from literature).

An attempt has been made here to build a hypothetical framework explaining the plausible pathways through which staple based cereal based diet could produce stunting and wasting ( Figure 10)

A pre-dominantly millet diet has a low glycaemic index, putative deficiencies in amino acids and micronutrients with variable digestibility after cooking ( Figure 10 pathway 2) due to the presence of a tough cell wall and antinutrients ^21, 30, 55, 60, 93^, This could lead to a greater lowering of amino acids and glucose, which in turn could lead to a substantial lowering of MTORC1 and rise in GCN2 contributing to wasting ( Figure 10 pathway 5) ^87– 91^. The lowering of MTORC1 & GCN2 at the cellular level causes low BMI in women (particularly growing adolescents) with consequent decreased acquisition of nutrients by placental syncytiotrophoblast ^87, 88, 94, 95^. This in turn leads to intra-uterine growth restriction and low birth weight. These nutritional effects on the malnourished mother persist during breast feeding ^79^ ( Figure 10 pathway 6) leading to early wasting and lower weight for height seen in regions with higher wasting ( Figure 10 pathway 9) ^35, 41, 43, 96, 97^, Maize consumption without nixtamalization could lead to lesser unbound niacin ( Figure 10 pathway 7) ^42, 65, 92, 98^, and both stunting and wasting with lower MUAC ( Figure 10 pathway 7) ^41– 43^. Poverty in highly populous areas growing rice & wheat could be contributory to stunting. Possible greater stress, lower dietary quantity and poor sleep could be contributory ( Figure 10 pathway 8) ^12, 19, 38, 99, 100^. Lower lysine, high phytates with lowered micronutrients like zinc and iron in cereal based diet in wheat and rice growing areas with poor dietary diversity could lead to stunting ( Figure 10 pathway 8) ^8, 18, 19, 98– 100^. Infections in the growing child can decrease stimulation of both MTORC1 and 2 through T cell mediated mechanisms ( Figure 10 pathway 3) ^101^. Millets are indeed gluten-free, have high fibre and antioxidant content and have recently seen a spike in use ^102– 104^. However, their use by pregnant as well as nursing mothers and children in poor communities, with limited dietary diversity, during the first 1000 days could be associated with malnutrition.

### Study limitations

An important limitation of our analysis is the limited fine scale data on low birth weight and food grain consumption (as opposed to cultivation) which would have allowed for confirmation of our hypothesis at household level. One of the reasons for this is that the NFHS and other country/regional demographic health surveys record cereal consumption without paying attention to type of cereal. Moreover, consumption is likely to be guided by choice and availability through food subsidy or open-market access to other cereals and food staples, apart from those cultivated for subsistence. Our analysis indicates the need for NFHS and demographic health surveys worldwide to include type of cereal consumption to gain better understanding of pathways to malnutrition. The use of cereal cultivation as a proxy for consumption too is a source of noise in our data, as some of the cultivation is likely to be for non-human use (primarily fodder for animal use). Factors leading to lack of dietary diversity like poverty, prevalence of infections like worm infestations or tuberculosis and other possible unaccounted confounding factors could also be contributing to these patterns. The data on availability of nutrients from cereal consumption from nutritional assays (stable isotope-based) is also meagre to the best of our efforts in reviewing peer-reviewed evidence-base. Such data from cereal consumption could help in linking the dietary matrix to the effects described above.

## Conclusion

Higher wasting and stunting prevalence among children in India has an ecogeographic pattern with plausible links of pre-dominant millet consumption to higher prevalence of wasting. MUAC and type of cereal consumed should be incorporated in NFHS4 and all global demographic surveys and health surveys to enable better assessment of patterns of malnutrition. State of the art research in nutrient sensing should be integrated with agriculture, food science, delivery systems and dietary matrix for translational benefits to accrue to the wider population.

## Data availability

### Underlying data

Figshare: Dataset used to assess relationship between millet cultivation and malnutrition patterns in India at district level. https://doi.org/10.6084/m9.figshare.12236789.v2 ^26^

This project contains the following underlying data:

- malnutrition_dataset_for_publication.xlsx (Dataset used for analysis described in the paper)
- Malnutrition and millets – India – DACNET NFHS 4.docx (Word document explaining how the dataset was prepared)

### Extended data

Figshare: Plots examining relationship between type of millet cultivated with stunting and wasting at district level along with map showing the overlaps for each type of millet with stunting and wasting. https://doi.org/10.6084/m9.figshare.12206135.v4 ^105^

This project contains the following extended data:

• Malnutrition_millets and malnutrition.pdf (PDF file with panel of seven plots and maps, each showing relationship between type of millet cultivated with stunting and wasting at district level and the corresponding map showing the overlaps of each type of millet with stunting and wasting)

Figshare: Plots examining relationship between low BMI and short stature in women 15–49 with stunting and wasting at district level along with map showing the overlaps for each type of millet with low BMI and short stature in women (15–49). https://doi.org/10.6084/m9.figshare.12206264.v4

This project contains the following extended data:

- malnutrition_bmi_short_status.pdf (PDF file with panel of seven plots and maps, wach showing relationship between low BMI and short stature in women 15–49 with stunting and wasting at district level alogn with maps showing overlaps for each type of millet with low BMI and short stature in women (15–49))

Data are available under the terms of the Creative Commons Attribution 4.0 International license (CC-BY 4.0).

## Software availability

Source code available from: https://gitlab.com/asdofindia/malnutrition-crops-maps

Archived source code at time of publication: http://doi.org/10.5281/zenodo.3828725 ^33^

License: MIT license
