# Eco-geographic patterns of child malnutrition in India and its association with cereal cultivation: An analysis using demographic health survey and agriculture datasets

**DOI:** 10.12688/wellcomeopenres.15934.4

**Published:** 2022-02-22

**Authors:** Rama Krishna Sanjeev, Prashanth Nuggehalli Srinivas, Bindu Krishnan, Yogish Channa Basappa, Akshay S. Dinesh, Sabu K. Ulahannan

**Affiliations:** 1 Pediatrics, Rural Medical College, Pravara Institute of Medical Sciences, Loni (BK), Ahmednagar district, Maharashtra, 413736, India; 2 Health equity cluster, Institute of Public Health Bengaluru, Bengaluru, Karnataka, 560070, India; 3 Physiology, Rural Medical College, Pravara Institute of Medical Sciences, Loni (BK), Ahmednagar district, Maharashtra, 413736, India; 4 Independent researcher, Bengaluru, Karantaka, 560008, India

**Keywords:** Millets, malnutrition, wasting, stunting, MTORC1 (mechanistic target of Rapamycin complex1), GCN2 (general control non derepressible 2), DSCQ (District subsistence cultivation quantum)

## Abstract

**Background: **High prevalence of maternal malnutrition, low birth-weight and child malnutrition in India contribute substantially to the global malnutrition burden. Rural India has disproportionately higher levels of child malnutrition. Stunting and wasting are the primary determinants of child malnutrition and their district-level distribution shows clustering in different geographies and regions. Cereals, particularly millets, constitute the bulk of protein intake among the poor, especially in rural areas in India where high prevalence of wasting persists.

**Methods: **The previous round of National Family Health Survey (NFHS4) has disaggregated data by district, enabling a more fine-scale characterisation of the prevalence of markers of malnutrition. We used data from NFHS4 and agricultural statistics datasets to analyse relationship of prevalence of malnutrition at the district level and area under cereal cultivation. We analysed malnutrition through data on under-5 stunting and wasting by district.

**Results: **Stunting and wasting patterns across districts show a distinct geographical and age distribution; districts with higher wasting showed relatively higher prevalence at six months of age. Wasting prevalence at district level was associated with higher cultivation of millets, with a stronger association seen for jowar and other millets (Kodo millet, little millet, proso millet, barnyard millet and foxtail millet). District level stunting was associated with higher district level cultivation of wheat. In multivariable analysis, wasting was positively associated with women’s body mass index and stunting with women’s short stature.

**Conclusions: **Well-designed intervention studies will be required to confirm causal pathways contributing to ecogeographic patterns of child malnutrition. The cultivation of other millets has a strong association with prevalence of wasting. State-of-the-art studies that improve our understanding of bio-availability of amino acids and other nutrients from the prevalent dietary matrices of rural poor communities will be needed to confirm causal pathways contributing to potential eco-geographic patterns.

## Introduction

Undernutrition among children less than 5 years is measured by prevalence of stunting (height for age with z score of less than -2), wasting (weight for height with z score less than -2) and underweight (weight for age with z score less than -2). High prevalence of low birth weight (weight less than 2.5kg at birth), is also an important contributor to child undernutrition, and, forms a continuum to it within the first 1000 days ^1^. Low pre-pregnancy BMI, low maternal BMI (< 18.5 kg/m2), maternal short stature and maternal micronutrient deficiency or anemia all contribute to small for gestational age, low birth weight and prematurity ^2^. Out of the estimated 20.5 million babies born low birth weight annually, 48% are born in South Asia. India alone is estimated to have 100 million adult women with low BMI ^1, 2^. Globally, the World Health Organization (WHO) estimates that among children under five, about 151 million suffer from stunting and 51 million from wasting with consequent risks of mortality, morbidity and delayed development ^3^. The latest stunting trends indicate increases in Africa along with substantial reductions across Asia. However, with regards to wasting, with a regional prevalence of 12%, South Asia accounts for half of all wasted children globally ^1, 4, 5^. India reports 21 % wasting of children under 5 years numbering about 27 million ^4^. South Asia is also estimated to have ~45% of the global burden of stunting. The socio-economic gains and poverty reduction of the past decades have not translated into commensurate reduction of stunting and wasting in children, often characterised as, *the Asian enigma* ^6– 8^.

### Subsistence farming and millet dependence

Indian states consist of 640 districts (at the time of NFHS4) with wide differences in geography, climate and the main agricultural crops. India has a large and poor rural population (68.9% rural with 25.5 % rural poverty prevalence), and over half (54%) of the working rural population (481.9 million) are cultivators and agricultural labourers ^9, 10^. Small land-holding farmers (owning less than two hectares of land) and their families constitute more than half the country’s population. Only half (96.46 million hectares) of the total area under cultivation (198.36 million hectares) is irrigated ^11^. Although, rice and wheat together constitute 75% of total area under food grain cultivation, Jowar (Sorghum) and Bajra (pearl millet) make up a significant 13.8%. However, the distribution of food grain cultivation in irrigated land varies, with rice (60%) and wheat (94.2%), expectedly being grown largely on irrigated land. In contrast, Sorghum (Jowar) and Pearl millet (Bajra) are grown largely in non-irrigated lands, most likely by small land-holding farmers in monsoon-dependent arid or semi-arid regions of the country, which are also among the poorest ^12, 13^. Cereal cultivation and consequently household food grain consumption and diets in such regions are likely driven by these strong linkages between agro-climatic, edaphic, and eco-geographic factors, more so among poorer households with socio-economic barriers to achieve dietary diversity.

A study based on National Family Health Survey-3, which reported results at the state level for India in 2005–6, demonstrated considerable geographic variation among the states of India with regards to child malnutrition, with higher levels of stunting seen in Uttar Pradesh, Uttaranchal & Gujarat ^14^. In contrast, higher wasting levels were seen in Madhya Pradesh, a state in central India. A nutritional survey among preschool children in three tribal regions belonging to different ecological zones in the state of Madhya Pradesh, India, namely Jhabua, Bastar and Sarguja, showed greater extent and severity of malnutrition among children in Jhabua. The staple cereals reported in the study for Jhabua was maize and Sorghum, while for Bastar and Sarguja, it was rice ^15^. Sorghum, as staple, has also been linked to endemic pellagra among farm workers in Hyderabad by Gopalan ^16^. Subsistence crop cultivation has been linked to seasonal "epidemic" nutritional edema among American farmers in the 1930s ^17, 18^. Much earlier,at the beginning of last century, nutritional edema among children weaned on a diet of cereal flour was called *Mehlnahrschden* or flour dystrophy in Germany ^17^. Cecily Williams in her classic description of Kwashiorkor attributed it to weaning on a pre-dominantly maize-based staple ^19^.

The Lancet 2008 series too has brought out this aforementioned pattern of child malnutrition, with areas having similar prevalence of stunting demonstrating substantial differences in wasting ^20^. Likewise, low women’s BMI (15–49 years age) too, has numerous geographical subnational hotspots in South Asia ^1^.

Geo-spatial heterogeneity in prevalence of child malnutrition across Indian districts has been reported ^21^. The NFHS 4 was conducted in 2015–16 incorporating district-level data for the first time ^22^. Based on unpublished field observations of wasting prevalence among populations depending on millet as staple in rural Maharashtra (spanning western and central India), we critically examined the spatial patterns of prevalence of stunting and wasting at the district level across India with the objective of exploring the role of dietary staple cereal consumption pattern using cultivation pattern as a proxy. Ragi (finger millet; *Eleusine coracana*) was excluded because it belongs to a distinct sub-family in the grass family Poaceae and has a relatively better nutritional profile ^23– 25^.

## Methods

We analysed district-level secondary data on under-5 stunting and wasting as reported in NFHS4 with district-wise crop cultivation data to assess geo-spatial overlaps and risk relationships between pre-school child malnutrition and cultivation of staple cereal crops. NFHS is a standardised and periodic nationally representative survey. NFHS4 covered 601,509 households, 699,686 women aged 15–49 years and 103,525 men aged 15–54 years that provides comprehensive data on various aspects of maternal and child health ^21, 26^. NFHS-4 provides unit level data (for each of the 640 districts of India at the time of survey) for download upon request via the demographic health survey data repository ^26, 27^. We extracted data on population of each district from the 2011 Census ^10^. We included other socio-demographic variables with known associations with malnutrition from NFHS4 to assess their relative contribution to childhood wasting and stunting, at district level using multivariable linear regression.

### Definitions and data sources

We considered the following cereal crops widely grown and reported in Indian agriculture databases and the Directorate of Millets Development (under Department of Agriculture, Co-operation and Farmers Welfare) in our analysis: rice( *Oryza sativa*), wheat( *Triticum aestivum*), maize( *Zea mays*), jowar (sorghum; *Sorghum bicolor*), bajra (pearl millet; *Pennisetum glaucum*) and other millets (kodo millet: *Paspalum scrobiculatum*, little millet: *Panicum sumatrense*, proso millet: *P. miliaceum*, barnyard millet: *Echinochloa esculenta* and foxtail millet: *Setaria italica*). Henceforth, millets in the text means Jowar, Bajra and other millets combined.

We adopted the definitions of districts with high prevalence of wasting and stunting from district-level malnutrition analysis by Junaid and Mohanty ^21^, which has considered >46% district-level stunting prevalence (Z score **≤** -2), and >28% district-level wasting prevalence (Z score **≤** -2) as representing high prevalence districts for stunting and wasting respectively.

We extracted variables of interest from NFHS4 (see variables listed below). For data on cultivation of cereal crops, we used DACNET, a web-based land use statistics information system maintained by the Agriculture Informatics Division of the National Informatics Centre of the Government of India ^28^.

The following data were extracted to prepare a district-level dataset for analysis ^29^:

1. From the 2011 census data, district-wise total and rural population
2. From the NFHS4 data,
  a. using appropriate weights BMI less than 18.5 and short stature less than 145cm of women aged 15–49 years, utilization of Anganwadi, dietary diversity (age 6–23months), women with 10years or more of education, household wealth quintiles (lowest and second), open defecation and rural population.
  b. district-level percentage of wasting and stunting was calculated from the children dataset
  c. percentage of people in household wealth quintiles, open defecation for a given district was calculated from household dataset
3. Various crop data is available in state-wise reports compiled by the Ministry of Agriculture and Farmers Welfare. We extracted district-level area under cultivation of cereals: rice, wheat, maize, ragi, bajra, jowar, and millets (by type as defined above) into a spreadsheet. Data was from the latest state-wise reports available at the time of analysis at DACNET ^28^ (data for most states ranged for years between 2014–17 except Maharashtra 2002–03, Manipur 2004–05 and Gujarat 2007–08; all data in hectares converted to acres).

Using district names as the common variable in all three datasets, the variables from these three datasets were merged into a single dataset ^29^. Any errors due to district spellings and duplicate district names across differing states were handled with caution to ensure proper merging. For each district we estimated the population of poor by multiplying the census figures for population of the district by the proportion of the population in the fourth and fifth wealth quintiles (from NFHS4). This was based on the assumption that subsistence cereal consumption is largely restricted to poor small land-holding farmers ^30, 31^. Since, Sorghum and other millets are largely cultivated by poor farmers with small land holdings for subsistence purposes with the exception of economically better-off and well-irrigated regions, particularly in northern India ^31– 33^. District Subsistence Cultivation Quantum (DSCQ) for each district was obtained by multiplying the per capita area (cereal cultivation area in acres/total population) by the proportion of the poor (in the lowest two wealth quintiles as per NFHS) followed by normalising data using logarithmic transformation. The independent and dependent variables used in scatter plots, bar charts as well as bivariate and multi variable analysis have been enumerated in Table 1.

**Table 1.** Variables (independent & dependent) used in different analysis.

S.NO	Type of analysis	Independent variable	Dependent variable
1	Maps in Figure 1– Figure 6	DSCQ # of cereals-rice, wheat, jowar, bajra, other millets	High stunting districts (>46%) High wasting districts (>28%)
2	Bivariate analysis (scatter plots) in Figure 2– Figure 6	DSCQ # (normalized using log transformation) of cereals-rice, wheat, jowar, bajra, other millets	Prevalence of stunting and wasting percentage by district
3	Multivariable regression Table 3– Table 4	I. BMI less than 18.5 II. short stature less than 145cm of women aged 15–49 years III. utilization of Anganwadi IV. Dietary diversity (age 6–23months) V. women with 10years or more of education VI. Household wealth quintiles(lowest and second lowest) Vii. open defecation Viii. rural population. IX. Log of district wise area of crops (rice, wheat, jowar, bajra and other millets) in hectares with 1 added as a constant Above variables were controlled for in multivariable regression for prevalence of under 5 stunting and wasting in Table 3 & Table 4	Prevalence of stunting and wasting percentage by district
4	Bar charts Figure 9	Cultivation area in hectare of cereal crops rice, wheat, jowar, bajra, other millets	Percentage of stunting/wasting children under five years of age

#: DSCQ- District Subsistence Cultivation Quantum, calculated for each district by multiplying Per capita Cereal area (Cereal area in acres / Total population of district) with proportion of the poor( in the lowest two wealth quintiles of the district as per NFHS)

### Analysis

***Spatial malnutrition patterns.*** We assessed overlaps between high prevalence of stunting and/or wasting with cereal cultivation data by generating maps derived from The Database of Global Administrative Areas (GADM) ^34^. We merged tabular data (from a spreadsheet file) with geographic data (from a geojson file), chose variables of interest, created map legends dynamically and rendered multiple maps using a custom-built wrapper software written in javascript which internally uses Mapbox GL JS library (version 1.10.0) for rendering maps ^35^. Further information on what this software wrapper does and how it works is present in the README file of the source code ^36^. As a base layer, DSCQ was shaded using a linear interpolator with manually chosen colour levels for the legend. A transparent layer of outcome variables (stunting and wasting) marked with distinct stripe patterns was overlaid on the base layer for visualizing overlap.

***Examining relationship between subsistence millet cultivation, childhood malnutrition and its early onset.*** For each cereal, we examined its association with district-level prevalence of stunting and wasting and DSCQ (normalised using logarithmic transformation) by linear regression. We also examined the relationship of age with wasting and stunting at the district level by plotting the prevalence percentages by age, from 6 months onwards till 5 years, in both groups of districts, with high prevalence of stunting and wasting. For multi-variable regression, since cereal cultivation distribution had high variability and was skewed, the logarithm of cultivation area in hectares (with 1 added as a constant ), was taken for analysis. For both women 10 or more years of education and toilet facilities, we categorized into binary 1 & 0, with 1 standing for 10 or more years of education and presence of toilet facilities, respectively. For utilization of Anganwadi, the variable was constructed from benefits accrued from Anganwadi centre and frequency of food received during the last 12 months. The information was aggregated at district levels with appropriate sample weights. For dietary diversity this was calculated as per the guide DHS program data guide for dietary diversity.

## Results

**Figure 1.**
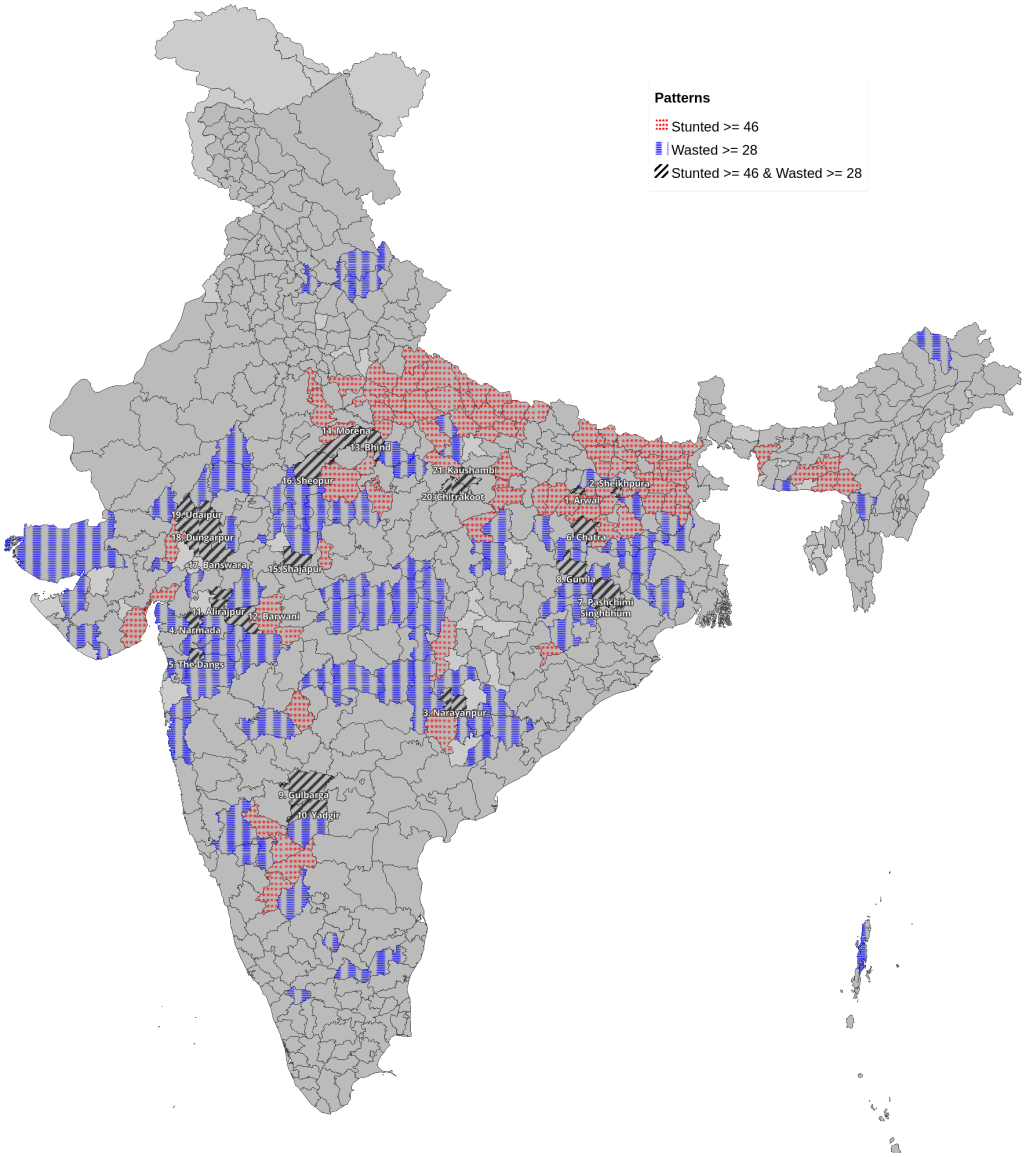
Map of India showing areas with higher prevalence of stunting (>46%) in rows of red dots and those with higher prevalence of wasting (>28%) in columns of blue bars. Districts with higher prevalence of both stunting and wasting are numbered cross referenced to Table 2 and marked with oblique bars.

In all, 107 districts had a high prevalence of stunting (ranging from 46–65% district prevalence) with risk concentrated in poorer states: Uttar Pradesh (31; 29%) Bihar (28; 26%) and Madhya Pradesh (13; 12%) (numbers in brackets are number of districts followed by percentage). Among the 112 districts, those with higher rates of wasting (ranging from 28–47% district prevalence) were in districts with pre-dominantly tribal population in Jharkhand (14; 12.5%), Madhya Pradesh (20; 17.8%), Maharashtra (12; 11%), Rajasthan (11; 9.8%) and Gujarat (10; 9%) (numbers in brackets are number of districts followed by percentage). High stunting areas were concentrated in north and eastern India, whereas areas of high wasting were primarily in central India, which had high prevalence of both childhood stunting and wasting ( Figure 1). There were 21 districts with high levels of both stunting and wasting, of which 16 had millets of any type or maize as either the most dominant (n=6) or second most dominant crop (n=10) ( Table 2). Of these 21 districts, there were three from Rajasthan, which had more maize cultivation than any other cereal crop: Udaipur (62 %), Banswara (51%), and Dungarpur (47%).

**Table 2.** Districts with high prevalence of stunting(>46%) and high prevalence of wasting(>28%) as per the integrated dataset from NFHS4 and agriculture statistics ^29^.

S.No	District	State	Major crop1	Percentage	Major crop 2	Percentage	Major crop 3	Percentage
1	Arwal	Bihar	Rice	73.64	Wheat	25.21	Maize	0.9
2	Sheikhpura	Bihar	Wheat	49.37	Rice	49.12	Maize	1.5
3	Narayanpur	Chhatisgarh	Rice	87.17	Other millets	7.66	Maize	3.8
4	Narmada	Gujarat	Rice	59.38	Jowar	27.07	Wheat	7.86
5	The Dangs	Gujarat	Rice	64.86	Other millets	16.98	Jowar	16.60
6	Chatra	Jharkhand	Rice	78.44	Maize	11.7	Wheat	8.99
7	Pashchimi Singhbhum	Jharkhand	Rice	98.89	wheat	0.61	Maize	0.47
8	Gumla	Jharkhand	Rice	88.18	Ragi	6.5	Other millets	2.4
9	Gulbarga	Karnataka	Jowar	86.11	Bajra	5.10	Wheat	3.89
10	Yadgir	Karnataka	Rice	62.26	Jowar	24.99	Bajra	12.35
11	Alirajpur	Madhya Pradesh	Maize	42.4	Wheat	21.99	Bajra	14.42
12	Barwani	Madhya Pradesh	Maize	35.02	Wheat	33.77	Jowar	26.04
13	Bhind	Madhya Pradesh	Wheat	67.57	Bajra	22.00	Jowar	4.06
14	Morena	Madhya Pradesh	Wheat	50.46	Bajra	48.11	Rice	0.44
15	Shajapur	Madhya Pradesh	Wheat	99.44	Jowar	0.53	Rice	0.02
16	Sheopur	Madhya Pradesh	Wheat	66.24	Rice	21.57	Bajra	10.58
17	Banswara	Rajasthan	Maize	51.63	Wheat	34.42	Rice	12.17
18	Dungarpur	Rajasthan	Maize	47.50	Wheat	35.06	Rice	13.19
19	Udaipur	Rajasthan	Maize	62.65	Wheat	29.25	Jowar	2.62
20	Chitrakoot	Uttar Pradesh	Wheat	62.43	Bajra	13.36	Rice	11.23
21	Kaushambi	Uttar Pradesh	Wheat	53.69	Rice	35.95	Bajra	6.33

**Figure 2.**
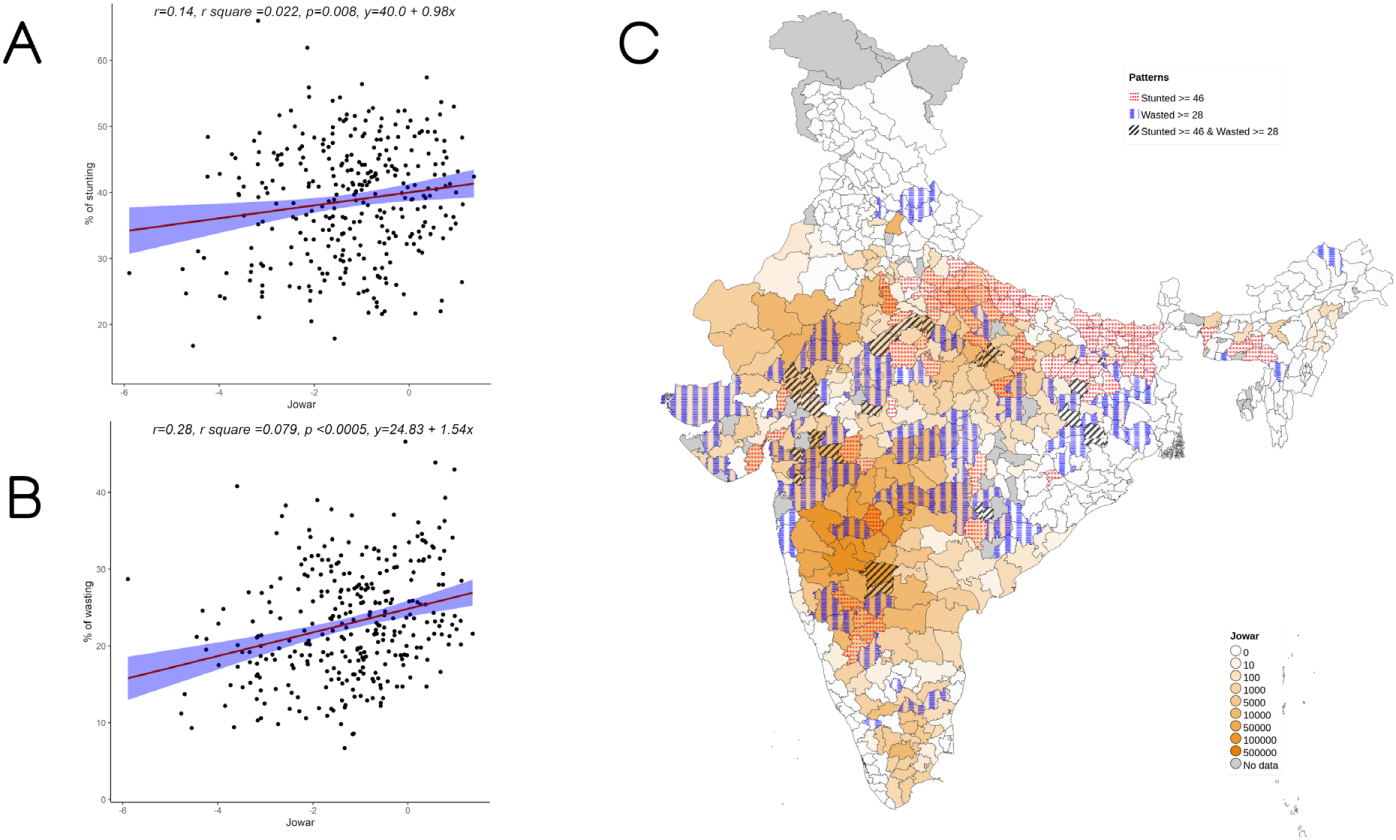
Plots examining relationship between jowar cultivated with stunting and wasting at district level along with map showing the overlap of jowar cultivated with stunting and wasting. **A**) Scatterplot of stunting v/s district subsistence cultivation quantum (DSCQ) of jowar by poor **B**) Scatterplot of wasting v/s DSCQ of jowar by poor **C**) Geographic distribution of DSCQ of jowar by poor, stunting > 46 & wasting >28.

**Figure 3.**
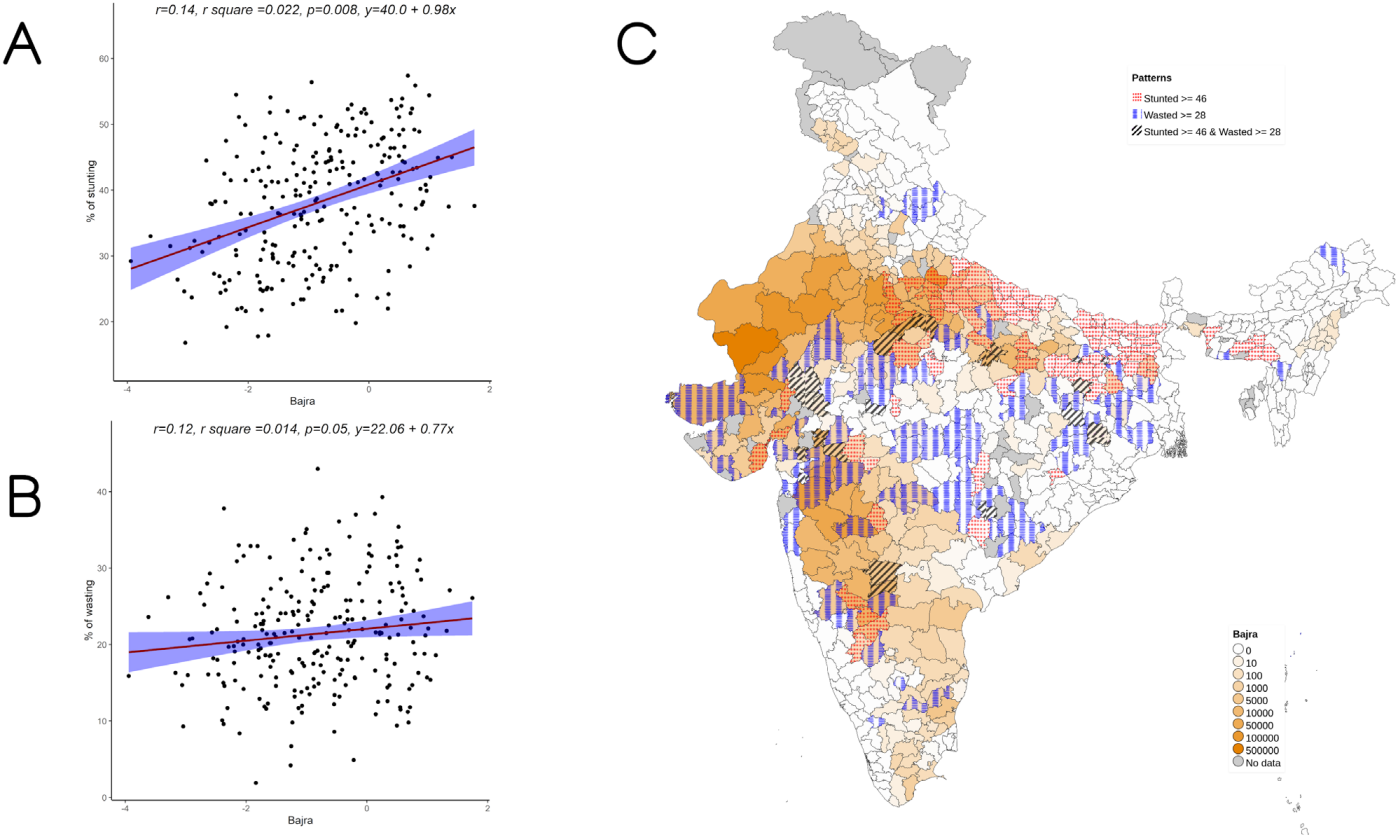
Plots examining relationship between bajra cultivated with stunting and wasting at district level along with map showing the overlap of bajra cultivated with stunting and wasting. **A**) Scatterplot of stunting v/s DSCQ of bajra by poor **B**) Scatterplot of wasting v/s DSCQ of bajra by poor **C**) Geographic distribution of DSCQ of bajra by poor, stunting > 46 & wasting >28.

On examining the district-level patterns of subsistence cultivation of jowar by district overlaid over districts having higher prevalence of stunting and wasting, we find that there is an overlap of districts with wasting alone and those with stunting and wasting with higher DSCQ for jowar ( Figure 2). Maps of Bajra show areas with higher DSCQ, particularly in parts of Northern and Western India, with no high stunting or wasting prevalence. Similar maps, separately showing overlap of high stunting and high wasting with per-capita cultivation of jowar, wheat, rice, bajra, maize, and other millets are also available ^36^. There is an overlap of districts with high wheat and rice cultivation in the well irrigated Gangetic plains (North and Eastern parts) with stunting ( Figure 5 & Figure 6). Cultivation of other millets is scattered throughout the country with an overlap with high prevalence of wasting. The large, irrigated areas in the Northwest & Central India with high DSCQ of Bajra & Jowar also have higher DSCQ of rice and wheat as seen in Figure 2, Figure 3, Figure 5 & Figure 6. 

Overall, increase in cultivation of jowar, bajra and other millets is independently associated with increase in prevalence of both stunting and wasting (see Figure 3 – Figure 5). When the association was examined for individual millets, whereas jowar cultivation did show an association with increase in both stunting and wasting, increase in bajra cultivation was associated only with increase in stunting. Increase in cultivation of other millets was associated with increase in wasting only (a reverse trend was seen with stunting). As expected, there was either no change or decrease seen when we examined association between increase in rice or wheat cultivation with wasting (with an increase in stunting associated with increase in rice or wheat cultivation).

**Figure 4.**
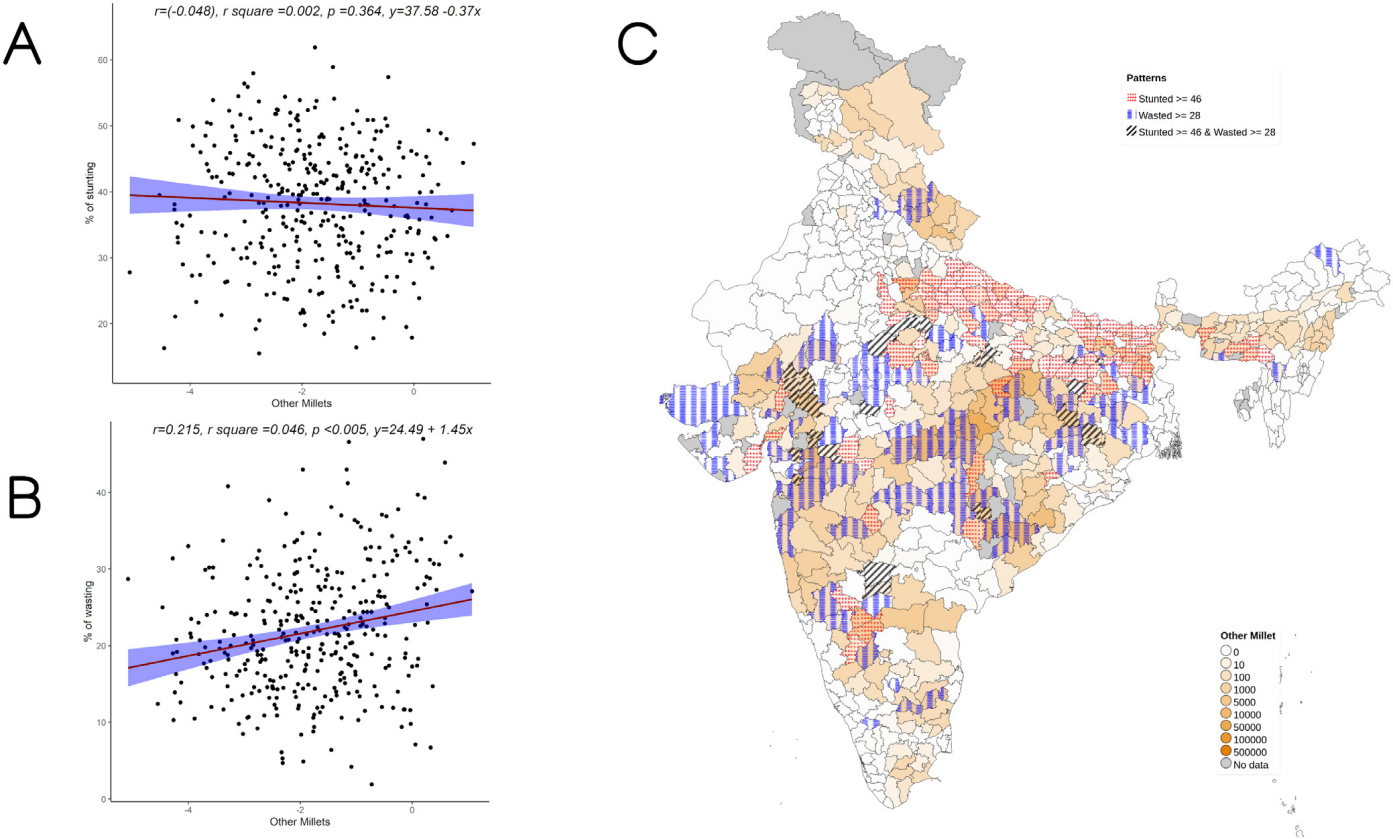
Plots examining relationship between Other millets (kodo millet, little millet, proso millet, barnyard millet and foxtail millet) cultivated with stunting and wasting at district level along with map showing the overlap of Other millets cultivated with stunting and wasting. **A**) Scatterplot of stunting v/s DSCQ of other millets by poor **B**) Scatterplot of wasting v/s DSCQ of Other millets by poor **C**) Geographic distribution of DSCQ of Other millets by poor, stunting > 46 & wasting >28.

**Figure 5.**
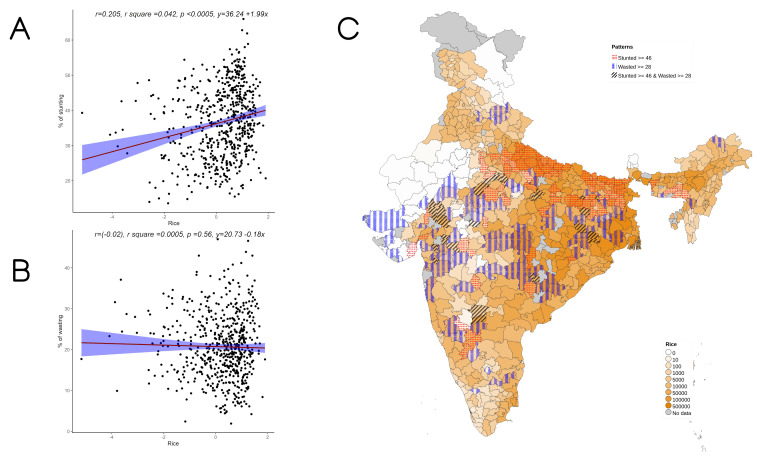
Plots examining relationship between rice cultivated with stunting and wasting at district level along with map showing the overlap of rice cultivated with stunting and wasting. **A**) Scatterplot of stunting v/s DSCQ of rice by poor **B**) Scatterplot of wasting v/s DSCQ of rice by poor **C**) Geographic distribution of DSCQ of rice by poor, stunting > 46 & wasting >28.

**Figure 6.**
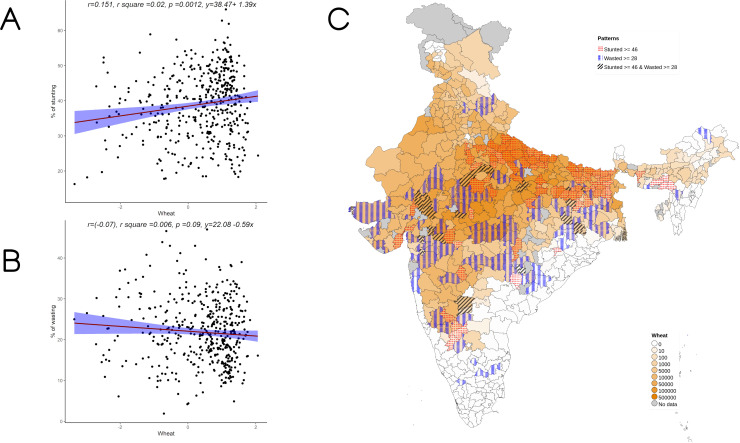
Plots examining relationship between wheat cultivated with stunting and wasting at district level along with map showing the overlap of wheat cultivated with stunting and wasting. **A**) Scatterplot of stunting v/s DSCQ of wheat by poor **B**) Scatterplot of wasting v/s DSCQ of wheat by poor **C**) Geographic distribution of DSCQ of wheat by poor, stunting > 46 & wasting >28.

**Figure 7.**
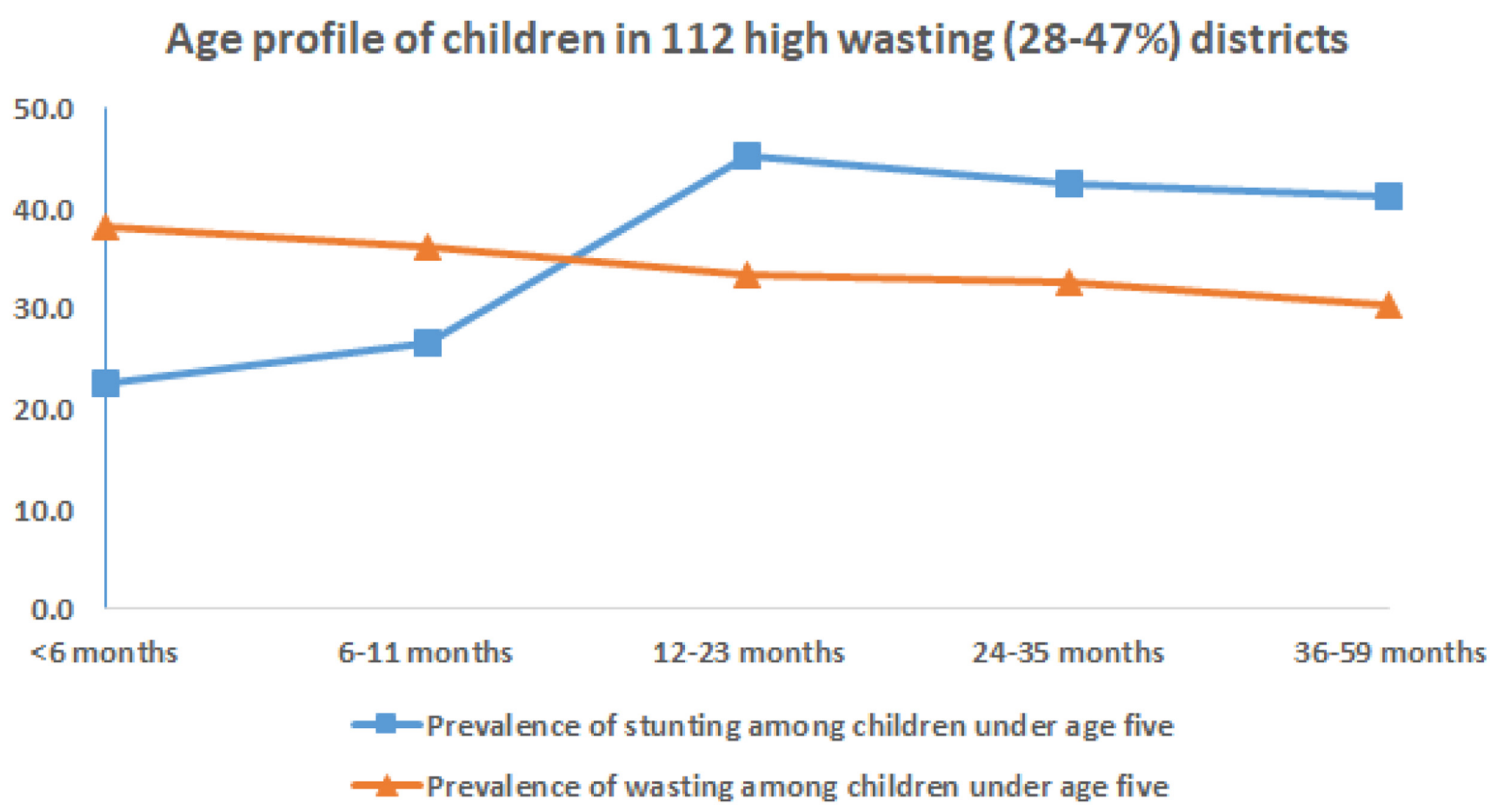
Age profile of stunted and wasted children in 108 high wasting (28–47%) districts.

**Figure 8.**
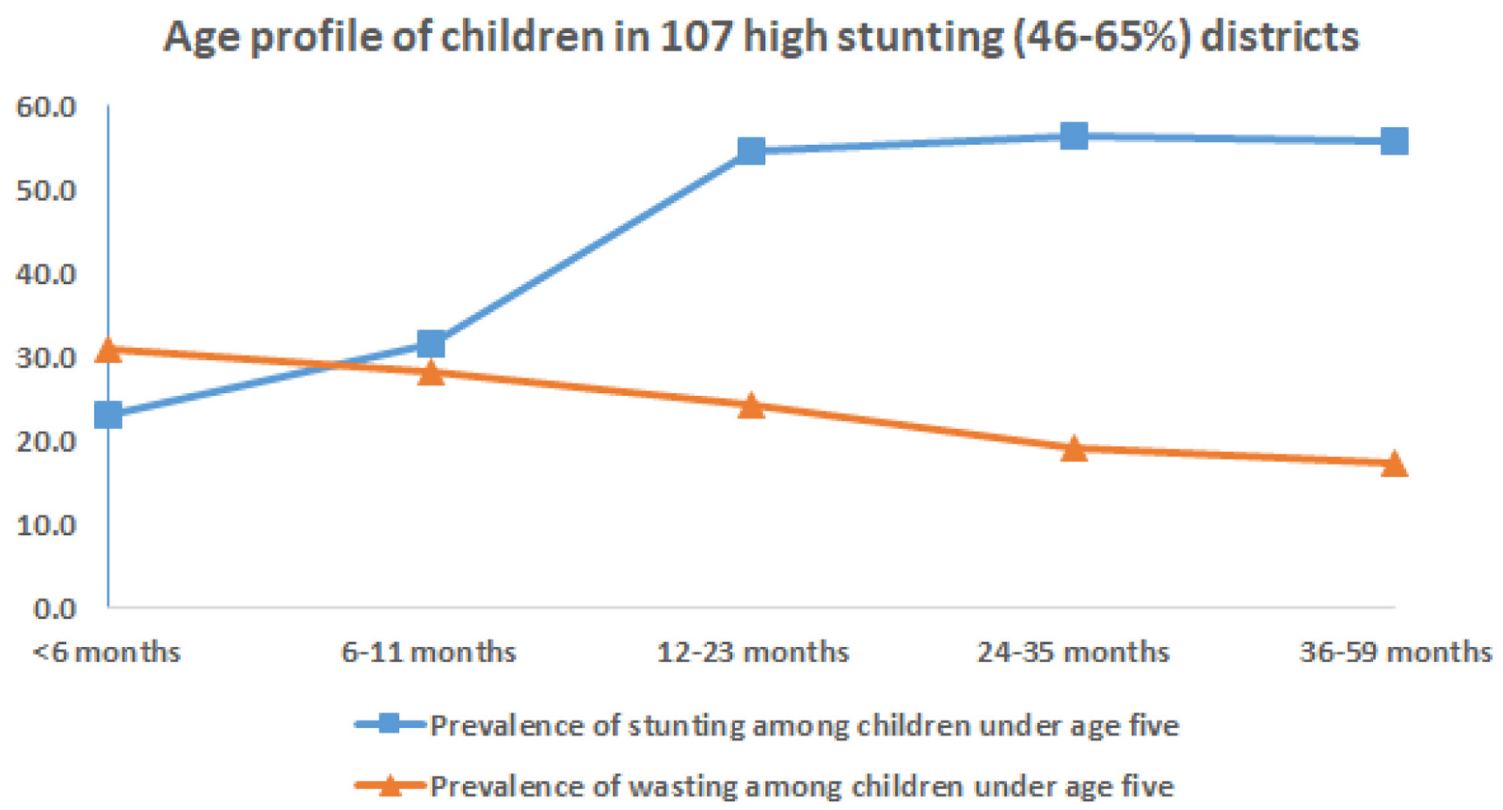
Age profile of stunted and wasted children in 112 high stunting (46–67%) districts.

On examining the age of children in districts with higher prevalence of stunting and wasting the following observations are evident, as seen in Figure 7 & Figure 8. In 112 districts with high wasting, wasting showed an early onset with highest wasting (40%) at 6 months of age ( Figure 7). The age-distribution of stunting was similar for both groups of districts with highest age-specific stunting prevalence at 12 months and a plateau thereafter till five years of age ( Figure 7 & Figure 8).

In multiple linear regression the analysis was controlled for confounders which included poor (calculated as belonging to the lower two quintiles of the wealth index), women =>10 years of education, proportion of rural, Open defecation, minimum dietary diversity, utilization of anganwadi, women’s short stature (<145 cms) in 15–49 years of age, women’s BMI less than 18.5 in the 15–49 years of age group, cultivation of Jowar, Bajra, other millets, rice and ragi and the outcome of interest is under 5 wasting ( Table 3) or under 5 stunting ( Table 4). In under 5 wasting, statistically significant negative association was seen with proportion of rural, minimum dietary diversity, bajra cultivation and a positive association was seen with women’s BMI less than 18.5 as well as open defecation. The cultivation of jowar and other millets was significantly associated positively with wasting, which was consistent with the results of the bivariate analysis seen in Figure 2 & Figure 4 (with r values of jowar & other millets being 0.28 and 0.215 respectively). The R square for the multivariable analysis for under 5 wasting as per model 7 was 0.382 implying that 38% of variance in wasting was attributable to the analyzed factors.

**Table 3.** Multivariable Regression models exploring the association between poor, women =>10 years of education, Proportion of rural, Open defecation, Minimum dietary diversity, Utilization of anganwadi, Women’s short stature (<145 cms) in 15–49 years of age, Women’s BMI less than 18.5 in the 15–49 years of age, cultivation of Jowar, Bajra, other millets, rice and ragi and the outcome of interest is % of under 5 wasting.

Variables	Model-1	Model-2	Model-3	Model-4	Model-5	Model-6	Model-7
	Unadjusted coefficients (95% CI)	Adjusted coefficients (95% CI)	Adjusted coefficients (95% CI)	Adjusted coefficients (95% CI)	Adjusted coefficients (95% CI)	Adjusted coefficients (95% CI)	Adjusted coefficients (95% CI)
Poor	0.073 ***	-0.029				0.005	-0.025
	(0.050 - 0.095)	(-0.072 - 0.014)				(-0.036 - 0.047)	(-0.072 - 0.022)
women =>10 years of education	-0.074 ***	-0.007				0.016	0.016
	(-0.105 - -0.042)	(-0.049 - 0.035)				(-0.026 - 0.058)	(-0.027 - 0.058)
Proportion of rural	0.030 *	-0.042 *				-0.075 ***	-0.058 ***
	(0.002 - 0.058)	(-0.075 - -0.008)				(-0.107 - -0.042)	(-0.092 - -0.025)
Open defecation	0.125 ***	0.157 ***				0.120 ***	0.070 ***
	(0.106 - 0.145)	(0.129 - 0.185)				(0.092 - 0.148)	(0.039 - 0.101)
Minimum dietary diversity	-0.168 ***		-0.173 ***			-0.119 ***	-0.056 **
	(-0.206 - -0.129)		(-0.210 - -0.136)			(-0.157 - -0.080)	(-0.099 - -0.014)
Utilization of anganwadi	0.093 ***		0.098 ***			0.088 ***	0.046 **
	(0.064 - 0.122)		(0.070 - 0.125)			(0.061 - 0.115)	(0.016 - 0.075)
Women short stature	0.026						
	(-0.073 - 0.125)						
Women BMI less than 18.5	0.372 ***			0.372 ***			0.211 ***
	(0.321 - 0.422)			(0.321 - 0.422)			(0.135 - 0.287)
Jowar	0.620 ***				0.670 ***		0.326 ***
	(0.482 - 0.757)				(0.500 - 0.840)		(0.163 - 0.488)
Bajra	0.228 **				-0.306 ***		-0.194 *
	(0.085 - 0.371)				(-0.481 - -0.131)		(-0.366 - -0.022)
Wheat	0.363 ***				0.365 ***		0.135
	(0.243 - 0.483)				(0.243 - 0.488)		(-0.003 - 0.272)
Other millets	6.689 ***				4.299 ***		3.372 ***
	(4.472 - 8.905)				(2.185 - 6.412)		(1.404 - 5.341)
Rice	0.052						
	(-0.128 - 0.233)						
Ragi	0.490 ***				0.488 ***		0.237 *
	(0.296 - 0.684)				(0.303 - 0.674)		(0.057 - 0.418)
Observations	640	640	640	640	640	640	640
R-squared		0.218	0.167	0.247	0.221	0.299	0.382

*** p<0.001, ** p<0.01, * p<0.05

**Table 4.** Multivariable Regression models exploring the association between poor, women =>10 years of education, Proportion of rural, Open defecation, Minimum dietary diversity, Utilization of anganwadi, Women short stature (<145 cms) in 15–49 years age, Women’s BMI less than 18.5, cultivation of Jowar, Bajra, other millets, rice and ragi and the outcome of interest is % of under 5 stunting.

Variables	Model-1	Model-2	Model-3	Model-4	Model-5	Model-6	Model-7
	Unadjusted coefficients (95% CI)	Adjusted coefficients (95% CI)	Adjusted coefficients (95% CI)	Adjusted coefficients (95% CI)	Adjusted coefficients (95% CI)	Adjusted coefficients (95% CI)	Adjusted coefficients (95% CI)
Poor	0.248 ***	0.052 *				0.062 **	**0.031**
	**(0.225 - 0.271)**	**(0.010 - 0.093)**				**(0.023 - 0.102)**	**(-0.014 - 0.076)**
women =>10 years of education	-0.335 ***	-0.197 ***				-0.137 ***	-0.104 ***
	**(-0.367 - -0.304)**	**(-0.237 - -0.156)**				**(-0.177 - -0.097)**	**(-0.143 - -0.065)**
Proportion of rural	0.164 ***	-0.044 **				**-0.030**	**-0.012**
	**(0.130 - 0.198)**	**(-0.077 - -0.012)**				**(-0.061 - 0.001)**	**(-0.043 - 0.019)**
Open defecation	0.235 ***	0.146 ***				0.132 ***	0.086 ***
	**(0.214 - 0.257)**	**(0.119 - 0.173)**				**(0.106 - 0.159)**	**(0.057 - 0.114)**
Minimum dietary diversity	-0.339 ***		-0.336 ***			-0.149 ***	-0.082 ***
	**(-0.384 - -0.294)**		**(-0.381 - -0.292)**			**(-0.186 - -0.113)**	**(-0.121 - -0.043)**
Utilization of anganwadi	-0.058 **		-0.048 **			-0.068 ***	-0.038 **
	**(-0.096 - -0.019)**		**(-0.081 - -0.015)**			**(-0.093 - -0.042)**	**(-0.065 - -0.011)**
Women short stature	0.902 ***			0.612 ***			0.474 ***
	**(0.796 - 1.007)**			**(0.512 - 0.712)**			**(0.376 - 0.571)**
Women BMI less than 18.5	0.571 ***			0.427 ***			**0.065**
	**(0.511 - 0.631)**			**(0.368 - 0.487)**			**(-0.005 - 0.134)**
Jowar	0.511 ***				0.265 *		**0.079**
	**(0.328 - 0.694)**				**(0.053 - 0.476)**		**(-0.072 - 0.230)**
Bajra	0.467 ***				**-0.052**		**0.146**
	**(0.285 - 0.648)**				**(-0.274 - 0.170)**		**(-0.011 - 0.304)**
Wheat	0.964 ***				0.901 ***		0.315 ***
	**(0.825 - 1.103)**				**(0.749 - 1.052)**		**(0.191 - 0.439)**
Other millets	**2.039**						
	**(-0.875 - 4.954)**						
Rice	0.420 ***				0.361 ***		**-0.102**
	**(0.191 - 0.650)**				**(0.153 - 0.570)**		**(-0.258 - 0.055)**
Ragi	**-0.222**						
	**(-0.475 - 0.030)**						
Observations	**640**	**640**	**640**	**640**	**640**	**640**	**640**
R-squared		**0.557**	**0.266**	**0.473**	**0.246**	**0.617**	**0.684**

*** p<0.001, ** p<0.01, * p<0.05

For stunting a significant negative association was seen with women’s education of more than 10 years and minimum dietary diversity. A significant positive association was seen with open defecation & women’s short stature. Among the crops a positive association was seen with wheat cultivation similar to that seen in bivariate analysis in Figure 6. with an r of 0.151. The R square of multivariable analysis as per model 7 was 0.684 implying that 68% of the variance in stunting was explained by the analyzed variables.

**Figure 9.**
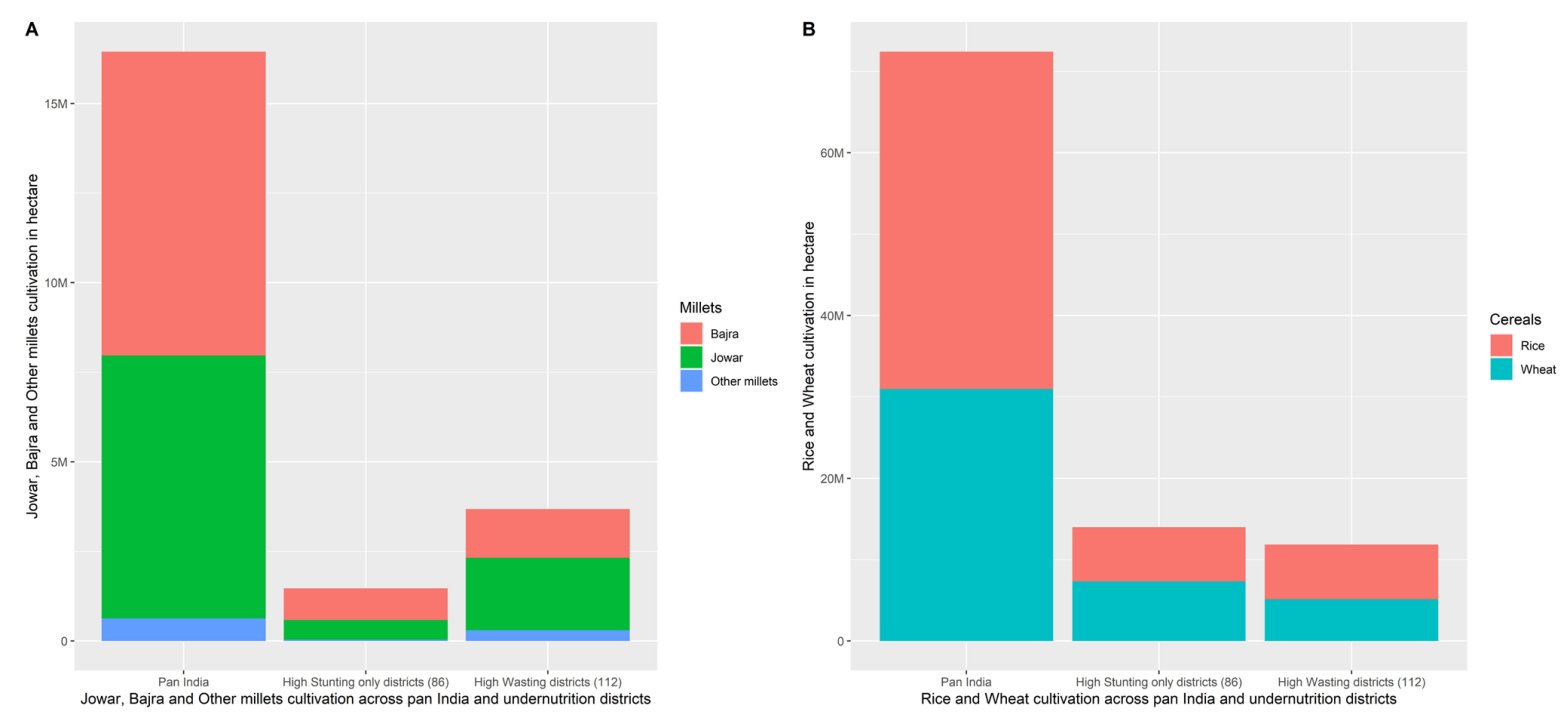
**A**. Cultivation of Jowar, Bajra and Other millets all over India, high stunting (only) districts and high wasting districts. **B**. Cultivation of rice and wheat all over India, high stunting (only) districts and high wasting districts.

In Figure 9, the area of cereal cultivation among all 640 districts, high stunting only (86) districts and high wasting (112) districts are shown. Figure 9A shows substantially higher cultivation of Jowar, Bajra and other millets in the high wasting districts in comparison to high stunting (only) districts. See contrast in Figure 9B where the cultivated area with respect to rice and wheat indicates greater cultivation of rice and wheat in the 86 high stunting only districts in comparison to the 112 high wasting districts.

## Discussion

The terms stunting and wasting were introduced by John Waterlow in the 1970s to differentiate underweight children with low weight for height, which constitutes wasting and those with low height for age, implying stunting ^37^. Stunting and wasting differ in terms of body composition with greater loss in muscle mass and fat in the latter. Adiposity also indirectly affects stature; periods of wasting are followed a few months later by stunting in the same individual, probably mediated by Leptin ^38^. The phenomenon of both stunting and wasting together has been named concurrent WaSt ^39^. However, most stunting is unrelated to wasting as several populations have high prevalence of stunting in the absence of previous wasting ^5^. Gain in height requires skeletal and lean body mass growth with need for additional resources including micronutrients such as Calcium, Magnesium, Phosphorus, Sulphur, Copper and Vitamins C, D and K ^38, 40^. Absence of the above micronutrients and vitamins can cause children to become stunted with or without adiposity depending on provision of other nutrients ^40^. In either wasting or stunting, children are at risk of higher mortality with highest risk being those having both together ^38^.

### Age and geographical patterns of stunting and wasting

On seeing the age profile of children with wasting and stunting ( Figure 7 and Figure 8) in the 112 high wasting prevalence and 107 high stunting prevalence districts, wasting at 6 months is higher (40% prevalence) in the high wasting districts, and lower (30% prevalence) in the latter. The mean prevalence of wasting at 6 months for the 640 districts of the country as per the NFHS4 dataset was 31.9%. Prevalence of stunting at 6 months was 20% in both groups of districts which was similar to the national prevalence ^22^.

A study analysing severe wasting among Indian infants less than 6 months of age using NFHS 4 dataset showed highest prevalence of severe wasting in the relatively prosperous Maharashtra and Gujarat (over 20%), in comparison to less than 15% prevalence in Uttar Pradesh and Bihar which are poorer ^41, 42^. In a multi-country study utilizing 20 demographic and health survey datasets, wasting under 6 months of age was highest in India (30.5% according to NFHS3 data) followed by Burkina Faso (25%), Nigeria (24.1%), Niger (19.7%) and Mali (18.2%) ^43^. Like the states of Maharashtra and Gujarat for India, the above countries too are among the highest millet producing regions in the world ^44, 45^. We examined and verified this pattern using a composite dataset with FAOSTAT data on agricultural production and prevalence of WaSt, stunting & wasting and wasting at 6 months ^45^.

Martorell *et al*. ^46^ compared differences in age patterns of stunting and wasting between India and Guatemala, utilizing NFHS 3 and Reproductive health survey 2008-9 respectively. India had much higher levels of wasting and similar levels of stunting with respect to Guatemala ^46^. This comparison was triggered by the use of WHO growth charts since 2007 in place of the older NCHS charts. The WHO growth charts had values of healthy breast fed babies (having relatively faster growth in the first 6 months than bottle fed babies), whereas NCHS represented bottle fed babies ^46^. Switching over to the WHO values resulted in lower weights for length at less than 6 months (and much higher values of wasting) among Indian babies, majority of whom paradoxically are breast fed. The paper also reported much higher levels of low BMI and anaemia in mothers as well as higher low birth weights in India when compared to Guatemala. The authors acknowledged absence of a satisfactory explanation apart from poor status of women, poor dietary quality, poor nutritional parameters or the “thin fat Indian baby” phenotype ^46, 47^. On examining the composite FAOSTAT dataset we created, Guatemala has maize as the top crop in contrast to India ^45^. Interestingly, like Guatemala, high stunting and lower wasting is also seen in Burundi and Timor-Leste which also have maize as top staple cultivated. Probably, proximate dietary factors hold the clues to these differences in spatiotemporal prevalence patterns of malnutrition between geographies within India and between nations.

### Ecogeographic patterns of clustering in India

There appears to be a discernible clustering of districts with stunting distinct from wasting in the country ( Figure 1). Greater stunting prevalence is mostly seen in the populous Northern states, which account for more than 80% stunted children in the country ^42^. High stunting prevalence was seen in Bihar and Uttar Pradesh at prevalence rates of 48.2% and 46.3% respectively ^42^. Areas with high prevalence of wasting are seen predominantly clustered in Central and Western Indian states of Gujarat, Maharashtra, Jharkhand, Madhya Pradesh and Rajasthan with greater dependence on rainfed agriculture ^48^. In India, child malnutrition prevalence, particularly stunting, has been explored spatiotemporally at household, village, block/ *taluk*, district, parliament and legislative constituency levels ^49– 54^. It has been studied with respect to clustering related to poverty, wealth inequality, low birth weight, maternal stature or low BMI ^55– 59^. However, these studies do not satisfactorily explain the contrasting clustering patterns of wasting vis-à-vis stunting across districts and states.

Cesar Victora in 1992 demonstrated that stunting and wasting are not necessarily co-occurring to a similar extent across geographies; regions with comparable stunting may in fact report several fold variations with corresponding wasting prevalence indicating diverse pathways to these two conditions ^60, 61^. Frongillo *et al.* analysed these differences between regions with stunting and wasting and found that they were eliminated when social, demographic and economic factors were taken into account ^61^. Both Victora and Frongillo *et al.* studies concluded that stunting and wasting could have different causes ^60, 61^. However, when comparing India and Bangladesh with respect to wasting, Frongillo *et al.* concluded that India had higher than expected and Bangladesh, on the contrary, had a lower than expected prevalence. They conclude that other factors not incorporated in their study possibly explained this difference ^61^. More than two decades later the differences with respect to under 5 wasting between India and its South Asian neighbours have remained persistently high, with India recording 21.04 % (as per NFHS4) wasting while others have figures ranging from 9.48% to 14.36% ^4^.

### Individual and geographic co-occurrence patterns of stunting and wasting

Presence of both stunting and wasting concurrently is called WaSt and its wide prevalence has been increasingly recognised recently ^39, 62– 65^. In our study 21 districts were identified to have high prevalence of both stunting and wasting ( Table 2). This is not WaSt *per se* but districts/populations reporting high prevalence of both *separately*. These 21 districts were from the high prevalence central and north-western states of Gujarat, Madhya Pradesh, Rajasthan and Jharkhand with only two each from Uttar Pradesh and Bihar. On examining the staple cereals cultivated in these districts, majority either cultivated Maize, Jowar (sorghum), other millets or Bajra (pearl millet) as one among the top two crops ( Table 2). However, among the four Uttar Pradesh and Bihar districts, one cultivated Bajra (Chitrakoot) as the second common crop but the others were predominantly rice and wheat cultivators.

Similar to the Indian cultivation patterns, a spate of recent studies on areas with WaSt also show predominant millet and sorghum cultivation. A prevalence survey of Karamoja region in Uganda, with Sorghum and maize as staple, showed a WaSt prevalence of 5% ^65, 66^. A recent study of children under 2 years in Madaraounfa in rural Niger, also with millet and sorghum as staple, showed 80% stunting, 14% wasting and 12% having concurrent wasting and stunting ^62^. Garenne *et al.* studied concurrent wasting and stunting among under 5 children in Niakhar, Senegal, which too has millet as staple ^39, 67^. Concurrent WaSt was found prevalent to the tune of 6.2% with a peak at 18 months in the study ^39^. A meta-analysis of prevalence of WaSt in 84 countries showed prevalence above 5% in 9 countries with three from Asia (India, Timor-Leste and Yemen) and six from sub-Saharan Africa (Niger, Djibouti, Burundi, Chad, Sudan and South Sudan) ^63^. On assessing them for crop cultivation or production, the highest ranking crops by area or production were Millet or Sorghum for Niger, Chad, Sudan, South Sudan and Yemen ^45^. For Djibouti, Burundi and Timor-Leste the top crop produced was Maize. India had by far the highest production of Sorghum and millet among all countries in the group, but these cereals trailed behind the figures for rice and wheat ^45^. We hypothesise that subsistence cereal cultivation in areas with high wasting and its use as staple particularly by pregnant and breast feeding mothers could account for this pattern.

The longitudinal study on WaSt of four decades of growth data in rural Gambia showed that wasting earlier increased the odds to stunting later after 3 months by a factor of 3.2 ^64^. In contrast, the odds to stunting associated with wasting after 3 months, was 1.6 ^64^. Hence, the stunted and wasted districts are more likely to have wasted children who later developed stunting. So we analysed the differences in quantity of crops cultivated between the high prevalence of wasting and high prevalence of stunting (only) districts, after excluding the 21 stunting and wasting districts from the list of high prevalence of stunting districts. The bar charts ( Figure 9A) clearly show the greater cultivation of coarse cereals (Bajra, Jowar and other millets) in the 112 high wasting districts in comparison to the 86 high stunting (only) districts.

Cereal-based diets are known to be associated with malnutrition and have been linked to Pellagra especially diets exclusively dependent on Sorghum and maize ^16, 68– 70^. Our results linking district-level wasting prevalence with cultivation of Jowar (Sorghum) and Other millets, and district-level stunting with wheat (rice cultivation did not significantly affect either stunting or wasting prevalence at district level) in the background of the discussion above indicate the need for household-level type of cereal consumption data to explain the malnutrition patterns. On the other hand, our study shows a negative association of bajra with under 5 wasting in Punjab and Haryana which needs explaining, given the overall pattern of district level wasting association with millet cultivation. Unlike other high millet cultivating regions which are semi-arid and practice rain-dependent agriculture, these states on the other hand are well irrigated and possibly grow Bajra for non-food purposes (feed, fodder and fine grain alcohol). This is estimated to be to the tune of 60% of total production of the country ^31^. The significant negative association of wheat with stunting could be due to reduced zinc intake linked to high phytates in wheat ^71– 73^.

The dietary diversity scores and maternal education levels are expectedly negatively associated with prevalence of stunting and wasting like reported earlier ^53, 74– 77^. Negative association was seen with rural residence for wasting which is contrary to the results of Harding *et al* ^4^. This could be a result of our choice of variables which modified the effect of rural residence. However, in a study comparing undernutrition in urban poor neighborhoods with rural areas in Maharashtra, wasting prevalence was higher in urban neighborhoods ^78^. Low women’s BMI was expectedly positively associated with under 5 wasting which is consistent with several other studies ^21, 79– 81^. Similarly women’s short stature was positively associated with stunting as reported earlier ^80– 83^. Open defecation and poverty too has been shown in various studies to be associated with under 5 wasting and stunting ^21, 57, 59, 75, 76^. However, in our study on adjusting for multiple variables, the association with poverty for both stunting and wasting was not statistically significant. A study done among Anganwadi centres (AWC) in North East India documented higher rates of stunting, wasting and underweight among 510 randomly selected children suggesting greater food insecurity among those utilizing AWCs ^84^. Food insecurity and access sought by food insecure families to AWC services could explain the positive association seen in our analysis with utilization of AWCs for under 5 wasting.

### Food processing and nutrient availability of cereals

In India both Sorghum and pearl millet are consumed by milling followed by bran removal and dry heating ^85^. This is known to adversely affect cereal protein availability, particularly in Sorghum, by Maillard reaction and Lysino-alanine like product formation ^86^. However, soaking overnight and boiling to 90 degree C can yield high percentage availability of available lysine for both pearl millet and Sorghum ^87, 88^. Unlike the practices in India, Maize is consumed in Latin America after nixtamalization ^89^. Indeed, cereal processing practices could contribute to high stunting and wasting seen in some districts with maize as staple ( Table 1).

The lower lysine scores of coarse cereals could be the key to higher levels of wasting and stunting in areas where they are staple. This could be mediated by molecular mechanisms ^90^. On comparing the digestible indispensable amino acid scores (DIAAS) of rice and wheat vis-à-vis millets and Sorghum, Lysine scores are higher in the former (table by Hans Henrik Steyn reproduced in our composite dataset) ^45^. With regards to micronutrient availability coarse cereals have higher micronutrient content than rice and wheat ^45, 68^. However, there is a known association of Jowar and Maize with Pellagra ^16, 69^. Clearly, there is marked variability in nutritional availability of glucose, amino acids, zinc, iron and other micronutrients among cereals ^45, 70– 73^. This warrants closer scrutiny of the dietary matrices of populations whose diet is mainly cereal based. See for instance, the nutritional benefit from ready to use therapeutic foods (RUTF) in children with acute malnutrition. RUTF formulations made from soya-maize-sorghum (SMS) show similar efficacy for malnutrition only when they are supplemented with free amino acids ^91– 93^. While millets and Sorghum’s lower glycaemic indices are suitable for elderly users their lower provision of amino acids and glucose could be detrimental for growth during the first 1000 days of life ^90, 94, 95^. This could be mediated by protein kinases, the mechanistic target of Rapamycin (MTORC1) or General control nonderepressible 2 (GCN2 ) as seen in the placenta in the case of intra-uterine growth retardation ^95^. Of these, MTORC1 has also been postulated as a possible cellular mechanism for stunting ^90, 95– 97^. A plausible hypothesised pathway on mechanisms of stunting and wasting through cereal based diets has been separately prepared ^45^.

### Study limitations

An important limitation of our analysis is the limited fine scale data on food grain consumption (as opposed to cultivation) which would have allowed for confirmation of our hypothesis at household level. One of the reasons for this is that the NFHS and other country/regional demographic health surveys record cereal consumption without paying attention to type of cereal. Moreover, consumption is likely to be guided by choice and availability through food subsidy or open-market access to other cereals and food staples, apart from those cultivated for subsistence. Our analysis indicates the need for NFHS and demographic health surveys worldwide to include type of cereal consumption to gain better understanding of pathways to malnutrition. The use of cereal cultivation as a proxy for consumption too is likely to have introduced substantial errors, as some of the cultivation is for non-human use. Factors leading to lack of dietary diversity like poverty, prevalence of infections like worm infestations or tuberculosis and other possible unaccounted confounding factors could also be contributing to these patterns. The data on availability of nutrients from cereal consumption from nutritional assays (stable isotope-based) is also meagre to the best of our efforts in reviewing peer-reviewed evidence-base. Such data from cereal consumption could help in linking the dietary matrix to the effects described above.

## Conclusion

Higher wasting and stunting prevalence among children in India has an ecogeographic pattern with plausible links of pre-dominant millet consumption to higher prevalence of wasting. The type of cereal consumed should be incorporated in NFHS and all global demographic surveys to enable better assessment of nutritional intake. State of the art research in nutrient sensing should be integrated with agriculture, food science, delivery systems and dietary matrix for translational benefits to accrue to the wider population.

## Data availability

### Underlying data

Figshare: Dataset used to assess relationship between millet cultivation and malnutrition patterns in India at district level. https://doi.org/10.6084/m9.figshare.12236789.v2 ^29^

This project contains the following underlying data:

- malnutrition_dataset_for_publication.xlsx (Dataset used for analysis described in the paper)
- Malnutrition and millets – India – DACNET NFHS 4.docx (Word document explaining how the dataset was prepared)

### Extended data

Figshare: Plots examining relationship between type of millet cultivated with stunting and wasting at district level along with map showing the overlaps for each type of millet with stunting and wasting. https://doi.org/10.6084/m9.figshare.12206135.v6 ^98^

This project contains the following extended data:

- Malnutrition_millets and malnutrition.pdf (PDF file with panel of seven plots and maps, each showing relationship between type of millet cultivated with stunting and wasting at district level and the corresponding map showing the overlaps of each type of millet with stunting and wasting)

Figshare: Plots examining relationship between low BMI and short stature in women 15–49 with stunting and wasting at district level along with map showing the overlaps for each type of millet with low BMI and short stature in women (15–49). https://doi.org/10.6084/m9.figshare.12206264.v4

This project contains the following extended data:

- malnutrition_bmi_short_status.pdf (PDF file with panel of seven plots and maps, wach showing relationship between low BMI and short stature in women 15–49 with stunting and wasting at district level alogn with maps showing overlaps for each type of millet with low BMI and short stature in women (15–49))

Data are available under the terms of the Creative Commons Attribution 4.0 International license (CC-BY 4.0).

## Software availability

Source code available from: https://gitlab.com/asdofindia/malnutrition-crops-maps

Archived source code at time of publication: http://doi.org/10.5281/zenodo.5976145 ^36^

License: MIT license
