# Supplementary material for: Eco-geographic patterns of child malnutrition in India and its association with cereal cultivation: An analysis using demographic health survey and agriculture datasets
Source: Wellcome Open Res. 2022 Feb 22;5:118. Originally published 2020 Jun 2. [Version 4] doi: 10.12688/wellcomeopenres.15934.4 (PMC9194519; doi:10.12688/wellcomeopenres.15934.4)
Supplement: Peer review history

# Peer review: Reviewer response for version 4

**DOI:** 10.21956/wellcomeopenres.19560.r50740

**Published:** 2022-06-13

**Authors:** Ilana Cliffer

**Affiliations:** 1 Harvard T.H. Chan School of Public Health, Boston, MA, USA

**Overall comments: **       

 This is a creative paper that uses district-level data to link area used for cereal cultivation of different types of cereals to wasting or stunting prevalence, using cereal cultivation area as a proxy for cereal consumption. The findings are interesting and relevant, however, the authors do not present a clear message as to the implications of their descriptive findings, nor do they sufficiently discuss the limitations of such ecological analyses based on many assumptions about the relationship between cereal cultivation and consumption.

 Two major points are missing, in my opinion:

 Is it possible that areas that cultivate one cereal over another may also have similar climatological features (temperature, precipitation) that impact the soil quality and type and also the prevalence of infectious disease? While I think the DSCQ is an interesting and clever metric, it is hard to separate any potential effects of cereal consumption from the underlying reasons why some areas cultivate one grain in favor of the other, and whether these factors could be related to other determinants of nutritional status. This merits discussion and exploration in this paper.

 The authors need to spend some time explaining why cereals were the only crops/dietary factors examined. No mention at all is made of vegetables, or other more nutrient-dense crops. What about livestock keeping? Animal-source foods? It seems impossible to draw conclusions even about associations with type of cereal without considering other dietary factors that may be equally or even more important.

 Several more specific comments follow, as well:

**Abstract**

1. Background
  1. Does the high prevalence of maternal malnutrition and child malnutrition include only undernutrition, or also overweight/obesity? Please clarify or use the term undernutrition if that is more accurate.
  2. Rural India has disproportionately high levels of child malnutrition compared to where? Urban India? Rest of the world?
  3. Stunting and wasting are not determinants of child malnutrition, they are indicators
  4. The background section of the abstract needs to explain the motivation behind the study better. It goes from stating that there is a high burden of malnutrition that is not evenly geographically distributed to talking about how cereals make up the bulk of protein intake, but doesn’t state the proposed connection between the two. It needs to be clarified why the authors are mentioning cereals as a proportion of the diet.  
2. Methods
  1. Methods section of abstract does not mention statistical techniques used – this would be helpful to mention
  2. Please also add sample size to methods (how many districts are included?)
  3. State in the methods of the abstract how under-5 stunting and wasting prevalence was disaggregated by age, since this comes up in the results
  4. Please also state in the methods of the abstract what cereals are analyzed (millets, wheat, etc.) or at least state that the investigation is of the relationship between prevalence of undernutrition and area under cultivation of several different cereals.
3. Results:
  1. Districts with higher prevalence of wasting – please define what this means. Would be useful to include a few specific statistics. Perhaps the range of prevalence in the highest vs lowest prevalence regions?
  2. Was the prevalence of wasting only higher among infants of 6 months old in districts with higher prevalence of wasting, or was it higher at all age ranges and especially at 6 months? Please make sure this is clear. Also what age ranges is this compared to? (All other children under 5?)
  3. When comparative phrases such as “stronger” or “higher” are used, it is hard to interpret without knowing what the comparison is. For example, the phrase stating that wasting prevalence has a stronger association seen for jowar and other millets, it would be helpful to know what this is compared to. Either that, or make the statement absolute.
  4. “District level stunting was associated with higher…”. Does this mean higher district level stunting was associated with higher district level cultivation of wheat?
  5. Does higher cultivation of cereals means more area under cultivation? It could be better to use more specific terms for what higher cultivation means.
  6. Saying wasting was positively associated with women’s body mass index is confusing, because what you actually mean is with women’s body mass index <18.5, correct? Please specify, otherwise it looks like in districts where women’s BMI was higher, there was *more* child wasting, which is not the case. 
4. Conclusions

- It seems to me that prior to a statement about what future studies are needed, the authors should summarize the main findings and their implications.

**Introduction**

1. Overall, the focus of the paper/study is not very clear, nor is the motivation to do the study. This needs to be more clearly defined and explained in the introduction. One thing that could help is if the introduction had a more logical organizational flow. For example, the authors may first want to discuss the high prevalence of undernutrition in South Asia (or India) compared to other regions (what is currently the last paragraph of the first section of the intro), then discuss its uneven distribution across the different districts (currently the second paragraph under the “subsistence farming and millet dependence” sub-heading, and phrase about hot-spots of low women’s BMI), and then discuss potential reasons for these disparities (differential cereal availability - the first paragraph in the “subsistence farming and millet dependence” sub-heading and the last paragraph about geospatial heterogeneity). Then the authors can state the research problem and specific research question in the last paragraph, and include a phrase about why answering this question will be beneficial.
2. The discussion of low birth weight in the first paragraph of the introduction seems out of place to me. Unless a link is made that explicitly discusses intergenerational transmission, talk of birth weight can be left out of the introduction since birth weight is not included as an outcome of interest in the study.
3. In the first line of the introduction, I suggest specifying that *population-level* undernutrition is indicated by prevalence of stunting, wasting, underweight.
4. Perhaps clarify how reference to the 2008 Lancet series on child malnutrition which mentions that areas with similar levels of stunting have different prevalence of wasting is relevant to the research question.
5. Please provide more specifics about what kind of subsistence crop cultivation was linked to seasonal epidemic nutritional edema among American farmers in the 1930s. Was the type of crop important in these studies from the 1930s?
6. Last phrase of the introduction about Ragi can be moved to the methods section.

**Methods**

1. Please clarify the year that data were collected for the NFHS4
2. Could the effects of DSCQ on district-level prevalence of stunting and wasting be modified by age? Was the age distribution even across all districts?

Table 1

- Variables like diet diversity could be a mean, so it should be clarified that all variables in this table are proportions of binary variables. (And/or call it minimum diet diversity in this table, as is done elsewhere).

Results

- “Wasting showed an early onset with highest wasting (40%) at 6 months of age”. I would suggest changing the wording from early onset, which implies that the data were longitudinal, to “wasting was highest among the youngest children (6 months of age) at the time of measurement”.

**Discussion **

1. The discussion should start with a clear summary (not repetition) of the results – a few phrases that summarize the main findings before moving on to put them into context of previous research and/or hypothesis related to the mechanisms behind their findings. The first paragraph of the discussion seems out of place to me. It can be shortened and worked into the section on individual and geographic co-occurrence patterns of stunting and wasting. The authors also do not really explain how cereal consumption in itself would be related to the relationship between stunting and wasting.
2. As it is written, the discussion is hard to follow, with many seemingly irrelevant details presented outside the context of the objective of the paper. It would be good if the discussion first outlined how the results in this paper are related to previous findings or not, and then discussed potential explanations for the findings, including mechanisms through which millet consumption may be associated with higher stunting or wasting prevalence. While there is some discussion at the very end about potential mechanisms through which millet consumption may be related to higher levels of stunting and/or wasting, this section is confusing and it is unclear how exactly millet consumption would be inferior, given statements about processing of rice or maize that would seemingly make those the inferior grains to consume.
3. A lot of the discussion is re-stating results instead of providing context and explanation for the results. For example, the age and geographical patterns of stunting and wasting section starts off with a paragraph stating specific results that could be summarized in one phrase and then merged into discussion that couches the findings in the literature and discusses implications.
4. “Probably, proximate dietary factors hold the clues to these differences in spatiotemporal prevalence patterns…”. This is a strong statement and a stretch in my opinion. It is possible, but saying that it is probable overstates a relationship based solely on correlations and understates/doesn’t explore other potential explanatory factors. In fact, the authors need to stress and respect that they cannot make causal inferences from these analyses, given their study design but also the fact that they use cereal cultivation as a proxy for consumption.
5. The limitation of using cereal cultivation as a proxy for consumption needs to be afforded much more discussion, as this is based on a lot of assumptions and is a major limitation to interpretation of these findings.
6. Authors could comment in the limitations section on how it may impact their results that census data were from 2011, the NFHS4 data were from (2015-2016?), and the crop data ranged from 2002-2017.

Is the work clearly and accurately presented and does it cite the current literature?

Partly

If applicable, is the statistical analysis and its interpretation appropriate?

Partly

Are all the source data underlying the results available to ensure full reproducibility?

Yes

Is the study design appropriate and is the work technically sound?

Partly

Are the conclusions drawn adequately supported by the results?

No

Are sufficient details of methods and analysis provided to allow replication by others?

Yes

Reviewer Expertise:

maternal and child nutrition, child growth, seasonality, food systems

I confirm that I have read this submission and believe that I have an appropriate level of expertise to confirm that it is of an acceptable scientific standard, however I have significant reservations, as outlined above.

# Peer review: Reviewer response for version 4

**DOI:** 10.21956/wellcomeopenres.19560.r48847

**Published:** 2022-04-04

**Authors:** Edward J.M. Joy

**Affiliations:** 1 Faculty of Epidemiology and Population Health, London School of Hygiene & Tropical Medicine, London, UK

Thanks to the authors for their further revisions to the article.

Is the work clearly and accurately presented and does it cite the current literature?

Partly

If applicable, is the statistical analysis and its interpretation appropriate?

No

Are all the source data underlying the results available to ensure full reproducibility?

Yes

Is the study design appropriate and is the work technically sound?

No

Are the conclusions drawn adequately supported by the results?

No

Are sufficient details of methods and analysis provided to allow replication by others?

Yes

Reviewer Expertise:

Dietary micronutrient assessment, population micronutrient assessment

I confirm that I have read this submission and believe that I have an appropriate level of expertise to confirm that it is of an acceptable scientific standard.

# Peer review: Reviewer response for version 3

**DOI:** 10.21956/wellcomeopenres.18904.r46237

**Published:** 2021-11-10

**Authors:** Edward J.M. Joy

**Affiliations:** 1 Faculty of Epidemiology and Population Health, London School of Hygiene & Tropical Medicine, London, UK

- In the abstract, the authors suggest that ‘better data’ are required to confirm a causal pathway. What is actually required is a well-designed intervention study. Please revise.
- In the methods, the authors write “Other socio-demographic variables included in our analysis have been listed above…”. It would be clearer to specify them here, and the authors should also specify whether these variables were controlled for in the multivariate regression analysis.
- In the methods where the outcome variables are specified, the z-scores should be ‘negative 2’
- In the study limitations, the authors write “The use of cereal cultivation as a proxy for consumption too is a source of noise in our data…”. I don’t think ‘noise’ is the right term. It is likely to introduce substantial error and is a major limitation of the study.

Is the work clearly and accurately presented and does it cite the current literature?

Partly

If applicable, is the statistical analysis and its interpretation appropriate?

No

Are all the source data underlying the results available to ensure full reproducibility?

Yes

Is the study design appropriate and is the work technically sound?

No

Are the conclusions drawn adequately supported by the results?

No

Are sufficient details of methods and analysis provided to allow replication by others?

Yes

Reviewer Expertise:

Dietary micronutrient assessment, population micronutrient assessment

I confirm that I have read this submission and believe that I have an appropriate level of expertise to confirm that it is of an acceptable scientific standard, however I have significant reservations, as outlined above.

# Response

**Published:** 2022-02-22

**Authors:** Prashanth N Srinivas

**Affiliations:** Institute of Public Health Bengaluru, India

Thank you for your review. 

 Regarding suggestion on revision of abstract, the revised abstract addresses this comment. 

****

 Regarding the clarification on variables, listing them and on the details of controlling for them in the multivariable regression, the variables have been listed in Table 1 and mention has been made of them being controlled in multivariable regression.

 The error regarding z-scores is regretted and fixed. 

 Amendments pointed out In the study limitations ****has been addressed.

 In addition to this, extensive revisions have now been made in the Discussion section to address other reviewer concerns as well and we hope these have comprehensviely addressed the observations in the reviews.

# Peer review: Reviewer response for version 3

**DOI:** 10.21956/wellcomeopenres.18904.r46243

**Published:** 2021-10-26

**Authors:** Nicholas J. Kassebaum

**Affiliations:** 1 Institute for Health Metrics and Evaluation, University of Washington, Seattle, WA, USA

This study reports on associations between grain cultivation patterns and patterns of HIGH wasting and HIGH stunting prevalence. This is a useful addition to the field of nutrition and child anthropometrics, which ironically has not focused as much on food supply as on other factors. However, I have a few concerns:

 Small points:

1. Opening sentence of intro is very difficult to read. After several tries, I’m still not sure what it is trying to say.
2. Last sentence of intro says NFHS4 was “published” in 2015. Wasn’t it conducted in 2015-2016 and published in 2018?

More substantial comments:

1. Figure 1- the legend is so similar for the three categories that it is very difficult to tell them apart. Suggest to revise to increase legibility.
2. Birth weight data for children <5 were routinely collected in NFHS-4. The authors state that this is “unavailable.” Please explain.
3. Choice of “high” vs. “low” is a bit arbitrary. There is nothing in the paper by Khan and Mohanty (other than their making severity classifications in a table), or indeed in the NFHS-4 anthropometrics data itself, that suggests a particular inflection point where the authors of this study have defined “high” wasting / stunting. Stunting/ wasting/ underweight are already over-simplified to be dichotomous. Further dichotomizing to high vs. low prevalence locations may be a simplification that makes the analysis easier, but it substantially weakens the broad applicability of the findings of the paper. Can the authors comment on why they were unable to perform this as an analysis where the dependent variable was not also/ instead chosen to be continuous z score?
4. Discussion doesn’t talk at all about the results of the paper and its implications, instead focusing on nutrition science. Suggest to revise and focus on this paper. Summarize the biology/ clinical information much more briefly and refer interested readers to where they can find this additional information. The tables and figures in the discussion do not add to the narrative. Figure 9 in particular – these don’t look substantially different; if there is an important difference to show, this needs to be a different visual (and moved to the results).
5. The most important finding of this analysis has largely been glossed over in both the results and the discussion. This is presented in Table 2 and Table 3, where the authors’ multivariate analysis suggests that the previously described association between sociodemographic factors (poverty, education, rural status, open defacation) are all actually [potential] proxies for dietary diversity as evidenced by dramatically reduced – or reversed – beta coefficients in multivariate regressions. What is the authors interpretation of this finding? This contradicts, or at least refines, the findings of Khan and Mohanty (cited by authors, ref 18) and many others. If true, the implications of this are potentially profound. What are the potential policy implications? What does the literature say about the mechanistic association between sociodemographics and food supply – is it more effective to target education/ poverty/ living conditions? Or more effective to target food supply and dietary diversity? What would be the next steps in gathering data to answer this question if unknown?

Is the work clearly and accurately presented and does it cite the current literature?

Partly

If applicable, is the statistical analysis and its interpretation appropriate?

Partly

Are all the source data underlying the results available to ensure full reproducibility?

Yes

Is the study design appropriate and is the work technically sound?

Yes

Are the conclusions drawn adequately supported by the results?

Partly

Are sufficient details of methods and analysis provided to allow replication by others?

Yes

Reviewer Expertise:

child health, epidemiology, health metrics

I confirm that I have read this submission and believe that I have an appropriate level of expertise to confirm that it is of an acceptable scientific standard, however I have significant reservations, as outlined above.

# Response

**Published:** 2022-02-22

**Authors:** Prashanth N Srinivas

**Affiliations:** Institute of Public Health Bengaluru, India

Thank you for your detailed review. 

 On the minor points you have highlighted:

1. We have made suitable edits to improve readability. Regarding teh 
2. Error related to year of NFHS4 regretted and corrected **.**

Response to major/substantive comments below; extensive changes made in v4 to address these: 

1. Figure 1 has been re-done the figures to improve visual contrast of the pattern **. **
2. References to birth weight data from NFHS have now been removed as we do not use it in our analysis.
3. On the assertion regarding arbitrariness of “high” vs. “low” stunting/wasting districts: In the paper by Junaid and Mohanty, the districts were classified as low stunting (less than 26%), medium stunting (prevalence 26.1-46%) and high stunting (prevalence higher than 46%) based on mean and standard deviation, as per their results. Highest wasting prevalence districts  were categorised as more than 28%. We utilised this  classification for illustrating the districts with high prevalence of stunting, wasting as well as both high stunting and wasting in the maps. However, Z scores of less than -2 SD have been used for stunting & wasting cut off to assess prevalence in NFHS4. Their  prevalence in districts (without distinction into high and low) have been used for the scatter plots and multivariable regression. A table (table 1) indicating different variables used in the maps and other results is shown for clarity in this version. We accept that our choosing of these cut-offs will affect the wider applicability of these findings. However, in the lack of a globally comparable standard, we have chosen this. Furthermore, this categorisation is only applicable to our map-based visualisations. The multivariable analysis indeed uses district-level percentages of stunting and wasting.  
4. On the points raised in Discussion that are not in the results: We have re-written the Discussion section to focus better on the results reported. WE have now removed any tables and figures from our discussion and have modified earlier figure 9, a pie chart into a bar and moved it up to the results. The figure showing pathways to nutrition and information about mechanisms through MTORC1 has been only referred to and all narrative/figures on the pathway have now been shifted out of the paper.
5. On the reviewers points about findings presented in Table 2 and Table 3: Thank you for the opportunity to clarify this further and thank you for your comments on the potential applications of our findings. In our revision, we have attempted to address these important comments to the extent possible. However, some of these comments being policy and practice implications of the underlying hypotheses we are examining, we have been unable to comprehensively address them even in our revised discussion. Moreover, many of these important observations by the reviewer are in age-groups outside of our analysis (nonetheless having important implications). We hope that our paper shall contribute to expanding the literature on the points raised by the reviewer. However, given the importance of these points, we attempt to provide some response hereunder to those aspects that are yet not addressed in our revised discussion. We hope to develop these further once this analysis achieves peer acceptability.
6. The literature and evidence on food security potentially connects socio-demographic factors mechanistically to food supply. One definition of food security is “Access by all people at all times to sufficient food and nutrition for a healthy and productive life” ^1^ . The opposite term, food insecurity has been defined by one source as “A person is food insecure when they lack regular access to enough safe and nutritious food for normal growth and development and an active and healthy life ” ^2^.   Moderate food insecurity, like seen among the poor in high-income countries, is also associated with obesity ^3^ . Severe food insecurity is associated with hunger. A study based on data suitable for measuring food security from Maharashtra, India  indicated that food insecurity is no longer associated  with statistically significant stunting, wasting or underweight, when controlled for dietary diversity and all other child, maternal or household characteristics implying  the importance of dietary diversity as a determinant of malnutrition ^4^.
7. The Lancet 2013 series clearly brought that the Nutrition specific interventions , when brought up to scale of over 90% could help tackle about 20% of global burden of malnutrition ^8^. Noticeably, the emphasis in the Lancet series of both 2008 & 2013 was on the first 1000 days as far as Nutrition specific interventions are concerned ^8,9^. However, longitudinal studies point to the fact that children both become stunted and recover in growth, from infancy through adolescence, thereby extending the period of intervention for healthy growth from the first 1000 days till adolescence ^10,11^. Greater persistent stunting after 5 years till 15 years, in one study in India and Ethiopia vis-à-vis Peru and Vietnam, points to an environmental context in the former countries ^11^. India also has the largest number of low birth weight  among countries in the world, a reflection on the health and nutrition of women through their lives ^12^. A study analysing secular trends in improvement of child malnutrition indices in Vietnam and Bangladesh, attributed rapid improvements in HAZ scores to changes in underlying determinants like socioeconomic status, maternal education, hygiene and food security ^13^. Changes in  maternal nutritional knowledge and child dietary diversity were associated with only 20% of the total change ^12^. A cross country study about drivers of nutritional change suggested four factors which predict reduction in undernutrition as, women’s secondary education, reduction in fertility, increased access to health services and increase in household assets ^14^. Clearly, a combination of nutrition sensitive and specific measures as per context would be the way forward ^15^.
8. The Integrated Child Development Services (ICDS) of India covers a wide range of services involving teenage nutrition, pre-school feeding, health and education as well as nutrition of pregnant & breast feeding mothers, conducted through 1.3 million Anganwadi centres all over India ^16,17^. In 2006, the ICDS was scaled up substantially following a Supreme Court directive ^18^. There was a significant improvement in ICDS coverage as seen through comparison of NFHS 3 & 4 after this scaling up. A threefold increase in percentage of women and children receiving food supplementation during pregnancy, lactation and early childhood was noted during the period ^17,18^.There were also significant improvements recorded in breast feeding practices, antenatal coverage, immunization, age of marriage, women’s education and several other health related practices ^18^.  Concomitantly, a 10 percentage reduction in stunting in children aged 0-5 years  was recorded in 2016 vis-à-vis 2006 ,as per NFHS data ^17,18^. The improvement is heterogenous across states of India, with intra-state disparities seen particularly among the poor and lower castes. However, in IYCF practices, complementary feeding and minimal meal frequency showed decline in 2016 over 2006. Clearly, the ICDS is a work in progress and there is much room for improvement with respect to coverage and efficacy ^17,18^.
9. A  meta-analysis on school feedings showed significant improvements in height and weight over 12 months , compared to controls ^19^. A recent study has clearly indicated substantial benefits of India’s mid-day meal (MDM)  scheme,  in estimated improvement in stunting up to 0.4 SD among children born to mothers with greater exposure to MDM ^20^. This points to the intergenerational benefits of  school feeding apart from its multisectoral economic benefits which extend from health, social protection, education, fertility reduction to local agriculture ^20,21^.A closer look at countries which have shown impressive declines in stunting and other mother and child nutrition related parameters, such as China and Peru , show presence of robust school feeding programs including provision of milk , over the last few decades ^22,23^. Improving the quality of  school feeding (coupled with deworming , nutrition education etc)   and extending it to include adolescents could arguably bring about  undernutrition reduction substantially by synergising the nutrition specific and sensitive nature of interventions involved.
10. A practice like eating down in pregnancy, wherein mothers eat less for fear of labour related complications due to a large baby is not unheard of in many parts of rural India ^24^. Health care systems should be made more accessible and effective, particularly in terms of priority areas like vaccination services, as well as obstetric and child care. This will also help deal with hidden issues like maternal depression as well as  common infectious causes of child wasting such as tuberculosis, Malaria, diarrhoeas and respiratory infections ^25,26^. Replacement of iron folate  to multiple micronutrient supplements, targeted balanced energy micronutrient supplements to pregnant mothers with low BMI have been recommended ^8 ^.This could be extended to breast feeding mothers given the high rates of malnutrition in early infancy in India. ^^The MDM should be expanded to include older children with provision of a breakfast with milk to start the day. Greater emphasis on play at ICDS and school  should address both child stunting and rising prevalence of obesity(twin burdens of malnutrition) well. Conditional cash transfers for poorer households like the Oportunidades in Mexico ^27^, could be used to improve dietary diversity among women and children of the poorer states through encouraging use of Nutrition, education and health services. Optimum resource allocation for multisectoral improvements  in both nutrition specific and sensitive areas with close monitoring of benefits to the targeted population nation-wide, could bring about transformational benefits.
11. Dietary matrix data like studies by Kurpad et al on commonly consumed food preparations by the poor with a focus on means to enhance nutrient availability through simple measures can be utilised for better quality nutritional advice or education ^28^. The effect of cereal type on the variability in nutrient(s) availability will have to be factored while designing/ recommending appropriate diets. Well-designed biofortification on a large scale could be explored to improve micronutrient levels in a population. Quality of breast milk , nutrient availability in the breast milk and maternal depression among mothers consuming different dietary matrices( like, say, by using different cereal types) would be immensely beneficial  to focus on type of diets needed by  lactating mothers in different ecogeographic areas . Given that most of the countries with high rates of malnutrition in early infancy have high cultivation of coarse cereals (our observation), it would be relevant and important to explore the relationship between type of cereal consumed and foetal growth restriction in communities with low dietary diversity and a cereal based diet. Considering that one third of Indian women in NFHS report never consuming eggs and 11% never consuming dairy products, research in optimal diet to support this group for the first 1000 days is urgently needed ^29^. Use of mobile services for personalised dietary advise to the general population is another unexplored avenue. Research into quality of India’s preschool services should be extended to states with poorer indicators of performance with an aim to translate best practices consistently across the states ^30,31^.

References:

 1. Gibson M. Food Security-A Commentary: What Is It and Why Is It So Complicated? Foods. 2012 Dec 3;1(1):18-27. doi: 10.3390/foods1010018. PMID: 28239088; PMCID: PMC5302220.

 2. FAO, IFAD, UNICEF, WFP and WHO. 2021. *The State of Food Security and Nutrition in the World 2021. Transforming food systems for food security, improved nutrition and affordable healthy diets for all*. Rome, FAO.

 3. Dhurandhar EJ. The food-insecurity obesity paradox: A resource scarcity hypothesis. Physiol Behav. 2016 Aug 1;162:88-92. doi: 10.1016/j.physbeh.2016.04.025. Epub 2016 Apr 26. PMID: 27126969; PMCID: PMC5394740.

 4. Chandrasekhar S, Aguayo VM, Krishna V, Nair R. Household food insecurity and children's dietary diversity and nutrition in India. Evidence from the comprehensive nutrition survey in Maharashtra. Matern Child Nutr. 2017 Oct;13 Suppl 2(Suppl 2):e12447. doi: 10.1111/mcn.12447. PMID: 29032621; PMCID: PMC6866156.

 5. Arimond M, Ruel MT. Dietary diversity is associated with child nutritional status: evidence from 11 demographic and health surveys. J Nutr. 2004 Oct;134(10):2579-85. doi: 10.1093/jn/134.10.2579. PMID: 15465751.

 6. Millward DJ. Nutrition, infection and stunting: the roles of deficiencies of individual nutrients and foods, and of inflammation, as determinants of reduced linear growth of children. Nutr Res Rev. 2017 Jun;30(1):50-72. doi: 10.1017/S0954422416000238. Epub 2017 Jan 23. PMID: 28112064.

 7. Roselló-Díez A, Joyner AL: Regulation of Long Bone Growth in Vertebrates; It Is Time to Catch Up. *Endocr Rev.*2015; 36(6): 646–80. PubMed Abstract | Publisher Full Text | Free Full Text

8. Bhutta ZA, Das JK, Rizvi A, Gaffey MF, Walker N, Horton S, Webb P, Lartey A, Black RE; Lancet Nutrition Interventions Review Group, the Maternal and Child Nutrition Study Group. Evidence-based interventions for improvement of maternal and child nutrition: what can be done and at what cost? Lancet. 2013 Aug 3;382(9890):452-477. doi: 10.1016/S0140-6736(13)60996-4. Epub 2013 Jun 6. Erratum in: Lancet. 2013 Aug 3;382(9890):396. PMID: 23746776.

 9. Black RE, Allen LH, Bhutta ZA, *et al.*: Maternal and child undernutrition: global and regional exposures and health consequences. *Lancet.* 2008; 371(9608): 243–260. PubMed Abstract | Publisher Full Text

 10 . Martorell R, Zongrone A. Intergenerational influences on child growth and undernutrition. Paediatr Perinat Epidemiol. 2012 Jul;26 Suppl 1:302-14. doi: 10.1111/j.1365-3016.2012.01298.x. PMID: 22742617.

 11. Gausman J, Kim R, Subramanian SV. Stunting trajectories from post-infancy to adolescence in Ethiopia, India, Peru, and Vietnam. Matern Child Nutr. 2019 Oct;15(4):e12835. doi: 10.1111/mcn.12835. Epub 2019 May 23. PMID: 31042809; PMCID: PMC7938413.

 12. Ramalingaswami V, Jonson U, Rohde J: The Asian Engima, the progress of nations. 1997.

 13. Nguyen PH, Headey D, Frongillo EA, Tran LM, Rawat R, Ruel MT, Menon P. Changes in Underlying Determinants Explain Rapid Increases in Child Linear Growth in Alive & Thrive Study Areas between 2010 and 2014 in Bangladesh and Vietnam. J Nutr. 2017 Mar;147(3):462-469. doi: 10.3945/jn.116.243949. Epub 2017 Jan 25. PMID: 28122930; PMCID: PMC5320405.

 14. Headey D. Developmental drivers of nutritional change: a cross-country analysis. World Dev 2013; 42: 76–88.

 15. Ruel MT, Alderman H; Maternal and Child Nutrition Study Group. Nutrition-sensitive interventions and programmes: how can they help to accelerate progress in improving maternal and child nutrition? Lancet. 2013 Aug 10;382(9891):536-51. doi: 10.1016/S0140-6736(13)60843-0. Epub 2013 Jun 6. Erratum in: Lancet. 2013 Aug 10;382(9891):506. PMID: 23746780.

 16. Rao N, Kaul V. India’s integrated child development services scheme: challenges for scaling up. Child Care Health Dev. 2018 01;44(1):31–40. doi: http://dx.doi.org/10.1111/cch.12531 PMID: 29235171

 17. Chakrabarti S, Raghunathan K, Alderman H, Menon P, Nguyen P. India's Integrated Child Development Services programme; equity and extent of coverage in 2006 and 2016. Bull World Health Organ. 2019 Apr 1;97(4):270-282. doi: 10.2471/BLT.18.221135. Epub 2019 Feb 25. PMID: 30940984; PMCID: PMC6438246.

 18. Nguyen PH, Avula R, Headey D, Tran LM, Ruel MT, Menon P. Progress and inequalities in infant and young child feeding practices in India between 2006 and 2016. Matern Child Nutr. 2018 Nov;14 Suppl 4(Suppl 4):e12663. doi: 10.1111/mcn.12663. PMID: 30499255; PMCID: PMC6518921.

 19. Wang D, Shinde S, Young T, Fawzi WW. Impacts of school feeding on educational and health outcomes of school-age children and adolescents in low- and middle-income countries: A systematic review and meta-analysis. J Glob Health. 2021 Sep 4;11:04051. doi: 10.7189/jogh.11.04051. PMID: 34552720; PMCID: PMC8442580.

 20. Chakrabarti S, Scott SP, Alderman H, Menon P, Gilligan DO. Intergenerational nutrition benefits of India's national school feeding program. Nat Commun. 2021 Jul 12;12(1):4248. doi: 10.1038/s41467-021-24433-w. PMID: 34253719; PMCID: PMC8275765.

 21. Verguet S, Limasalle P, Chakrabarti A, Husain A, Burbano C, Drake L, Bundy DAP. The Broader Economic Value of School Feeding Programs in Low- and Middle-Income Countries: Estimating the Multi-Sectoral Returns to Public Health, Human Capital, Social Protection, and the Local Economy. Front Public Health. 2020 Dec 3;8:587046. doi: 10.3389/fpubh.2020.587046. PMID: 33344398; PMCID: PMC7744483.

 22. Jacoby ER, Cueto S, Pollitt E. When science and politics listen to each other: good prospects from a new school breakfast program in Peru. Am J Clin Nutr. 1998 Apr;67(4):795S-797S. doi: 10.1093/ajcn/67.4.795S. PMID: 9537631.

 23. Huang X, Yang B, Liu Q, Zhang R, Tang S, Story M. Improving maternal and child nutrition in China: an analysis of nutrition policies and programs initiated during the 2000-2015 Millennium Development Goals era and implications for achieving the Sustainable Development Goals. J Health Popul Nutr. 2020 Dec 2;39(1):12. doi: 10.1186/s41043-020-00221-y. PMID: 33267909; PMCID: PMC7709233

 24. Nichter M. The ethnophysiology and folk dietetics of pregnancy: a case study from South India. Human Organization 1983; 42:235–246.

 25. Goutines J, Miller LC, Sorge F. Infections and nutritional status of internationally adopted children in France. Acta Paediatr. 2021 Apr;110(4):1359-1365. doi: 10.1111/apa.15612. Epub 2020 Nov 9. PMID: 33040353.

 26. Shidhaye P, Giri P. Maternal depression: a hidden burden in developing countries. Ann Med Health Sci Res. 2014 Jul;4(4):463-5. doi: 10.4103/2141-9248.139268. PMID: 25221688; PMCID: PMC4160664.

 27. . Morley, Samuel & David Coady, 2003. " From Social Assistance to Social Development: Targeted Education Subsidies in Developing Countries," Peterson Institute Press: All Books, Peterson Institute for International Economics, number cgd376, July.

 28. Kalasuramath S, Kurpad AV, Thankachan P. Effect of iron status on iron absorption in different habitual meals in young south Indian women. Indian J Med Res. 2013 Feb;137(2):324-30. PMID: 23563376; PMCID: PMC3657856.

 29. Nguyen PH, Avula R, Tran LM, Sethi V, Kumar A, Baswal D, Hajeebhoy N, Ranjan A, Menon P. Missed opportunities for delivering nutrition interventions in first 1000 days of life in India: insights from the National Family Health Survey, 2006 and 2016. BMJ Glob Health. 2021

 Feb;6(2):e003717. doi: 10.1136/bmjgh-2020-003717. PMID: 33627359; PMCID: PMC7908280.

 30. Isley, B.J. (2000). *Tamil Nadu Early Childhood Environment Rating Scale (TECERS). *Chennai,

 India: M.S. Swaminathan Research Foundation.

 31. Rao N. Preschool Quality and the Development of Children from Economically Disadvantaged Families in India. *Early Educ. Dev. *2010;21:167–185. doi: 10.1080/10409281003635770.[ CrossRef] [ Google Scholar]

# Peer review: Reviewer response for version 2

**DOI:** 10.21956/wellcomeopenres.18006.r41339

**Published:** 2021-06-09

**Authors:** Suparna Ghosh-Jerath

**Affiliations:** 1 Indian Institute of Public Health-Delhi, Public Health Foundation of India, Gurgaon, Haryana, India

I would request you to kindly transfer this paper for a second round of review by another reviewer for the following reasons: 

- I have gone through the responses provided by the authors. Though the authors have tried to respond to some of the concerns raised by both the reviewers, the core concern related to the basic hypothesis remains. The authors seem to be making the classic mistake of assuming causality from correlations with erroneous and troublesome conclusions. Further, these conclusions are based on several assumptions especially translating production into consumption, limited exploration of intra-household food distribution, access to other food sources (dietary diversity), nutrition sensitive factors and a simple fact that a nutritious diet cannot be constituted or assessed based on "only cereals", one needs to look at the diet in totality and production and access to a diverse food basket. If this message is  disseminated without further scrutiny, it may give strong negative messages countering the benefits of cultivating nutrient rich, climate friendly millets, that are sometimes grown as mixed cropping with other pulses/legumes and cereals, and are incidentally produced by several of the nutritionally vulnerable, marginalized, economically backward communities of India.
- Unfortunately, the authors fail to understand that production of cereal cannot be a proxy to actual consumption, which along with several other intervening factors (both nutritional as well as non nutritional) are resulting in poor nutritional status of vulnerable communities in question. 
- Most of the responses are deviating from the queries raised. Removing a nutrient rich millet from the analysis and assuming that the underlying cause of wasting could be coarse cereals seems somewhat indefensible.
- In case they wish to continue with the coarse cereal /millets and wasting argument, they need to highlight in discussion that pre-processing can address most concerns about the anti-nutritional effects e.g. as given in many papers, instead of trying to dissuade millet cultivation and use. ( https://fppn.biomedcentral.com/articles/10.1186/s43014-020-0020-5)
- In case the authors extensively revise the paper, they need to focus on all components of infant and young child feeding practices in totality along with other nutrition sensitive factors rather than focusing on "cereals only" and exploring association of wasting with cultivation of specific coarse cereals in isolation
- At its current status, the paper cannot be accepted for indexing.

Is the work clearly and accurately presented and does it cite the current literature?

No

If applicable, is the statistical analysis and its interpretation appropriate?

No

Are all the source data underlying the results available to ensure full reproducibility?

Yes

Is the study design appropriate and is the work technically sound?

No

Are the conclusions drawn adequately supported by the results?

No

Are sufficient details of methods and analysis provided to allow replication by others?

Yes

Reviewer Expertise:

NA

I confirm that I have read this submission and believe that I have an appropriate level of expertise to state that I do not consider it to be of an acceptable scientific standard, for reasons outlined above.

## References

[1] : Plant food anti-nutritional factors and their reduction strategies: an overview. *Food Production, Processing and Nutrition* .2020;2(1) : 10.1186/s43014-020-0020-5 10.1186/s43014-020-0020-5

# Response

**Published:** 2021-06-25

**Authors:** Prashanth N Srinivas

**Affiliations:** Institute of Public Health Bengaluru, India

In the paper we analyse data in order to explore the possible link between type of cereal consumed and the occurrence of child malnutrition, given that cereals constitute upwards of 70% of diet in the rural areas where malnutrition is the highest. We maintain that this is a novel exploratory study, as we have not come across a study that has explored this relationship.

 In the interest of advancing critical inquiry at the heart of the scientific process, we believe that overwhelming existing evidence should NOT be a reason for non-acceptance of new kinds of evidence demonstrating plausibility if not causation. A reasonable approach in our opinion for anyone with differences from the line of thought that we pursue here would be to demonstrate mistakes in our analysis and/or over-reach of conclusions (which in fact was pointed out by RI and has been significantly revised in our version 2). 

 The reviewer repeatedly brings out the argument that millets are nutritious and in the same breath says that malnutrition is more common in communities consuming millets (see for eg. R2 assertion in response to v1 "“It's not surprising that households with high coarse cereals consumption have high wasting and malnutrition”) This is in fact the relationship that we attempt to explain (we do not attempt to causally link the two). Such an explanation requires reaching across disciplines ranging from cellular pathways, agriculture and nutrition which we attempt in this paper.

 It is the dietary matrix and how it impacts on the absorption of various nutrients that is important as opposed to making millet a singular causal candidate. Our approach is that since there is substantial variability in the dietary availability of various key nutrients in different cereals there are likely to be differences in their effects in different populations. The low glycaemic index of some millets which may be advantageous to the obese diabetic may be a disadvantage to the unborn of a pregnant adolescent with poor dietary diversity. We have indicated in our version2 that most millets indeed have greater micronutrient levels in comparison to rice & wheat which could be contributory to stunting in areas where they are predominantly consumed. Our contention is that the lower amino acid availability could be similarly causing wasting in areas where millets are consumed. To emphasize this aspect, we have brought out the Digestible ingested amino acid scores(DIAAS), as acceptable by WHO, of different cereals from published literature. The correct approach mitigate this lack of nutritional availability by pre-processing like nixtamalization or fermentation or to supplement above by appropriate changes in the matrix or with added nutritional supplements in the way Iron and Folate is supplemented in pregnancies in most developing countries.

 So what we are asking is for a closer scientific scrutiny into the nutritional availability of key components in commonly consumed diets by rural Indians. To reject the question itself, as the reviewer has done in conclusion, demonstrates the lack of engagement with our analysis and results presented possibly due to an overwhelming reliance on the existing assertions about nutritious value of millets rather than an openness to engage with evidence critical of a given hypothesis. 

 We believe that our assertions are reasonably demonstrated by the evidence we present and were hoping to be proved wrong by demonstrating errors in our analysis or conclusions. In the lack of that, we shall continue working harder on communicating our findings better to the scientific community.

# Peer review: Reviewer response for version 2

**DOI:** 10.21956/wellcomeopenres.18006.r41340

**Published:** 2021-01-12

**Authors:** Edward J.M. Joy

**Affiliations:** 1 Faculty of Epidemiology and Population Health, London School of Hygiene & Tropical Medicine, London, UK

The authors have made substantial revisions which have largely addressed my earlier comments. I would prefer the authors to expand on the study limitations, which are inherent in a cross-sectional, ecological study design.

 The multi-variate analysis is a better approach. It appears that the association between wheat consumption and wasting is similarly strong as for jowar consumption, which I suppose illustrates the potential pitfalls with over-interpreting the findings and implying causality via a specific metabolic pathway driven by (anti-)nutritional factors in millets. It would be consistent with an underlying driver of poverty/wealth which will determine not only access to diverse diets but also other risk factors of undernutrition.

Is the work clearly and accurately presented and does it cite the current literature?

Partly

If applicable, is the statistical analysis and its interpretation appropriate?

No

Are all the source data underlying the results available to ensure full reproducibility?

Yes

Is the study design appropriate and is the work technically sound?

No

Are the conclusions drawn adequately supported by the results?

No

Are sufficient details of methods and analysis provided to allow replication by others?

Yes

Reviewer Expertise:

Dietary micronutrient assessment, population micronutrient assessment

I confirm that I have read this submission and believe that I have an appropriate level of expertise to confirm that it is of an acceptable scientific standard, however I have significant reservations, as outlined above.

# Peer review: Reviewer response for version 1

**DOI:** 10.21956/wellcomeopenres.17478.r39290

**Published:** 2020-07-27

**Authors:** Suparna Ghosh-Jerath

**Affiliations:** 1 Indian Institute of Public Health-Delhi, Public Health Foundation of India, Gurgaon, Haryana, India

The manuscript provides insight on impact of production of coarse cereals (selective millets) on the nutritional status of women and children in India.

 I have the following concerns with the methodology and conclusions drawn in this manuscript

1. Title: The title has issues in terms of including food (cereals) and nutrients (proteins and micronutrients) in the same basket, which is not clear. 
2. Hypothesis: According to the manuscript, families depending on subsistence farming and growing millets may have higher levels of wasting in under 5 children and malnourishment in women. Assessing causality by relating production of millets to nutritional status without looking at actual consumption and other factors [underlying and basic factors (ref to UNICEF conceptual framework of Causes of maternal and child malnutrition)] may be flawed.
3. The issue of assuming causality from correlations with perhaps erroneous conclusions is a major concern in this manuscript. It is crucial to look at dietary consumption data, from NSSO and NNMB and it is also important to adjust for all the other factors like SES, endemicity to diseases, access to potable water before superimposing millet cultivation and prevalence of malnutrition in women and wasting in children. These communities are poverty stricken and have limited access to several resources mentioned above. So the yield of crop is questionable as well. What % of their farming are millets? What % of their food plate are millets? All these need to be explored before drawing conclusions. It's not surprising that households with high coarse cereals consumption have high wasting and malnutrition. To assume that the underlying cause of wasting is coarse cereals (and that wasting would be less without coarse cereals) seems erroneous and perhaps indefensible. Comparing similar households with and without coarse cereal consumption could be a convincing way to draw conclusion in the present manuscript.
4. Why have the authors excluded Ragi (finger millets) from the analysis, which is one of the most nutritious millets?
5. With Public distribution system (PDS) , a food security program in India distributing majorly rice and wheat throughout the country at subsidized rates, it might be difficult to conclude that the millet growing communities, who have access to PDS are only consuming millets and hence have poor nutritional status?
6. The authors mention that at 6 months, the wasting in children is the highest, does the data say that disaggregated data on millet cultivation and wasting at 6 months has the most significant association? 
7. It is somewhat worrisome to see the concluding line stating that "Policies and programs targeting malnutrition need to address type of cereal consumed in order to impact childhood malnutrition in parts of India where subsistence cultivation of millets for staple consumption is prevalent." The millet consumption can actually add diversity to the Indian diets and the presence of anti-nutrients can be assessed in the light of anti-nutrient and micronutrient molar ratio (e.g. phytate: iron molar ration before concluding that the millets can lead to malnutrition.

 Recommendation: In its current state, this manuscript may be rejected.

Is the work clearly and accurately presented and does it cite the current literature?

No

If applicable, is the statistical analysis and its interpretation appropriate?

No

Are all the source data underlying the results available to ensure full reproducibility?

Yes

Is the study design appropriate and is the work technically sound?

No

Are the conclusions drawn adequately supported by the results?

No

Are sufficient details of methods and analysis provided to allow replication by others?

Yes

Reviewer Expertise:

Food systems research, assessment of nutritional status, nutrient analysis, nutritional status of indigenous communities of India

I confirm that I have read this submission and believe that I have an appropriate level of expertise to state that I do not consider it to be of an acceptable scientific standard, for reasons outlined above.

# Response

**Published:** 2020-10-09

**Authors:** Prashanth N Srinivas

**Affiliations:** Institute of Public Health Bengaluru, India

Thank you for your careful review of our submission and for raising important concerns. We have now comprehensively revised the paper and hope that the revised version has adequately responded to your concerns raised. We first provide a comprehensive overview of the main arguments underlying our revision and subsequently provide point-by-point response to the specific concerns raised. We kindly point you to review substantive responses we have made in response to reviewer 1 under the following headings which are also relevant to concerns raised by you: *(a) On protein and amino acid content of cereals, (b) On the matter of why some cereals such as millet and sorghum have lower digestibility, (c) Protein quality in the first 1000 days of conception, (d) On the micronutrient content in cereals*

**On the choice of title: **There have been  questions linked to our choice of the title by both reviewers. Overall, there are 108 districts each with higher prevalence of stunting and wasting, with an overlap of both in 18 districts (Figure 1 showing overlaps between two sets of districts in response to reviewer 1). Undeniably, cereals constitute upwards of 70% protein consumption in rural India and malnutrition has a strong rural preponderance worldwide ^1,2,3^. Looking from the prism of predominantly cereal based diets, the wide range of  protein (or amino acid) and micronutrient availabilities among cereals (as explained in our overarching response above) could well determine both wasting and stunting. Furthermore the results provided in the revised version strengthen the evidence-base for an association of wasting and low BMI with millet cultivation (as a proxy for consumption). 

**On the hypothesis: **Our hypothesis is based on the distinct patterns of higher prevalence of stunting and wasting as well as the wide diversity in the cultivation and nutritional values of both micronutrients and proteins among cereals. We agree that other factors linked to poverty like dietary diversity, infections, low birth weight could well be contributing  to malnutrition. This emphasizes is a need for well-designed studies to look for the contribution of cereals consumed to the patterns of malnutrition. But, given that some states with higher poverty (e.g., UP and Bihar) with relatively lower levels of low BMI and wasting than states in peninsular (or central) India (also see Table 1 in response to Reviewer 1 and the section titled *On the early onset of malnutrition in India and ecogeographic patterns & *section titled *On the micronutrient content of cereals*) points to other factors.

**On the exclusion of *ragi*(finger millet)**: As mentioned in the text we excluded *ragi *from the millets because of its purported nutritional richness and its distinct subfamily in the grass family *Poaceae* ^4,5,6^. This is also corroborated by patterns in the African continent where the countries which had the highest production of ragi (Uganda and Tanzania) are in the highlands of East Africa with lower prevalence of wasting when compared to other millet-growing regions in the continent ^4,7^.The figure showing the distribution of Ragi in eastern highlands is available from p.42 (Link); the geo-spatial analysis of prevalence of malnutrition in low- and middle-income countries by Kinyoki et. al figure 2 showing low prevalence of wasting in areas having pre-dominant *ragi *cultivation is available ( Link).

**About Poverty, PDS and food security in India and its effects on our findings** (see table uploaded on Figshare as tables are not allowed in response to reviewers) **: **That the Public Distribution system (PDS) is invaluable in managing food security in the country is undeniable. The share of rice or wheat consumption from PDS in different states indicates that the percentage is quite less (ranging from 7.6% in Gujarat to 34.3% in Chhattisgarh) . The percentage of improvement in PDS use from 2004-5 to 2011-12 has not led to a commensurate decline in undernutrition in the country ^8,9^. Moreover, the coverage of PDS is much higher in hill-states of northern India and in South Indian states as compared to other regions where malnutrition prevalence is higher ^9^. Hence, the likelihood of effects of PDS especially in the regions that we analyse in this paper by the marginal farmers and rural poor is likely to be low with subsistence cultivation continuing to play an important part, especially among the poorest communities. 

**About validity of data for study purposes: **The major nutrition surveys completed in this decade are summarised by Rathi et. al. (see table 1; Link to journal) ^7 ^. The Comprehensive National Nutrition Survey 2016-18 has data by state and their respective urban/rural areas and not by districts ^10^. The district level household survey 4 (DLHS4 in 2012-14) covered only 336 districts among 640 present at that time. The minimum disaggregation available on such a scale is at the district level only. The malnutrition data in our study was extracted from NFHS4 which is a nationwide survey encompassing all districts ^11,12^. The Hemalatha et al ^13^ study uses multiple data inputs with the latest being,NFHS4 and India Urban Nutrition Survey Data 2015-2016.  As seen from table 4 above taken from Rathi et al ^14^NFHS4 is the only nationwide survey encompassing all districts completed in the present decade. The food consumption data too has dietary groups with cereals aggregated in a group, as grains, roots and tubers. For the purposes of our study we needed separate consumption of cereal (by type) in each district which is not available in CNNS or NFHS4.The older NSSO (NSS 55) has data on type of cereals consumed but that too is not available by district but by 77 ecogeographic zones ^15 ^.

**Point-by-point response to review observations**

  

- Title: The title has issues in terms of including food (cereals) and nutrients (proteins and micronutrients) in the same basket, which is not clear.

Thank you for bringing the title to attention. We have explained above under the heading “ *on the choice of the title*”.

- Hypothesis: According to the manuscript, families depending on subsistence farming and growing millets may have higher levels of wasting in under 5 children and malnourishment in women. Assessing causality by relating production of millets to nutritional status without looking at actual consumption and other factors [underlying and basic factors (ref to UNICEF conceptual framework of Causes of maternal and child malnutrition)] may be flawed.

We accept that obtaining actual consumption would have been ideal for establishing or negating such a hypothesis. In the version 2 of our paper, we have attempted to include other factors pertinent to malnutrition (available through nationwide surveys) in our analysis . We have explained this in detail under the sections *On the hypothesis* and *On the validity of data for study purposes*. Please also see the more extended multivariate analysis in version 2.  

- The issue of assuming causality from correlations with perhaps erroneous conclusions is a major concern in this manuscript. It is crucial to look at dietary consumption data, from NSSO and NNMB and it is also important to adjust for all the other factors like SES, endemicity to diseases, access to potable water before superimposing millet cultivation and prevalence of malnutrition in women and wasting in children. These communities are poverty stricken and have limited access to several resources mentioned above. So the yield of crop is questionable as well. What % of their farming are millets? What % of their food plate are millets? All these need to be explored before drawing conclusions. It's not surprising that households with high coarse cereals consumption have high wasting and malnutrition. To assume that the underlying cause of wasting is coarse cereals (and that wasting would be less without coarse cereals) seems erroneous and perhaps indefensible. Comparing similar households with and without coarse cereal consumption could be a convincing way to draw conclusion in the present manuscript.

We have strengthened the analysis in the revised version as well has edited the language implying causality. We, in fact do not want to attribute causality but wish to describe a consistent pattern and provide a plausible hypothesis in the epidemiology of malnutrition in India. Hence, we have placed the pathway incorporating the mechanisms of causation in discussion rather than results in version 2 . We have incorporated few of the variables pertaining to causation  of malnutrition in a multivariate analysis as well. Dietary consumption data from nationwide surveys have limitations as mentioned under the section *About validity of data for study purposes.* We concede that well designed field studies are required to come to a clear position on this issue.

  

- Why have the authors excluded Ragi (finger millets) from the analysis, which is one of the most nutritious millets?

 

 We have dwelt on this under the above section titled *On the exclusion of ragi(finger millet).*

  

- With Public distribution system (PDS) , a food security program in India distributing majorly rice and wheat throughout the country at subsidized rates, it might be difficult to conclude that the millet growing communities, who have access to PDS are only consuming millets and hence have poor nutritional status?

This has been explained in the section above titled *About Poverty, PDS and food security in   India and its effects on our findings.*

  

- The authors mention that at 6 months, the wasting in children is the highest, does the data say that disaggregated data on millet cultivation and wasting at 6 months has the most significant association?

 

 In Fig 5 of version 1 and Figs 9 & 10 of version 2 the age profile of children in the 108 high stunting and 108 high wasting districts is plotted. The districts with higher wasting ( which have higher cultivation of millets as brought out in the results) have higher wasting at 6 months. However, data are unavailable to establish this at the household level due to lack of data on type of cereal consumed (which we have elaborated under the discussion). 

  

- It is somewhat worrisome to see the concluding line stating that "Policies and programs targeting malnutrition need to address type of cereal consumed in order to impact childhood malnutrition in parts of India where subsistence cultivation of millets for staple consumption is prevalent." The millet consumption can actually add diversity to the Indian diets and the presence of anti-nutrients can be assessed in the light of anti-nutrient and micronutrient molar ratio (e.g. phytate: iron molar ration before concluding that the millets can lead to malnutrition.

We have indeed taken note of this suggestion and changed the manuscript in version 2 accordingly. We have taken into consideration the diversity of nutritional profiles of various cereals and brought out tables (Table 3 & 4) to highlight this. Further detailed explanation has been given under the headings *On protein and amino acid content of cereals, Protein quality in the first 1000 days of conception and On the micronutrient content in cereals *in the response to reviewer 1. The concluding line as stated in the comment has been removed in version 2.

**References:**

  

1. Swaminathan S, Vaz M, Kurpad AV: Protein intakes in India. *Br J Nutr.* 2012; **108**(Suppl 2): S50–S58. PubMed Abstract | Publisher Full Text
2. Manary MJ, Heikens GT, Golden M. Kwashiorkor: more hypothesis testing is needed to understand the aetiology of oedema. Malawi Med J. 2009 Sep;21(3):106-7. doi: 10.4314/mmj.v21i3.45630. PMID: 20345018; PMCID: PMC3717490.
3. Paciorek CJ, Stevens GA, Finucane MM, Ezzati M; Nutrition Impact Model Study Group (Child Growth). Children's height and weight in rural and urban populations in low-income and middle-income countries: a systematic analysis of population-representative data. Lancet Glob Health. 2013 Nov;1(5):e300-9. doi: 10.1016/S2214-109X(13)70109-8. Epub 2013 Oct 24. PMID: 25104494; PMCID: PMC4547325.
4. National Research Council: Lost Crops of Africa. Washington DC National Academies Press; 1996. Reference Source
5. Shobana S, Krishnaswamy K, Sudha V, et al.: Finger Millet ( *Ragi, Eleusine Coracana* L.): A Review of Its Nutritional Properties, Processing, and Plausible Health Benefits. *Adv Food Nutr Res.* 2013;69:1–39. 23522794 10.1016/B978-0-12-410540-9.00001
6. Cannarozzi G, Plaza-Wüthrich S, Esfeld K, et al.: Genome and transcriptome sequencing identifies breeding targets in the orphan crop tef ( *Eragrostis tef*). *BMC Genomics.* 2014;15(1):581. 25007843 10.1186/1471-2164-15-581 4119204
7. Kinyoki, D.K., Osgood-Zimmerman, A.E., Pickering, B.V. *et al. *Mapping child growth failure across low- and middle-income countries. *Nature* **577, **231–234 (2020). https://doi.org/10.1038/s41586-019-1878-8
8. Kumar, A., Ayyappan, S. Food Security and Public Distribution System in India. *Agric Res* **3, **271–277 (2014). https://doi.org/10.1007/s40003-014-0115-9
9. NITI Ayog. Evaluation Study on Role of Public Distribution System in Shaping Household and Nutritional Security India. Available online: http://niti.gov.in/writereaddata/files/document_publication/Final%20PDS% 20Report-new.pdf.Accessed on 16 August 2020.
10. Ministry of Health and Family Welfare (MoHFW), Government of India, UNICEF and Population Council. 2019. Comprehensive National Nutrition Survey (CNNS) National Report. New Delhi. 
11. Khan J, Mohanty SK: Spatial heterogeneity and correlates of child malnutrition in districts of India. *BMC Public Health.* 2018; **18**(1): 1027. PubMed Abstract | Publisher Full Text | Free Full Text
12. NFHS: National Family Health Survey-4 (NFHS-4). *Minisitry Heal Fam Serv India.* 2016. Reference Source
13. India State-Level Disease Burden Initiative CGF Collaborators. Mapping of variations in child stunting, wasting and underweight within the states of India: the Global Burden of Disease Study 2000-2017. *EClinicalMedicine*. 2020;22:100317. Published 2020 May 13. doi:10.1016/j.eclinm.2020.100317
14. Rathi K, Kamboj P, Bansal PG, Toteja GS. A review of selected nutrition & health surveys in India. *Indian J Med Res*. 2018;148(5):596-611. doi:10.4103/ijmr.IJMR_1808_18
15. Rao PP, Birthal PS, Reddy BVS, Rai KN, Ramesh S. Diagnostics of sorghum and pearl millet grains-based nutrition in India. SAT eJournal 2006;2(1).

# Peer review: Reviewer response for version 1

**DOI:** 10.21956/wellcomeopenres.17478.r39353

**Published:** 2020-07-21

**Authors:** Edward J.M. Joy

**Affiliations:** 1 Faculty of Epidemiology and Population Health, London School of Hygiene & Tropical Medicine, London, UK

The authors assess whether staple cereal consumption patterns in India underlie malnutrition patterns. They test this through an association between District-level production of staple cereals, and prevalence of stunting/wasting in children or low BMI/short stature in adult women.

 The article is quite clearly written and I appreciate that the authors have made the datasets and analysis code available for reviewers/readers. This is commendable. 

 However, there are a number of areas that concern me about the study:

 The study title calls this an exploratory analysis, and if this is to be indexed I think the interpretation of the results and framing of the whole paper needs to be consistent with this. For example, the authors currently write "Policies and programs targeting malnutrition need to address type of cereal consumed in order to impact childhood malnutrition in parts of India where subsistence cultivation of millets for staple consumption is prevalent". I don't think such a statement is justified based on the findings of this paper, given the exploratory nature of the analysis, quite apart from the methodological shortcomings. 

 The title also specifies wasting, where 4 different anthropometric outcomes were assessed. 

 There are several methodological shortcomings to the analysis, in my opinion. The following shortcomings are noted by the authors:

1. Cereal production is not equivalent to cereal consumption, so testing the hypothesis using cereal production data is problematic. Can the authors explain why 'consumption ' data were not used, e.g. from the Comprehensive National Nutrition Survey?
2. Data are integrated at the District-level. This level of aggregation is likely to mask the true effect, if there is indeed an effect, between millet consumption and risk of malnutrition.

The following shortcomings are not reported in the paper:

- The authors propose a biochemical pathway through which consumption of millets and sorghum might negatively affect nutritional status of women and children. However, they ignore wider contextual factors that are likely to be related both to the likelihood of producing millets and to the risk of malnutrition. These factors include, for example, socioeconomic status and likelihood of drought. There are multiple pathways linking such environmental and socioeconomic factors to nutritional status, mostly operating outside specific biochemical pathways. The proposed pathways (Figure 6) should at most be included in the discussion (not results), with acknowledgement that it may explain a small proportion of the observed association...if at all. 
- The relationship between millet production and prevalence of malnutrition outcomes (displayed in Figures 3 & 4) is messy. This is not necessarily inconsistent with the hypothesis, considering the District-level nature of the data and the multiple and complex factors underlying nutritional status. However, do the authors really find sufficient evidence with appropriate statistical certainty to reject the null hypothesis, that there is no association between the independent and dependent variables? The authors need to present a more comprehensive assessment of the associations they find, including appropriate p-values. NB I found this hard to review as the statistical test of association was not specified in the methods. Relatedly, the axes in Figures 3 & 4 need units.
- As above, assuming production closely predicts consumption is a major limitation of the study. But this could be partially addressed through limiting the analysis to rural households only. This might avoid any associations being driven by highly-urban Districts where there is both low millet production and better-than-average nutritional status.
- The authors pick out particular micronutrients and amino acids which are apparently lower in concentration or bioavailability in millets than in other staple cereals, and suggest this offers a mechanism which might underlie the observed associations. I am not clear how the authors came to select these micronutrients and amino acids. There are others, for example calcium, which are present at much greater concentrations in millets (especially finger millet) than other staple cereals. The idea that a diet with greater millet consumption is less nutritious is not supported by the evidence. 

Other comments:

- Are the estimates of malnutrition prevalence consistent with others? E.g. Hemalatha et al (2020), The Lancet ^1^.

From visual analysis of the figures, it appears there are some discrepancies. The authors could discuss this. 

- The pattern of increased stunting and wasting prevalence around 12 mo of age (Figure 5) is consistent with other settings and is likely due to poor IYCF practices and high rates of diarrhoea when moving from exclusive breastfeeding to complementary feeding
- The authors could consider using a different base colour for Figure 1. The current colouring might not translate well to presentations or black and white printing. Also, the hatched areas represent areas with 'higher prevalence' of stunting or wasting, but it's not clear what they are higher than, i.e. what's the comparator.

Overall, I suggest the authors re-consider the framing of the paper and presentation of findings.

Is the work clearly and accurately presented and does it cite the current literature?

Partly

If applicable, is the statistical analysis and its interpretation appropriate?

No

Are all the source data underlying the results available to ensure full reproducibility?

Yes

Is the study design appropriate and is the work technically sound?

No

Are the conclusions drawn adequately supported by the results?

No

Are sufficient details of methods and analysis provided to allow replication by others?

Yes

Reviewer Expertise:

Dietary micronutrient assessment, population micronutrient assessment

I confirm that I have read this submission and believe that I have an appropriate level of expertise to state that I do not consider it to be of an acceptable scientific standard, for reasons outlined above.

## References

[1] : Mapping of variations in child stunting, wasting and underweight within the states of India: the Global Burden of Disease Study 2000-2017. *EClinicalMedicine* .2020;22: 10.1016/j.eclinm.2020.100317 100317 10.1016/j.eclinm.2020.100317 32510044PMC7264980

# Response

**Published:** 2020-10-09

**Authors:** Prashanth N Srinivas

**Affiliations:** Institute of Public Health Bengaluru, India

Comments of Dr Joy

 Thank you for your careful review of our submission and for raising important concerns. We have now comprehensively revised the paper and hope that the revised version has adequately responded to your concerns raised. We first provide a comprehensive overview of the main arguments underlying our revision and subsequently provide point-by-point response to the specific concerns raised.

**On protein and amino acid content of cereals** **: **Cecily D Williams, in her pathbreaking description of Kwashiorkor in 1933 commented ^1^: *“As maize is the only source of supplementary food , some amino acid or protein deficiency cannot be excluded as a cause.”* The understanding about proteins being relevant to the causation of stunting and Intrauterine growth restriction(IUGR) through the MTOR pathway has been extensively studied ^2,3,4,5^. Since proteins are absorbed through the ileum after breakdown into amino acids or short peptide chains, and, MTOR being exquisitely sensitive to amino acids, we incorporate the MTOR pathway in hypothesizing about the purported differences in wasting prevalence between northern and peninsular India (see figure 1). Further, as per the 2011 FAO Expert Consultation on Protein Quality Evaluation in Human Nutrition ^6^, it is recommended that “ *dietary amino acids be treated as individual nutrients and that wherever possible data for digestible or bioavailable amino acids be given in food tables on an individual amino acid basis*”. Due to paucity of human data it was further stated that “ *Where human data are lacking it is recommended that true ileal amino acid digestibility values from the growing pig be used, and where these data are not available from the growing laboratory rat*.” ^6 ^This consideration is underlying our use of ileal digestibility data in pig (Table 5 in the revised version). In a recent paper Swaminathan, Vaz & Kurpad while analysing protein intakes in India ^7^,highlight lower protein quality of a predominantly cereal based rural and tribal diet, particularly in pregnancy. There are small but significant differences in the  Lysine contents across cereals in their analysis (table2 in Swaminathan S et al ^7^). This is in line with earlier more global evidence-base ^8^. These differences are germane to the differences in malnutrition patterns between northern and peninsular India. 

      

 On the matter of why some cereals such as millet and sorghum have lower digestibility, Millward implicates tougher plant cell walls prevalent in millets; others have implicated anti-nutrient factors as well ^8,9,10,11^. This is corroborated by measures such as the Digestible indispensable amino acid scores(DIAAS) of cooked cereals (including rice and wheat) which are the lowest for foxtail and proso millet ^12^. Other independent analyses to determine the standardised ileal digestibility(SID) among eight cereal grains using DIAAS scores also have shown highest values for rice but the lowest for maize, rye and Sorghum ^13^. Furthermore, excess Leucine in Sorghum has been implicated in Pellagra reported among people who consume Sorghum as a staple in India ^14^. There are few studies that compare ileal digestibility across various complementary foods in India. In a comparison of standardized ileal digestibility(%) of amino acids between Sorghum, pearl millet and different varieties of corn, Sorghum was the lowest followed by pearl millet. The different corn varieties were higher than both ^15^. The digestibility of pearl millet protein  has been suspected to be less than other major grains ^16^. While evaluating the protein quality of complementary foods using dual isotope tracer method in comparing the true ileal digestibility of rice, finger millet ( *ragi*) and egg, finger millet and mung dal had the lowest ^13^. Studies are ongoing for other millets but are currently unavailable (email communication with corresponding author ^17^). 

**Protein quality in the first 1000 days of conception**. Pregnant women in fact need an additional 1, 9 and 31 gm per day in first, second and third trimester respectively ^18^. This lack of protein (and energy quality) is linked to low maternal BMI and Intrauterine growth restriction ^18^. As per the standard FAO reference source on millets and sorghum, Lysine amino acid score of pearl millet is variable (26-69) ^8^. Barnyard millet, little millet and Sorghum had the lowest Lysine scores among millets ^8^. The lower glycaemic index of millets vis-à-vis rice and wheat (which is indeed beneficial for elderly Diabetics), on the other hand for pregnant women on pre-dominant millet-based diet could contribute to low birth weight and wasting since glucose along with amino acids  is  an important upstream regulator for MTOR ^4,19^. In fact the FAO asserts that exclusive millet diets are not adequate to meet the growth requirements of infants and young children ^8^. In table 5 (of the revised version of our paper) we provide Lysine scores of cereals and their references in studies conducted in University of Illinois, Urbana-Champaign and elsewhere. The table is extracted from a compendium curated by Hans-Henrik Stein from published data.     

 Based on these arguments and evidence presented in our paper further supported by extensive published data, we submit that monotonous cereal based diets of the rural poor wherever they are not supplemented with good quality protein from other sources could drive wasting, as is seen in monotonous millet-based diets.

**On the micronutrient content in cereals: **Micronutrient availability in rice and wheat, on the contrary, is worse off than in millets (see table 6 of revised paper). This could be underlying the higher prevalence of stunting in rice and wheat growing areas of northern India (possibly related to Zinc deficiency). Review evidence too corroborates the other reviewer’s observation that high phytate and phytate to mineral molar ratios in plant based diets as contributing to deficiencies of Iron, zinc and Calcium ^20,21,22,23^. The higher Iron, Zinc and Calcium content in millets (especially pearl millet, Sorghum and other millets) could be offsetting the phytate and phenol inhibitors in comparison to rice and wheat ( Table 6 data from Indian Food Composition Table 2017 ^24^). This underlies our assertion “Lower lysine, high phytates with lowered micronutrients like zinc and iron in cereal based diet in wheat and rice growing areas with poor dietary diversity could lead to stunting” (as per caption of pathway figure in version 1; now figure 11). Zinc has a known association with linear growth and supplementation in developing countries has been beneficial in marginal gain of length ^25,26^. There has been a decline in the zinc & Iron molar ratios with phytates, in India over the last four decades, attributed to lesser consumption of millets and sorghum ^21,22^. Characterising micronutrient availabilities *in vivo *(particularly Zinc, Vitamin D, Magnesium, Phosphate, Selenium) in different cereal based meals will require state-of-the-art laboratory techniques like stable isotope studies. Iodine is another micronutrient critical for foetal and child growth ,but, unlikely to be of consequence in view of universal Iodization of common salt in India. Iodine availability of different cereals is however unavailable in IFCT 2017.

 Malnutrition prevalence related to micronutrients are available only at state level and with no district level data and have been compiled by us from different sources in table 1 below. The state-level data  do not reproduce intuitive patterns. For example, poorer states (higher poverty rates) are better off with respect to prevalence of low BMI among women in 10-19 years age group when compared with richer states which report higher coarse cereal cultivation (Rajasthan, Maharashtra, Karnataka and Gujarat highlighted in red). Similarly, lesser degree of low zinc prevalence was seen in 1-4 year age groups in Rajasthan and Maharashtra (see discussion above on better off micronutrient profile of coarse cereals) in comparison to Uttar Pradesh and Bihar. However, these patterns are not consistent (for example Karnataka which reports higher low zinc prevalence in 1-4 year age group despite having relatively high coarse cereal consumption). Nevertheless, a study comparing zinc levels among preschool children across five states of India too showed higher prevalence in Orissa(51.3%) followed by Uttar Pradesh(48,1%), Gujarat(44.2%), Madhya Pradesh(38.9%) and Karnataka(36.2%) ^27^. The latter three have a higher production of coarse cereals in comparison to others as seen in the table ( since tables are not allowed here, this table curated by us has been uploaded on figshare)

**On the early onset of malnutrition in India and ecogeographic patterns: **The timing of onset of malnutrition in India has been reported in earlier studies. The paper titled *The Asian Enigma* by UNICEF in 1996 brought it to the forefront with a discussion about higher proportion of low birth weights in India (and Bangladesh) with its linkages to womens’ health in general and maternal nutrition in particular ^28^. Cesar Victora et al ^29 ^have demonstrated this with India having the lowest weight for age Z scores at 1 month of age. This aspect was emphasized by Martorell et al in the comparisons between stunting and wasting prevalence as well as their respective age of occurrence between India and Gautemala ^30^. Figure 1 in the paper by Martorellillustrates this comparison indicating earlier onset of wasting in India.

 In our analysis the stunting prevalence comparison between the districts with high stunting and high wasting  showed a similar pattern as seen in figures 9 and 10 (in the revised version), with higher and earlier onset of wasting in the 108 high wasting districts. That is likely to be attributable to maternal nutritional factors during pregnancy, lactation and low birth weight. However, granular data on low birth weight by district in India not being available, this remains a hypothesis to be tested when better data becomes available. 

 Further evidence of ecogeographic patterning of malnutrition is also available in the landmark Lancet 2008 series on malnutrition ^31^  where it is asserted “ *Furthermore, stunting and severe wasting are not necessarily associated on a geographical or ecological basis—ie, countries with a similar stunting prevalence can have a several-fold difference in the prevalence of severe wasting.*” This phenomenon has also been written about by Cesar Victora in 1992 ^32^. What we are attempting to posit here is that, in a diverse country like India, such differences are likely to be important in addressing malnutrition. 

        

**Point-by-point response to review observations**

- The authors assess whether staple cereal consumption patterns in India underlie malnutrition patterns. They test this through an association between District-level production of staple cereals, and prevalence of stunting/wasting in children or low BMI/short stature in adult women. The article is quite clearly written and I appreciate that the authors have made the datasets and analysis code available for reviewers/readers. This is commendable. 

Thank you. 

  

- The study title calls this an exploratory analysis, and if this is to be indexed I think the interpretation of the results and framing of the whole paper needs to be consistent with this. For example, the authors currently write "Policies and programs targeting malnutrition need to address type of cereal consumed in order to impact childhood malnutrition in parts of India where subsistence cultivation of millets for staple consumption is prevalent". I don't think such a statement is justified based on the findings of this paper, given the exploratory nature of the analysis, quite apart from the methodological shortcomings. The title also specifies wasting, where 4 different anthropometric outcomes were assessed. 

There have been  questions linked to our choice of the title by both reviewers. Overall, there are 108 districts each with higher prevalence of stunting and wasting, with an overlap of both only in 18 districts (see Junaid et. al.; reference number 33). Undeniably, cereals constitute upwards of 70% protein consumption in rural India and malnutrition has a strong rural preponderance worldwide ^7^. Looking from the prism of predominantly cereal based diets, the wide range of  protein (or amino acid) and micronutrient availabilities among cereals (as explained in our overarching response above) could well determine both wasting and stunting. Furthermore the results provided in the revised version strengthen the evidence-base for an association of wasting and low BMI with millet cultivation (as a proxy for consumption). 

  

- There are several methodological shortcomings to the analysis, in my opinion. The following shortcomings are noted by the authors: Cereal production is not equivalent to cereal consumption, so testing the hypothesis using cereal production data is problematic. Can the authors explain why 'consumption ' data were not used, e.g. from the Comprehensive National Nutrition Survey? Data are integrated at the District-level. This level of aggregation is likely to mask the true effect, if there is indeed an effect, between millet consumption and risk of malnutrition.

This is an important shortcoming of the paper. However, no consumption data are currently available. Detailed explanation for this is provided in response to reviewer 2 under the heading *About validity of data for study purposes*. 

- The following shortcomings are not reported in the paper: The authors propose a biochemical pathway through which consumption of millets and sorghum might negatively affect nutritional status of women and children. However, they ignore wider contextual factors that are likely to be related both to the likelihood of producing millets and to the risk of malnutrition. These factors include, for example, socioeconomic status and likelihood of drought. There are multiple pathways linking such environmental and socioeconomic factors to nutritional status, mostly operating outside specific biochemical pathways. The proposed pathways (Figure 6) should at most be included in the discussion (not results), with acknowledgement that it may explain a small proportion of the observed association...if at all. 

We agree with the reviewer observation about the need to integrate other known co-variates of malnutrition which is now done in version 2. Furthermore as suggested the implications of our findings are moved to the discussion (figure 11 relating to plausible pathways is now moved to discussion). 

  

- The relationship between millet production and prevalence of malnutrition outcomes (displayed in Figures 3 & 4) is messy. This is not necessarily inconsistent with the hypothesis, considering the District-level nature of the data and the multiple and complex factors underlying nutritional status. However, do the authors really find sufficient evidence with appropriate statistical certainty to reject the null hypothesis, that there is no association between the independent and dependent variables? The authors need to present a more comprehensive assessment of the associations they find, including appropriate p-values. NB I found this hard to review as the statistical test of association was not specified in the methods. Relatedly, the axes in Figures 3 & 4 need units.

Thank you for these observations. We have now reported the details of our statistical analysis. Corresponding changes have also been made in erstwhile figures 3 and 4 which have now been broken up into figures 3-5 & 6-8 in the revised version along with appropriate p-values. 

  

- As above, assuming production closely predicts consumption is a major limitation of the study. But this could be partially addressed through limiting the analysis to rural households only. This might avoid any associations being driven by highly-urban Districts where there is both low millet production and better-than-average nutritional status.

The multivariate analysis has now taken into the account the effect of rural population proportion in the district. 

  

- The authors pick out particular micronutrients and amino acids which are apparently lower in concentration or bioavailability in millets than in other staple cereals, and suggest this offers a mechanism which might underlie the observed associations. I am not clear how the authors came to select these micronutrients and amino acids. There are others, for example calcium, which are present at much greater concentrations in millets (especially finger millet) than other staple cereals. 

This has now been comprehensively addressed in our overarching response above (see particularly discussion under sub-heading *On the matter of why some cereals such as millet and sorghum have lower digestibility *and *On micronutrient availability in cereals*. Please see also section *On the exclusion of ragi *in response to reviewer 2).

  

- The idea that a diet with greater millet consumption is less nutritious is not supported by the evidence. 

This has been addressed in the revisions made. Please also refer to section above titled *On micronutrient availability in cereals.*

  

- Are the estimates of malnutrition prevalence consistent with others? E.g. Hemalatha et al (2020), The Lancet ^66^.

Hemalatha et. al. ^66 ^have used multiple data sources for their analysis, of which the only comprehensive dataset that comprises district-level malnutrition data for the entire country is NFHS4 (which we have used in our anlaysis). Other datasets used in their paper do not provided district-level data for the entire country. Our estimates of malnutrition prevalence at the district level are reproducible and are consistent with analysis of NHFS4. 

  

- From visual analysis of the figures, it appears there are some discrepancies. The authors could discuss this. 

The maps have been reproduced with better contrast and legends for clarity. The few instances of lack of overlaps between millet cultivation and wasting (e.g., bajra in north India) are now discussed in the revised version. 

- The pattern of increased stunting and wasting prevalence around 12 mo of age (Figure 5) is consistent with other settings and is likely due to poor IYCF practices and high rates of diarrhoea when moving from exclusive breastfeeding to complementary feeding

We have extensively discussed the early onset of malnutrition in India above. 

  

- The authors could consider using a different base colour for Figure 1. The current colouring might not translate well to presentations or black and white printing. Also, the hatched areas represent areas with 'higher prevalence' of stunting or wasting, but it's not clear what they are higher than, i.e. what's the comparator.

Thank you for these suggestions. The revised figures address these concerns. We took the levels of districts having higher prevalence of stunting >45% and wasting >27% based on cut-offs suggested by Junaid et al article ^33^. We used levels of > = 30% and >= 15% as high prevalence of women’s BMI (<18.5) and short stature(< 145 cms) respectively.

 References:

1. Williams CD: A nutritional disease of childhood associated with a maize diet. *Arch Dis Child.*1933;8(48):423–433. 21031941 10.1136/adc.8.48.423 1975318
2. Semba RD: The Rise and Fall of Protein Malnutrition in Global Health. *Ann Nutr Metab.* 2016; **69**(2): 79–88. PubMed Abstract | Publisher Full Text | Free Full Text
3. Semba RD, Shardell M, Sakr Ashour FA, *et al.*: Child Stunting is Associated with Low Circulating Essential Amino Acids. *EBioMedicine.* 2016; **6**: 246–252. PubMed Abstract | Publisher Full Text | Free Full Text
4. Jansson T, Aye ILMH, Goberdhan DCI: The emerging role of mTORC1 signaling in placental nutrient-sensing. *Placenta.* 2012; **33**(Suppl 2): e23–e29. PubMed Abstract | Publisher Full Text | Free Full Text
5. Millward DJ. Nutrition, infection and stunting: the roles of deficiencies of individual nutrients and foods, and of inflammation, as determinants of reduced linear growth of children. *Nutr Res Rev*. 2017;30(1):50-72. doi:10.1017/S0954422416000238.
6. Dietary protein quality evaluation in human nutrition. Report of an FAO Expert Consultation. *FAO Food Nutr Pap*. 2013;92:1-66.
7. Swaminathan S, Vaz M, Kurpad AV: Protein intakes in India. *Br J Nutr.* 2012; **108**(Suppl 2): S50–S58. PubMed Abstract | Publisher Full Text
8. Food and Agriculture Organization of the United Nations: Sorghum and millets in human nutrition. David Lubin Meml Libr Cat Publ Data FAO, Rome. 1995.
9. Millward DJ. The nutritional value of plant-based diets in relation to human amino acid and protein requirements. Proceedings of the Nutrition Society. Cambridge University Press; 1999;58(2):249–60.
10. Boncompagni E, Orozco-Arroyo G, Cominelli E, et al.: Antinutritional factors in pearl millet grains: Phytate and goitrogens content variability and molecular characterization of genes involved in their pathways. Kashkush K, ed. *PLoS One.* 2018;13(6):e0198394. 29856884 10.1371/journal.pone.0198394 5983567
11. Proietti I, Frazzoli C, Mantovani A: Exploiting Nutritional Value of Staple Foods in the World’s Semi-Arid Areas: Risks, Benefits, Challenges and Opportunities of Sorghum. *Healthcare.* 2015;3(2):172–193. 27417755 10.3390/healthcare3020172 4939534
12. Han F, Han F, Wang Y, et al. Digestible indispensable amino acid scores of nine cooked cereal grains. *Br J Nutr*. 2019;121(1):30-41. doi:10.1017/S0007114518003033
13. Cervantes-Pahm SK, Liu Y, Stein HH. Digestible indispensable amino acid score and digestible amino acids in eight cereal grains. *Br J Nutr*. 2014;111(9):1663-1672. doi:10.1017/S0007114513004273
14. Gopalan C, Jaya Rao KS. Pellagra and amino acids imbalance. *Vitam Horm*. 1975;33:505-528. doi:10.1016/s0083-6729(08)60971-1 Yin, Y.-L., Gurung, Nar, K., Jeaurond, E. A., Sharpe, P. H. and de Lange, C. F. M. 2002. 
15. Digestible energy and amino acid contents in Canadian varieties of sorghum, pearl millet, high-oil corn, high-oil–high-protein corn and regular corn samples for growing pigs. Can. J. Anim. Sci. **82**: 385–391 
16. National Research Council: Lost Crops of Africa. Washington DC National Academies Press; 1996. Reference Source
17.  Nirupama Shivakumar, Sindhu Kashyap, Satvik Kishore, Tinku Thomas, Aneesia Varkey, Sarita Devi, Thomas Preston, Farook Jahoor, M S Sheshshayee, Anura V Kurpad, Protein-quality evaluation of complementary foods in Indian children, *The American Journal of Clinical Nutrition*, Volume 109, Issue 5, May 2019, Pages 1319–1327, https://doi.org/10.1093/ajcn/nqy265
18. Ghosh S. Protein Quality in the First Thousand Days of Life. *Food Nutr Bull*. 2016;37 Suppl 1:S14-S21. doi:10.1177/0379572116629259
19. Atkinson FS, Foster-Powell K, Brand-Miller JC. International tables of glycemic index and glycemic load values: 2008. *Diabetes Care*. 2008;31(12):2281-2283. doi:10.2337/dc08-1239
20. Wessells KR, Brown KH. Estimating the global prevalence of zinc deficiency: results based on zinc availability in national food supplies and the prevalence of stunting. *PLoS One*. 2012;7(11):e50568. doi:10.1371/journal.pone.0050568
21. DeFries R, Chhatre A, Davis KF, et al. Impact of Historical Changes in Coarse Cereals Consumption in India on Micronutrient Intake and Anemia Prevalence. *Food Nutr Bull*. 2018;39(3):377-392. doi:10.1177/0379572118783492
22. Smith MR, DeFries R, Chhatre A, Ghosh-Jerath S, Myers SS. Inadequate Zinc Intake in India: Past, Present, and Future. *Food Nutr Bull*. 2019;40(1):26-40. doi:10.1177/0379572118825176
23. Gibson RS, Bailey KB, Gibbs M, Ferguson EL. A review of phytate, iron, zinc, and calcium concentrations in plant-based complementary foods used in low-income countries and implications for bioavailability. *Food Nutr Bull*. 2010;31(2 Suppl):S134-S146. doi:10.1177/15648265100312S206.
24. Indian Food Composition Tables 2017 T. Longvah, R. Ananthan, K. Bhaskarachary and K. Venkaiah
25. Imdad, A., Bhutta, Z.A. Effect of preventive zinc supplementation on linear growth in children under 5 years of age in developing countries: a meta-analysis of studies for input to the lives saved tool. *BMC Public Health* **11, **S22 (2011). https://doi.org/10.1186/1471-2458-11-S3-S22
26. Millward DJ. Nutrition, infection and stunting: the roles of deficiencies of individual nutrients and foods, and of inflammation, as determinants of reduced linear growth of children. *Nutr Res Rev*. 2017;30(1):50-72. doi:10.1017/S0954422416000238.
27. Kapil U, Jain K. Magnitude of zinc deficiency amongst under five children in India. Indian J Pediatr 2011; 78 : 1069-72.
28. Ramalingaswami V, Jonson U, Rohde J: The Asian Engima, the progress of nations. 1997.
29. Victora CG, de Onis M, Hallal PC, *et al.*: Worldwide Timing of Growth Faltering: Revisiting Implications for Interventions. *Pediatrics.* 2010; **125**(3): e473–e480. PubMed Abstract | Publisher Full Text
30. Martorell R, Young MF: Patterns of Stunting and Wasting: Potential Explanatory Factors. *Adv Nutr.* 2012; **3**(2): 227–233. PubMed Abstract | Publisher Full Text | Free Full Text
31. Black RE, Allen LH, Bhutta ZA, et al. Maternal and child undernutrition: global and regional exposures and health consequences. *Lancet*. 2008;371(9608):243-260. doi:10.1016/S0140-6736(07)61690-0.
32. Victora CG. The association between wasting and stunting: an international perspective. J Nutr 1992; 122: 1105–10.
33. Khan J, Mohanty SK: Spatial heterogeneity and correlates of child malnutrition in districts of India. *BMC Public Health.* 2018;18(1):1027. 30119652 10.1186/s12889-018-5873-z 6098604
